# The efficacy and safety of MARS-PD: Meridian activation remedy system for Parkinson’s disease—A single-center, assessor and statistician-blinded, parallel-group randomized, controlled trial protocol

**DOI:** 10.1371/journal.pone.0303156

**Published:** 2024-05-06

**Authors:** Miso S. Park, SangSoo Park, Jie-Yoon Kang, In Chul Jung, HoRyong Yoo

**Affiliations:** 1 Clinical Trial Center, Daejeon Korean Medicine Hospital of Daejeon University, Daejeon, Republic of Korea; 2 Department of Cardiology and Neurology of Korean Medicine, College of Korean Medicine, Daejeon University, Daejeon, Republic of Korea; 3 Department of Oriental Neuropsychiatry, College of Korean Medicine, Daejeon University, Daejeon, Republic of Korea; Ramon Llull University: Universitat Ramon Llull, SPAIN

## Abstract

**Background:**

Parkinson’s disease (PD) patients face a substantial unmet need for disease-modifying interventions. Potential approaches such as exercise and acupuncture have been investigated to slow PD progression. To address this unmet need, we developed a novel therapeutic approach that integrates acupuncture and exercise: the Meridian Activation Remedy System for PD patients (MARS-PD). Building upon promising outcomes observed in our preliminary pilot study, where MARS-PD exhibited a large clinically important difference on the Movement Disorder Society Unified Parkinson’s Disease Rating Scale Part III (MDS-UPDRS Part III), we embark on a randomized controlled trial with the primary objective of examining the efficacy, safety, and economic impact of MARS-PD.

**Methods:**

In this single-center, assessor and statistician-blinded, parallel-group randomized controlled trial, we aim to investigate the clinical efficacy of MARS-PD through 16 interventions administered over 8 weeks in 88 PD patients. Participants will be randomly assigned to the experimental (n = 44) or control (n = 44) groups. The experimental group will receive MARS-PD intervention alongside standard care, while the control group will solely receive standard care. The intervention period spans 8 weeks, followed by a 12-week post-intervention follow-up. The primary endpoint is the change in MDS-UPDRS Part III score from baseline to the conclusion of the 8-week intervention. Secondary outcomes encompass various assessments, including MDS-UPDRS, International Physical Activity Questionnaire Short Form, Parkinson Self Questionnaire, Parkinson’s Disease Sleep Scale, Timed Up and Go test, GAITRite metrics, Functional Near-Infrared Spectroscopy measurements, smart band outcomes, gut microbiome analysis results, and iris connective tissue texture.

**Discussion:**

Previous studies by the authors have indicated MARS-PD’s safety and benefits for PD patients. Building upon this foundation, our current study aims to provide a more comprehensive and detailed confirmation of the efficacy of MARS-PD.

**Trial registration:**

cris.nih.go.kr KCT0006646 –First posted on 7 October 2021; ClinicalTrials.gov NCT05621772 –First posted on 11 November 2022.

## Introduction

### Background and rationale

Parkinson’s disease (PD), the second most prevalent neurodegenerative disorder, impacts approximately 2–3% of individuals aged 65 and older, manifesting with motor symptoms such as bradykinesia, rigidity, tremor, and postural instability [1]. However, PD is considered to exhibit the fastest growth rate among various degenerative brain diseases [2]. Furthermore, non-motor symptoms such as autonomic dysfunction, constipation, sleep disorders (including rapid eye movement (REM) sleep behavior disorder), and fatigue often precede motor symptoms, broadening the scope of individuals affected by PD when considering prodromal PD patients [3,4].

For many years before it manifests clinically, PD progresses gradually, causing damage to the patients’ multiple nervous systems, including the central, peripheral, and enteric nervous systems [5]. Moreover, people with PD experience a decline in both movement speed and muscle strength, resulting from an altered pattern of motor unit activation. Over the last fifty years, research has revealed a wide range of motor abnormalities and myofiber changes in PD patients, including hypertrophy of slow-twitch Type I myofibers and atrophy of fast-twitch Type II myofibers in various muscles [6]. Several studies have found that type I myofiber grouping in muscles, not just dopaminergic cell damage in the substantia nigra, can influence motor symptoms of PD [7–9], implying that treatments other than dopaminergic drugs, such as exercise, may also be beneficial [10].

Individuals with PD currently face unmet needs in terms of long-term management and treatment strategies, given the absence of specific disease-modifying medications or sustainable interventions available at the moment [11]. Despite a growing body of evidence supporting the disease-modifying impact of various exercise modalities on PD [12], there remains a notable lack of a structured system facilitating consistent and continuous physical activity for symptom management [13]. Meanwhile, studies have indicated that long-term acupuncture, when combined with antiparkinsonian medication, can effectively manage PD. Mizushima et al. demonstrated that acupuncture in conjunction with drug treatment over a five-year period resulted in suppressed disease progression, improved daily life functioning, and enhanced exercise capabilities compared to drug treatment alone [14]. In a network meta-analysis by Kwon et al., various acupuncture treatments, when used alongside conventional levodopa therapy for individuals with PD, exhibited superior outcomes compared to sham acupuncture or exclusive reliance on conventional levodopa therapy [15].

### Objectives

In order to address the need for an effective and sustainable intervention allowing PD patients to maintain their physical activity level over time, we devised the Meridian Activation Remedy System (MARS), integrating acupuncture and exercise. MARS was specifically designed to facilitate the delivery of optimal acupuncture and exercise treatment in hectic clinical environments. Within this framework, patients engage in 20-minute exercise concurrently with the administration of intradermal acupuncture. This integrated approach is strategically designed to amplify efficiency and effectiveness, thereby optimizing therapeutic interventions for patients within busy medical settings.

MARS involves a series of linear movements designed to evenly stimulate the body’s meridians, offering a straightforward and effective routine for PD patients in outpatient settings. The intervention, incorporating both slow (30 beats per minute (BPM)) and fast (120 BPM) movements, aims to activate both slow- and fast-twitch muscle fibers of the patients. In addition, intradermal acupuncture and motions altering the center of gravity were utilized to improve balance and proprioception of the patients [16]. We have observed the effectiveness of MARS for PD in preliminary case study [17] and observational study [18]. In the pilot observational study, MARS (16 interventions administered over 8 weeks) demonstrated a large clinically important difference on the Movement Disorder Society Unified Parkinson’s Disease Rating Scale Part III (MDS-UPDRS Part III) in PD patients (from 20.00 ± 11.78 to 8.85 ± 5.54, p = 0.003). The subsequent phase involves the initiation of a randomized clinical trial to systematically validate the efficacy of MARS. In this single-center, assessor and statistician-blinded, parallel-group randomized controlled trial, our principal aim is to thoroughly assess the efficacy of MARS for PD through a comparative analysis. This entails evaluating a group of PD patients undergoing conventional therapy in conjunction with MARS, juxtaposed with a group solely receiving conventional therapy. Additionally, we will perform safety and economic evaluation of MARS intervention.

## Materials and methods

### Study design and setting

We aim to assess both the clinical efficacy and cost-effectiveness of the Meridian Activation Remedy System for Parkinson’s Disease (MARS-PD). A single-center, assessor and statistician-blinded, parallel-group randomized controlled trial will be conducted at Daejeon Korean Medicine Hospital of Daejeon University, Daejeon, South Korea between 8^th^ April 2022 and December 2024 (i.e., the recruitment period for this study). The study will encompass 88 patients diagnosed with PD, randomly assigned to either the experimental group (undergoing MARS-PD treatment) or the control group (receiving usual care without additional intervention) in a 1:1 ratio. The experimental group will undergo the MARS-PD intervention twice a week for a duration of 8 weeks, followed by a comprehensive assessment during a 12-week follow-up visit. Conversely, the control group will not receive any additional intervention and will adhere to with their usual care for 8 weeks before participating in a follow-up assessment over the subsequent 12 weeks. This trial design is structured to elucidate the potential benefits of MARS-PD, both in terms of clinical outcomes and cost-effectiveness, providing valuable insights into its impact on PD management, utilizing an equivalence framework (assuming, as our null hypothesis, that the MARS-PD therapy and standard care are equivalent). Additionally, participants in the control group, although not receiving the MARS-PD intervention during the study period, will be eligible for compensation treatment upon successful completion of the trial. This compensation involves acupuncture or MARS-PD intervention sessions (based on the subject’s preference) twice a week, totaling one month. We anticipate that offering this compensatory treatment option may further motivate participants to adhere to the study protocols.

**Fig 1:**
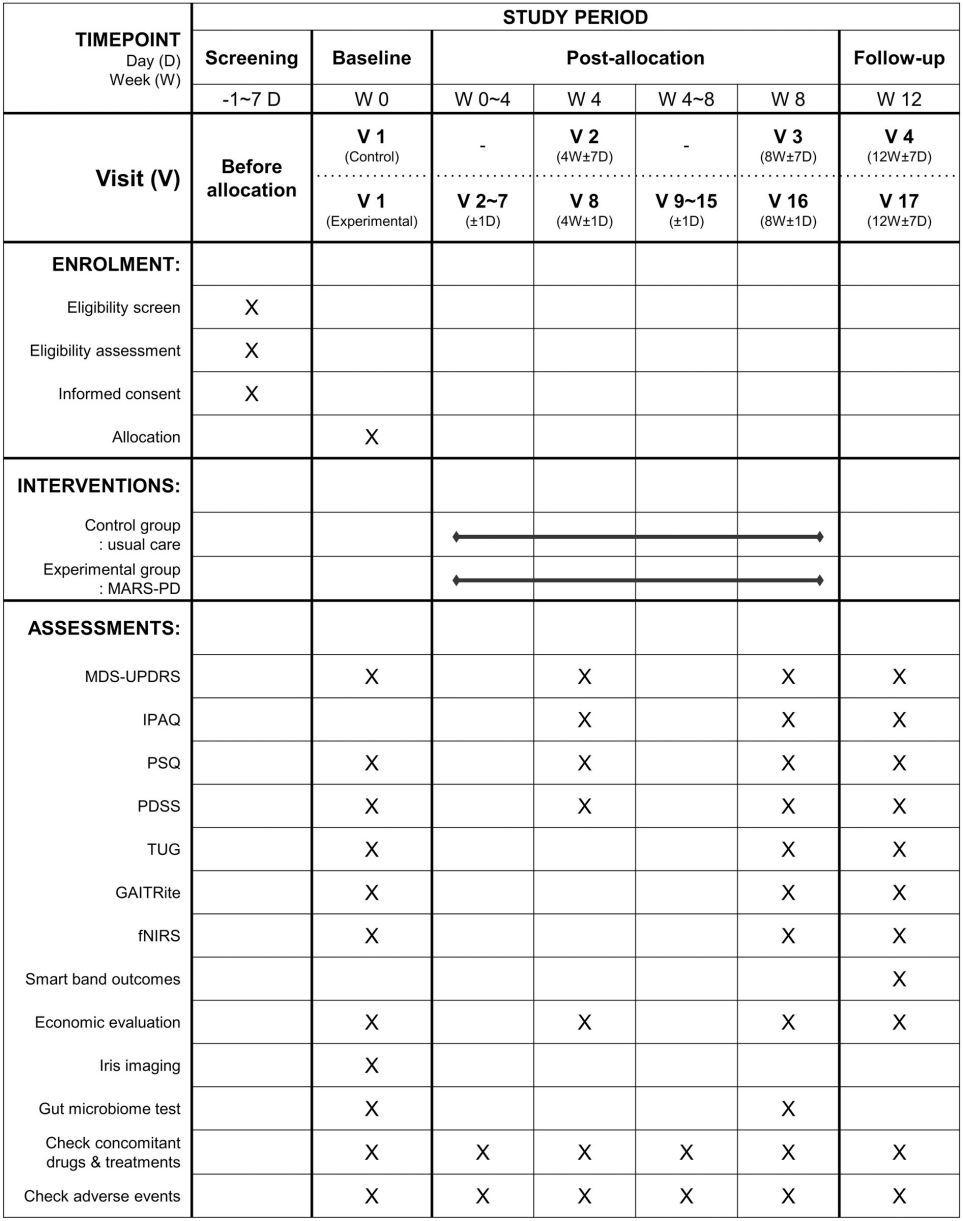
The SPIRIT schedule of enrollment, interventions, and assessments.

**Fig 2:**
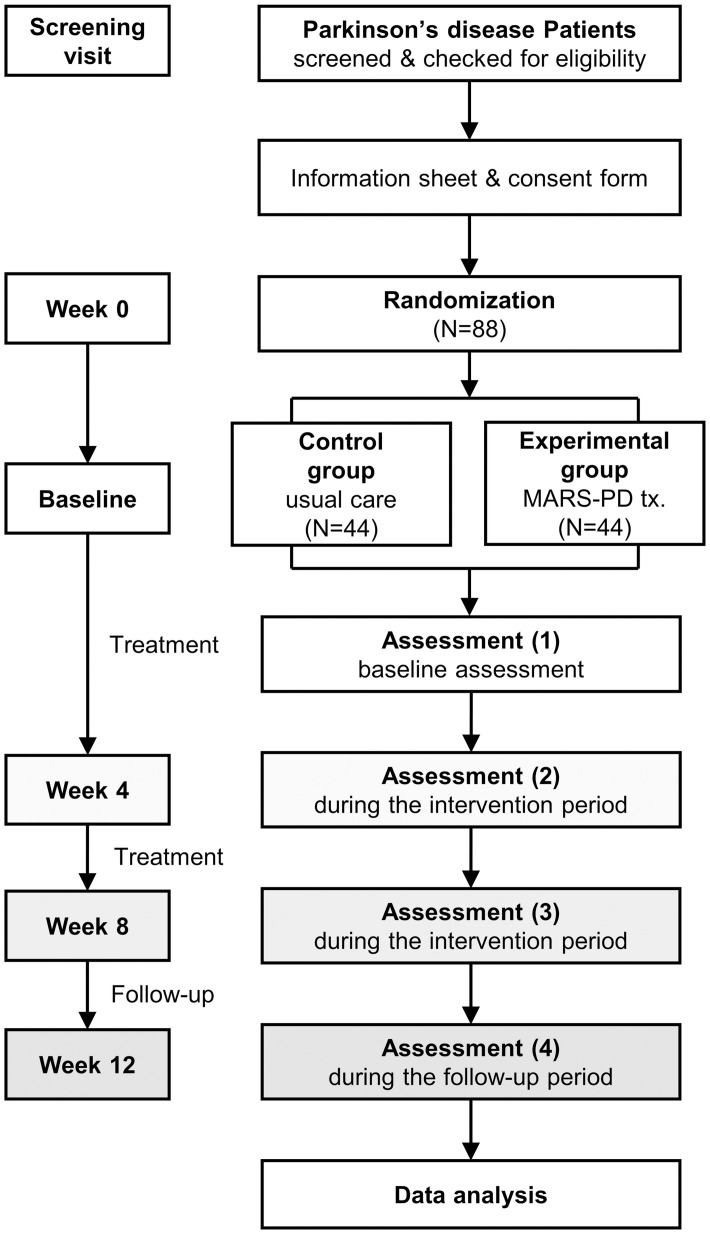
The flowchart of the study.

The protocol for this clinical trial adheres to the Standard Protocol Recommendations for Interventional Trials (SPIRIT) guidelines (S1 Appendix) [19]. The clinical trial registry number for the current manuscript can be found in CRIS (Clinical Research Information Service, cris.nih.go.kr, no.: KCT0006646) and in ClinicalTrials.gov’s Protocol Registration and Results (PRS) system (no.: NCT05621772). The SPIRIT schedule of enrollment, interventions, and assessments is shown in Fig 1. The flowchart of this study is shown in Fig 2.

### Eligibility criteria

Doctors of Korean Medicine at Daejeon Korean Medicine Hospital of Daejeon University will screen patients for eligibility. The inclusion and exclusion criteria are shown in Table 1. All participants must manually sign a written informed consent from (ICF) prior to any study procedures. During the initial visit, the doctor of Korean medicine will evaluate the Korean Mini-Mental State Examination (MMSE-K) and the Hoehn and Yahr (HY) scale. The test staff will gather vital signs, demographic information, medical and treatment history, laboratory test results, electrocardiogram readings, and chest X-ray results. Eligibility of the subjects will be ascertained through a thorough review of their history, conducted tests, and comprehensive examinations.

**Table 1:** Inclusion and exclusion criteria.

**Inclusion Criteria:** 45 to 75 years of age Patients with PD, diagnosed according to the United Kingdom Parkinson’s Diseases Society Brain Bank Criteria Hoehn and Yahr scale stage I to III Patients who have voluntarily decided to participate in the clinical study and signed the informed consent form **Exclusion Criteria:** Clinically unstable patients (e.g., elevated aspartate transaminase (AST) or alanine aminotransferase (ALT) more than three-fold the upper limit of normal in the research institute’s laboratory, heart failure, respiratory failure, etc.) Patients who are planning to undergo deep brain stimulation within the study period Pregnant or lactating women Patients with MMSE-K (Mini-Mental State Exam) score of 18 or less If there has been a change in the dosage of antiparkinsonian drugs (e.g., L-dopa, COMT (catechol-O-methyltransferase) inhibitor, Dopamine agonist, MAO-B (monoamine oxidase B) inhibitor, etc.) according to a doctor’s prescription within the last 4 weeks prior to enrollment Patients who are receiving manual therapy, exercise therapy, or rehabilitation therapy for Parkinson’s disease according to a doctor’s prescription within the last 4 weeks prior to enrollment, or are planning to receive such therapy within the study period Patients who are not suitable for participation in this clinical study according to the judgment of the researcher

### Interventions

At a 1:1 ratio, patients will be assigned to either the experimental (MARS-PD intervention) or control (usual care with no additional intervention) groups. Those in the experimental group will undergo the MARS-PD intervention twice a week for 8 a span of weeks, totaling 16 visits. The intervention will be administered by Doctors of Korean Medicine with a minimum of one year of clinical experience in the relevant field, as detailed in the authors’ previous work [16]. In contrast, patients in the control group will adhere to their usual care without any supplementary interventions. Given that the number of steps per day constitutes one of our secondary outcomes, all willing participants, regardless of their group assignment, will receive Samsung Galaxy Fit2 smart band (Samsung Electronics Co., Ltd., Suwon, South Korea), and application-based lifestyle guidance.

During the test period, participants are allowed to engage in personal exercise. However, doctor-prescribed manual therapy, exercise therapy, and rehabilitation treatment specifically tailored for PD are not permitted. This restriction aims to maintain consistency in the intervention conditions and isolate the effects of the MARS-PD being evaluated in the trial.

### Standards for concomitant drug

The utilization of antiparkinsonian drugs, such as levodopa (L-dopa), Catechol-O-methyltransferase (COMT)) inhibitor, Dopamine agonist, Monoamine oxidase B (MAO-B) inhibitor, etc., will be permitted if the dosage has remained stable for more than 4 weeks preceding the initiation of participation in the clinical trial. The prescribed drug dosage must be maintained throughout the trial period. Additionally, medications employed for the temporary treatment of conditions other than PD may be allowed after consultation with the investigator. All concomitant medications, irrespective of their purpose, should be documented with detailed drug information, including product name, dose, purpose and duration of administration, etc., in the case report forms (CRF). While there are no drugs that are pre-determined to be prohibited, the investigator retains the discretion to restrict the use of medications that may potentially influence the trial outcome, such as corticosteroids, female hormones, etc. This approach ensures thorough documentation and monitoring of medication usage throughout the study.

### Outcomes

The primary outcome of this study is the mean change in MDS-UPDRS Part III score from baseline to 8 weeks, which is a validated assessment tool is employed to evaluate the motor symptoms in PD patients [20]. MDS-UPDRS Part III scores can range from 0 to 132, with a higher score indicating worse clinical progression. Secondary outcome measures will include the MDS-UPDRS Part III score from baseline at 4 and 12 weeks, MDS-UPDRS (total) score from baseline at 4, 8, and 12 weeks, International Physical Activity Questionnaire (IPAQ) Short Form, Parkinson Self Questionnaire (PSQ, developed by researchers), Parkinson’s Disease Sleep Scale (PDSS), Timed Up and Go test (TUG) data, GAITRite Electronic Walkway Platinum metrics, Functional Near-Infrared Spectroscopy (fNIRS) measurements, smart band outcomes (number of steps per day), gut microbiome analysis results, and iris connective tissue texture and density. The details are shown in Table 2.

**Table 2:** Outcomes.

**Primary outcome measure**	**Time frame**
Movement Disorder Society Unified Parkinson’s Disease Rating Scale Part III (MDS-UPDRS Part III)	Change from baseline MDS-UPDRS Part III score at 8 weeks
**Secondary outcome measures**	**Time frame**
Movement Disorder Society Unified Parkinson’s Disease Rating Scale Part III (MDS-UPDRS Part III)	Change from baseline MDS-UPDRS Part III score at 4 and 12 weeks
International Physical Activity Questionnaire Short Form (IPAQ)	Change from baseline IPAQ score at 4, 8 and 12 weeks
Parkinson Self Questionnaire (PSQ), developed by researchers	Change from baseline Parkinson Self Questionnaire results at 4, 8 and 12 weeks
Parkinson’s Disease Sleep Scale (PDSS)	Change from baseline PDSS score at 4, 8 and 12 weeks
Timed up and go (TUG) test	Change from baseline TUG time in seconds at 8 and 12 weeks
GAITRite Electronic Walkway Platinum	Change from baseline GAITRite parameters at 8 and 12 weeks
Functional near-infrared spectroscopy (fNIRS)	Change from baseline fNIRS results at 8 and 12 weeks
Smart band outcomes (Number of steps per day)	Change from baseline daily steps at 4, 8 and 12 weeks
Movement Disorder Society Unified Parkinson’s Disease Rating Scale (MDS-UPDRS)	Change from baseline MDS-UPDRS score at 4, 8 and 12 weeks
Gut microbiome analysis results	Change from baseline at 8 weeks
Iris imaging 1 (Visible light image)	Iris connective tissue texture, baseline
Iris imaging 2 (Infrared image)	Iris connective tissue density, baseline

### Participant timeline

Among patients who applied to participate in this trial and signed the ICF, the following items will be checked for eligibility assessment: vital signs, the HY scale, the MMSE-K, demographic survey, medical and treatment history, laboratory tests, electrocardiogram, and chest X-ray. Upon random assignment to either the experimental or control group, participants will undergo treatment for a duration of 8 weeks in accordance with their respective group assignments. The MDS-UPDRS Part III, IPAQ, PSQ, PDSS, and economic evaluation questionnaire will be administered four times (0th, 4^th^, 8^th^, and 12^th^ week). TUG, GAITRite, and fNIRS measurements will be taken three times (0^th^, 8^th^, and 12th week). Gut microbiome specimens will be collected and analyzed twice: once at baseline and once at 8^th^ week. Before collecting human specimens, additional consent will be obtained for collection and use of biological specimens.

### Sample size

The hypothesis of this clinical trial is as follows:

H0 (null hypothesis): μ_t_ = μ_c_
H1 (alternative hypothesis): μ_t_ ≠ μ_c_

μ_t_ denotes the average change in MDS-UDPRS Part III score from baseline to 8 weeks in the treatment group (MARS-PD treatment), and μ_c_ denotes the average change in MDS-UDPRS Part III score from baseline to 8 weeks in the control group (usual care). The experimental and control groups will be recruited in a one-to-one ratio.

For the determination of the sample size, we referenced a previous study that closely resembled our current investigation in terms of research design, treatment period, treatment method, number of participant visits, and outcome variables [21]. In the study conducted by Hackney and Earhart, a total of 33 patients diagnosed with PD were enrolled, with 17 participants in the exercise (Tai Chi) group and 16 in the control (no intervention) group. The treatment period lasted 10–13 weeks, with a total of 20 treatment sessions. One of their outcome variables was the UPDRS motor subscale (Part III). The observed average change in UPDRS Part III scores for the treatment and control groups were -1.5 ± 6.6 and 4.3 ± 5.6, respectively. The mean difference in effect between the Tai Chi group and control group was calculated as 5.8, with a pooled standard deviation was 6.1. These parameters from the previous study served as a basis for informing the sample size calculation in our current research design.

We used a conservative value of 5 for the mean difference of the effect and 6.5 for the standard deviation to calculate the number of test subjects to confirm the effect of the experimental group compared to the control group in this study. According to Shulman et al. [22] and Sánchez-Ferro et al., [23] the minimal clinically important difference (MCID) for UPDRS Part III is 2.3 and 5, respectively. In the meantime, Horváth et al. [24] estimated that the MCID for MDS-UPDRS Part III is between 2.3 and 2.7 points. Therefore, the proposed mean difference of the effect of 5 is equal to or greater than the MCID that was previously reported by the studies. Substituting this into the calculation formula [25] results in approximately 36 people per group as shown below. Considering the dropout rate of 20%, a total of 88 clinical trial subjects, 44 per group, are required. The criteria for dropout is specified in the protocol (S2 Appendix).

$$\frac{2*{\left(Z_{1-\frac{\alpha }{2}}+Z_{1-\beta }\right)}^{2}*\sigma^{2}}{\mu_{t}-\mu_{c}}=\frac{2{\left(1.96+1.28\right)}^{2}*6.5^{2}}{5^{2}}\sim 36$$

Where α (two-tailed test significance level) = 0.05; β (type 2 error) = 0.1; 1-β (power) = 90%.

### Recruitment

Participants for the study will be recruited through diverse channels, encompassing in-hospital and subway advertisements, as well as the outpatient department of the hospital. This multifaceted recruitment strategy aims to reach a broad and representative pool of potential participants for the clinical trial.

### Assignment of interventions

The randomization process will be conducted by a statistician who is not involved in the execution or evaluation of the clinical trial. Utilizing SAS^®^ Version 9.4 (SAS Institute, Inc., Cary, NC), the statistician will employ a block randomization method to randomly assign each subject to either the experimental or control group, ensuring a 1:1 chance of selection. Following the creation of the randomization list, the statistician will seal and securely store the randomization table. Sealed envelopes will be prepared in such a way that the seal can be checked, and they will be managed separately by the research director. The test staff who provide the intervention to the patients will allocate the subjects by opening randomization envelopes in the predetermined order in front of the subjects. The opened envelopes will be stored separately, with the date of opening and the signature of the test staff written on them.

Before beginning the clinical trial, the investigator will explain the ICF to the participants and obtain written consent for the clinical trial from them based on their free will after ensuring that they understand what is expected of them. The subjects will be given a screening code once they have given their consent. Subjects who agree to take part in this clinical trial will be assigned a screening number in the manner described below.

# DJ-S-### [DJ: Daejeon Korean Medicine Hospital of Daejeon University, S: Screening, ###: Serial number (001, 002, 003, …)] (e.g., DJ-S-001: Daejeon Korean Medicine Hospital of Daejeon University’s 1st applicant for screening).

Only eligible subjects who meet the inclusion and exclusion criteria through eligibility assessment will be assigned an identification code in the manner described below.

# DJ-E-### [DJ: Daejeon University Daejeon Oriental Medicine Hospital, E: Enrollment, ###: Serial number (001, 002, 003,…)] (e.g., DJ-E-088: Daejeon Korean Medicine Hospital of Daejeon University’s 88th enrolled subject).

### Blinding

The test staff (i.e., investigator) will be divided into intervention staff and evaluation staff. The intervention staff will perform MARS-PD intervention, provide medical consultations to test subjects, and record case report forms (CRFs). Due to the nature of the intervention method used in this clinical trial, blinding of the subjects and the intervention staff will not be possible. Only blinding of the evaluation staff and statisticians will be possible in this study. To reduce the risk of unblinding caused by electronic medical records (EMRs) and CRFs, and orders will be recorded under the name of the intervention staff, not the evaluation staff. The evaluation staff will determine the research subjects’ eligibility, obtain signed ICF from the subjects, assign screening numbers and identification codes to the subjects, and perform evaluations. The evaluation staff should not be aware of the subjects’ treatment. The clinical research coordinator (CRC) will manage the subjects’ schedules and complete the CRFs as part of the basic clinical trial work. Blinding of the CRC cannot be maintained. The outcome assessor will be blinded.

### Data collection and management

The data will be collected and recorded by the intervention staff using CRF. The data will be stored confidentially in line with the National Institute for Korean Medicine Development’s (NIKOM) personal information protection policy. A copy of all documents related to this trial, including signed ICFs, CRFs, and other participant records, will be kept in document storage room for three years.

### Statistical analysis

To calculate compliance for each subject, we will use the following formula:

$$Compliance\left(\%\right)=\frac{number\;of\;interventions\;actually\;conducted}{number\;of\;interventions\;originally\;planned}$$

Throughout the trial, it is imperative that compliance (%) remains at or above 75%. Subjects falling below this threshold will be considered insufficiently compliant and subsequently excluded from the per-protocol (PP) analysis. The efficacy analysis will be primarily assessed based on the full analysis set (FAS) principle, followed by the PP principle for primary and secondary outcomes.

The FAS analysis will be carried out on cases that meet the principle of intention-to-treat (ITT). The FAS analysis will exclude cases falling into the following categories: (1) subjects not meeting the main eligibility criteria, (2) those not receiving the intervention specified in the protocol, and (3) individuals who were never evaluated after randomization with no collectable data.

On the other hand, the PP analysis will be carried out on cases that have successfully completed the entire trial process without any protocol violations. The PP analysis will exclude cases meeting any of the following criteria: (1) subjects dropping out during the intervention period, (2) violation of eligibility criteria, (3) compliance falling below 75%, and (4) any other serious violations of the clinical trial protocol.

By adopting this approach, we aim to evaluate the results conservatively, considering PD’s natural tendency to worsen over time. Statistical analysis will be conducted using the two-tailed test method, and a p-value of < .05 will be deemed statistically significant. In the event of missing values, appropriate imputation methods will be employed based on the underlying cause.

### Efficacy evaluation

The primary outcome, the mean change in MDS-UPDRS Part III score from baseline at 8 weeks between the experimental and control groups will be assessed using an independent t-test. If there is a significant difference in the baseline between the groups, we will use an analysis of covariance. If there is a significant difference in the other baseline variables, we will employ multiple regression analysis. The MDS-UPDRS Part III scores will be assessed at each time point within the confines of a linear mixed effects model with fixed effects for treatment, time, and the interaction of treatment by time, and an unstructured covariance structure for repeated measures within each subject.

The secondary outcomes include the MDS-UPDRS, IPAQ Short Form, PSQ, PDSS, TUG results, GAITRite parameters, fNIRS measurements, number of steps per day, gut microbiome analysis results, and iris connective tissue texture and density. To evaluate the secondary outcomes, we will use analysis of covariance or multiple regression analysis with corrected baseline variables. When the baseline variable is used as a covariate, a PP analysis will be performed without processing missing values.

### Safety evaluation

To determine safety, lab test results will be compared to the baseline to identify any clinical changes. AE that occurred during the study period will be listed and thoroughly explained. All SAE will be thoroughly described. AE will be gathered through patient-reported symptoms and investigator-made observations. The frequency of AE associated with and unrelated to the intervention will be recorded and presented as descriptive statistics. If necessary, additional statistical analysis will be performed based on the nature of the variables and the purpose of the evaluation.

### Economic evaluation

To confirm the cost-effectiveness of the intervention in this clinical trial versus existing treatment (usual care), an economic evaluation will be conducted concurrently with the clinical trial. The primary economic endpoint of the study is cost per Quality Adjusted Life Years (QALY) gained. To calculate QALY, quality of life will be derived from the EuroQol five dimensions questionnaire (EQ-5D) using area under the curve method [26]. The secondary economic endpoint of the study is cost per the EuroQol Visual Analogue Scale (EQ-VAS). The cost will be determined as follows: (1) the treatment cost incurred in relation to the clinical trial will be calculated by combining the number of treatments and the unit cost, and (2) the treatment cost incurred within the clinical trial institution will be recorded by the researcher by examining the EMR after unblinding.

FAS analysis will be used for economic evaluation, while PP analysis will be used to test the sensitivity of the analysis results to missing values. The primary evaluation period will be 12 weeks, which corresponds to the study’s total follow-up period. If an evaluation needs to be done after 12 weeks, the costs and effects will be extrapolated and estimated using a regression model, or a secondary analysis, such as decision modeling analysis. If the total analysis period (time horizon) exceeds 12 months, the cost unit will be unified into Korean currency (won) in 2021, and a 5% discount rate will be applied based on the Health Insurance Review and Assessment Service economic evaluation guidelines.

The findings of the economic analysis will be presented as follows: (1) a table displaying analysis results, including the incremental cost-effectiveness ratio, (2) a cost-effectiveness plane containing confidence intervals confirmed by non-parametric methods, (3) the Cost Effectiveness Acceptability Curve (CEAC), which measures cost-effectiveness sensitivity to changes in national thresholds, and (4) a graph displaying the value of information analysis results, which can be used to estimate the value of information for the target population group.

The level of statistical significance will be tested using a p-value of .05 or less. For the analysis, Stata-MP version 14 (StataCorp LLC, College Station, Texas, USA) and R version 4.0.2 (R Foundation for Statistical Computing, Vienna, Austria) will be used. TREEAge Pro 2016 (TreeAge Software Inc., Williamstown, MA, USA) will be used for extrapolation through modeling, when necessary.

### Monitoring

The academic contract research organization (A-CRO) of the Clinical Trial Center, Daejeon Korean Medicine Hospital of Daejeon University, which is independent from the investigators and sponsor, and has no competing interests will form the data monitoring committee (DMC). The DMC will supervise the progress of the clinical trial and will review and confirm whether the trial is being conducted and recorded in accordance with the protocol, standard operating instructions, clinical trial management standards, and related regulations on a regular basis. Phone calls and visits will be employed for the monitoring process. Upon visit, the monitoring staff will evaluate the trial progress and inspect the original records of test subjects and data storage. Following a monitoring session, the investigators will receive a follow-up letter in 2 days. The monitoring report will be completed in seven days, reviewed, and approved in four days.

### Adverse events

Adverse events (AEs) refer to any harmful and unintended signs, symptoms, or diseases that occur in test subjects who have received a clinical trial intervention and do not necessarily have a causal relationship with the relevant intervention method. Serious adverse events (SAEs) refer to any of the following AEs that occur as a result of clinical trial intervention: (1) when death or a threat to life occurs, (2) when hospitalization is required or the hospitalization period must be extended, (3) when there is permanent or serious disability or functional decline. The subject who has developed an AE must be monitored by the test staff until the symptoms have subsided and his or her condition has stabilized. If there is a request from the client, a report on the progression of the AE must be submitted.

The principal investigator (PI) must explain to the test staff, subjects, and their guardians all possible AEs that may occur and train them to report any phenomena that appear after the intervention. All SAEs that occur during the study period must be reported to the PI within 24 hours, regardless of whether they are related to the intervention or not. If an SAE occurs, the PI must inform the client (Daejeon Korean Medicine Hospital of Daejeon University) promptly and provide comprehensive details in writing within 5 days. Clinical trials must be halted until further instructions are given. All serious and unexpected AEs must be disclosed immediately to other relevant investigators, the Institutional Review Board (IRB), and the Ministry of Food and Drug Safety (MFDS) within the specified time frame: (1) within 7 days of notification to the client for death or life-threatening AE, with additional detailed information provided within 8 days of the initial report date; (2) within 15 days of notification to the client for all other serious or unexpected AE. SAE must be documented in the report, and the following information should be included in the final report, if possible: data on the occurrence of the SAE, its severity, treatment, course, and causal relationship with the intervention.

### Ethics and dissemination

This study’s protocol was approved by the Institutional Review Board of Daejeon Korean Medicine Hospital (DJDSKH-21-BM-11), and it has been registered on CRIS (no.: KCT0006646) and ClinicalTrials.gov’s PRS (no.: NCT05621772). The details of the trial will be communicated to all participants. Prior to enrollment, all participants will be required to submit a written ICF.

### Study protocol modifications

When modifying this protocol, the date, details, and reason for revision must be reported to and approved by the IRB. If the protocol is modified, the study participants will be informed.

## Discussion

PD is becoming a global health concern, and it imposes a significant individual, social, and economic burden [2,27,28]. PD is a neurodegenerative disorder with a high prevalence, particularly among the elderly, and the number of patients is rapidly increasing as the global population ages. Furthermore, even after adjusting for age, the prevalence of PD continues to rise, particularly in developed countries with a high sociodemographic index [28]. Although the mechanism is unknown, smoking has been linked to a lower risk of PD. Because smoking prevalence is declining in many countries, the future incidence of PD may be higher than previously estimated [29].

PD is a movement disorder characterized by the progressive degeneration of dopaminergic neurons in the substantia nigra pars compacta. The primary treatment option for PD is dopamine-based drug therapy [30]. Early initiation of dopaminergic therapies may alleviate symptoms, reduce disability, and improve quality of life in PD patients, but it has no disease-modifying effect. Complications of long-term levodopa therapy, such as motor fluctuations and dyskinesia, are also a source of concern [31].

There is an increasing demand for the development of new therapeutic approaches because there is currently no treatment that can reverse the primary pathology of PD. In a survey of PD patients, 94 out of 123 patients (76%) used complementary and alternative therapies such as acupuncture and herbal medicine to relieve motor symptoms, fatigue, pain, and constipation [32]. Furthermore, the importance of exercise in PD patients is becoming more widely recognized. Several systematic reviews and meta-analyses have found that exercise interventions such as resistance training, endurance training, balance training, and aerobic exercises can improve motor symptoms, muscle strength, cardio-respiratory fitness, gait performance, and balance in people with PD [33–35].

Acupuncture [36–38] and exercise [39,40] have both been shown in recent studies to have potential neuroplastic and neuroprotective effects in PD Prior to this study, we established a complex therapy (MARS-PD) to enhance the synergistic effects of acupuncture and exercise [16]. The goal of this randomized controlled trial is to assess MARS-PD’s clinical efficacy, safety, and cost-effectiveness. Because the goal of this trial is to assess the add-on effect of MARS-PD, we will use usual care as the comparator. Previous case study [17] and observational study [in review] by the authors found MARS-PD to be safe and beneficial to Parkinson’s disease patients. This study, we believe, will enable us to verify the efficacy of MARS-PD in greater detail.

## Supporting information

S1 Appendix SPIRIT checklist. (PDF)

S2 Appendix The institutional review board-approved protocol. (Korean & English translation).
(PDF)

## Abbreviations

A-CRO: Academic Contract Research Organization
AEs: Adverse Events
ALT: ALanine Transaminase
AST: ASpartate Transaminase
BPM: Beats Per Minute
CEAC: Cost Effectiveness Acceptability Curve
COMT: catechol-O-MethylTransferase
CRF: Case Reporf Forms
CRIS: Clinical Research Information Service
DMC: Data Monitoring Committee
EMR: Electronic Medical Records
EQ-5D: EuroQol Five Dimensions
EQ-VAS: EuroQol Visual Analogue Scale
FAS: Full Analysis Set
fNIRS: Functional Near-InfraRed Spectroscopy
HY: Hoehn and Yahr
ICF: Informed Consent Form
IPAQ: International Physical Activity Questionnaire
IRB: Institutional Review Board
L-dopa: Levodopa
MAO-B: MonoAmine Oxidase B
MARS-PD: Meridian Activation Remedy System for Parkinson’s Disease
MDS-UPDRS: Movement Disorder Society-sponsored revision of the Unified Parkinson’s Disease Rating Scale
MFDS: Ministry of Food and Drug Safety
MMSE: Mini-Mental State Exam
NIKOM: National Institute for Korean Medicine Development
PD: Parkinson’s Disease
PDSS: Parkinson’s Disease Sleep Scale
PI: Principal Investigator
PP: Per-Protocol
PSQ: Parkinson Self Questionnaire
QALY: Quality Adjusted Life Years
REM: Rapid Eye Movement
SAEs: Serious Adverse Events
SPIRIT: Standard Protocol Recommendations for Interventional Trials
TUG: Timed Up and Go
