# Supplementary material for: The efficacy and safety of MARS-PD: Meridian activation remedy system for Parkinson’s disease—A single-center, assessor and statistician-blinded, parallel-group randomized, controlled trial protocol
Source: PLoS One. 2024 May 6;19(5):e0303156. doi: 10.1371/journal.pone.0303156 (PMC11073687; doi:10.1371/journal.pone.0303156)
Supplement: S2 Appendix — (Korean & English translation). (PDF) [file pone.0303156.s002.pdf]

PROTOCOL

- 임상시험계획서 -

파킨슨병에 대한 경근 활성 치료 시스템의 평가를 위한 단일기관, 평가자 맹검, 평행설계 무작위 대조 임상시험

MARS-PD: Meridian Activation Remedy System for Parkinson's Disease — A Single-center, Rater-blinded, Parallel Randomized Controlled Trial

Protocol No.	DJ_PD_21_02
**Protocol Ver.**	**Ver. 1.6**
**임상시험 의뢰기관**<br>Requesting agency	**대전대학교 대전한방병원**<br>Daejeon Korean Medicine Hospital of Daejeon University
**임상시험 실시기관**<br>Executing agency	**대전대학교 대전한방병원**<br>Daejeon Korean Medicine Hospital of Daejeon University
**시험책임자**<br>Principal investigator	**유호룡**<br>Horyong Yoo
**발행 일자**<br>Date	**2023.10.17. (v1.6)**

개요 (Synopsis)

임상시험 제목<br>Clinical trial title	[국문] 파킨슨병에 대한 경근 활성 재활 시스템의 평가를 위한 단일기관, 평가자 맹검, 평행설계 무작위 대조 임상시험<br>[English] MARS-PD: Meridian Activation remedy system for Parkinson's Disease — A Single-center, Rater-blinded, Parallel Randomized Controlled Trial
임상시험 목적<br>Clinical trial purpose	파킨슨병 환자의 안정 시 떨림, 서동, 근육 경직, 자세 반사 장애, 보행장애 등의 운동증상과 변비, 자율신경 장애, 우울 등의 비운동증상에 대한 경근 활성 재활 시스템(MARS-PD)의 안전성, 유효성 및 경제성을 평가한다.<br>The purpose of this study is to assess the clinical efficacy and cost-effectiveness of MARS-PD. Because the goal of this trial is to assess the add-on effect of MARS-PD, we will use usual care as the comparator. Our hypothesis is that complex therapy (MARS-PD) will alleviate motor and nonmotor symptoms, improve gait performance, and enhance neuroplasticity in Parkinson's disease (PD) patients while remaining safe and cost-effective.
임상시험 실시기관 이름 & 주소<br>Name&address of clinical trial executing agency	대전대학교 대전한방병원. 대전광역시 서구 대덕대로 176번길 75.<br>전화번호: 042-470-9131<br>Daejeon Korean Medicine Hospital of Daejeon University. 75, Daedeok-daero 176beon-gil, Seo-gu, Daejeon.<br>Phone: 042-470-9131
임상시험 책임자<br>Clinical trial principal investigator	유호룡 대전대학교 한의학과 한방내과학 교수<br>HoRyong Yoo, Professor of Internal Korean Medicine, Daejeon University department of Korean Medicine
대상질환<br>Target disease	파킨슨병<br>Parkinson’s disease
임상시험 기간<br>Clinical trial duration	본 연구의 기간은 본 연구계획서에 대한 기관생명윤리위원회(IRB) 승인일로부터 39개월로 한다. 단, 피험자 등록에 따라 단축되거나 연장될 수 있다.<br>This study will last 39 months from the date this research plan was approved by the institutional review board (IRB). However, depending on the subject registration, it may be shortened or extended.
임상시험 디자인<br>Clinical trial design	단일기관, 무작위배정, 평가자 맹검, 평행설계 임상시험<br>Single-center, randomized, evaluator-blinded, parallel clinical trial
대상자 선정기준<br>Inclusion criteria	1) 만 45세 이상 75세 이하의 남녀<br>2) 영국 파킨슨병학회 뇌은행 진단 기준(UK Parkinson’s Diseases Society Brain Bank Criteria)에 따라 파킨슨병을 진단받은 자<br>3) 혼-야의 분류법(Hoehn and Yahr Scale) 중 1~3단계에 해당하는 자<br>4) 연구대상자 본인이 자의로 임상연구에 참여를 결정하고 동의서에 서명한 자<br>1) 45 to 75 years of age<br>2) Patients with PD, diagnosed according to the UK Parkinson’s Diseases Society Brain Bank Criteria

	3) Hoehn and Yahr scale stage I to III<br>4) Patients who have voluntarily decided to participate in the clinical study and signed the informed consent form
대상자 제외기준<br>Exclusion criteria	1) 심각하게 불안정한 상태인 경우 (예) AST 또는 ALT 혈중치가 연구기관 정상 상한치의 3배 이상인 경우, 심부전증, 호흡부전증 등)<br>2) 연구 기간 내에 심부 뇌자극술을 계획 중인 환자<br>3) 임산부 또는 수유부<br>4) MMSE-K(Mini-Mental State Exam) 점수가 18점 이하인 경우<br>5) 최근 4주 이내 의사 처방에 따른 항파킨슨약물(예: L-dopa, COMT inhibitor, Dopamine agonist, MAO-B inhibitor 등)의 복용량 변화가 있는 경우<br>6) 최근 4주 이내 의사의 처방에 따라 파킨슨병에 대한 도수치료, 운동치료, 재활치료를 받고 있거나, 또는 연구 기간 내에 해당 치료를 계획 중인 환자<br>7) 연구자의 판단에 따라 본 임상연구 참가가 적합하지 않은 경우<br>1) Clinically unstable patients (e.g., elevated aspartate transaminase(AST) or alanine aminotransferase(ALT) more than three-fold the upper limit of normal in the research institute's laboratory, heart failure, respiratory failure, etc.)<br>2) Patients who are planning to undergo deep brain stimulation within the study period<br>3) Pregnant or lactating women<br>4) Patients with MMSE-K(Mini-Mental State Exam) score of 18 or less<br>5) If there has been a change in the dosage of antiparkinsonian drugs (e.g., L-dopa, COMT inhibitor, Dopamine agonist, MAO-B inhibitor, etc.) according to a doctor's prescription within the last 4 weeks prior to enrollment<br>6) Patients who are receiving manual therapy, exercise therapy, or rehabilitation therapy for Parkinson's disease according to a doctor's prescription within the last 4 weeks prior to enrollment, or are planning to receive such therapy within the study period<br>7) Patients who are not suitable for participation in this clinical study according to the judgment of the researcher
방문일정<br>Visit schedule	총 12주 동안 17회 방문[screening(7일 이내), 8주간 주 2회 방문, 4주 후 관찰방문]<br>A total of 17 visits over 12 weeks [screening (within 7 days), visits twice a week for 8 weeks, observation visit after 4 weeks]
임상시험 대상자의 수 및 산정 근거<br>Number of clinical trial subjects and calculation method	1) 대상자 수: 시험군 44명, 대조군 44명, 총 88명<br>2) 산출 근거:<br>① 본 임상시험의 가설은 다음과 같다.<br>- H0(귀무가설): $\mu t = \mu c$ vs. H1(대립가설): $\mu t \neq \mu c$<br>- μt : 치료군(운동+침치료)의 베이스라인(baseline) 대비 8주 시점의 UDPRS Part III 점수 평균 변화량<br>- μc : 대조군의 베이스라인(baseline) 대비 8주 시점의 UDPRS Part III 점수 평균 변화량<br>- 평가변수의 통계적 가설검정: 양측 검정<br>- 유의수준(α): 0.05

- 제2종 오류(β): 0.1, 검정력(1-β): 90%
- 시험군과 대조군의 비율은 1:1로 동일하게 한다.

② 본 연구와 연구 디자인, 처치의 기간, 방법 및 횟수, 평가변수 등이 가장 유사한 선행연구(Hackney & Earhart, 2008)를 참고하여 계산하였다.

- 해당 논문에서는 운동치료군 17명, 대조군 16명으로 총 33명 대상자 모집하였다. 처치 기간은 10-13주였으며, 총 처치 횟수는 20회였다. 1차 유효성 평가변수는 MDS-UPDRS Part III를 사용하였다. 치료군과 대조군의 MDS-UPDRS Part III의 변화량에 대한 평균(표준편차)는 각각 –1.5(6.6)과 4.3(5.6)로 나타났으며, 대조군 대비 치료군(운동)의 효과 차이(Mean Difference)는 5.8이고, 합동표준편차(Pooled SD)는 6.1로 나타났다.

③ 효과 차이는 5, 표준편차는 6.5로 보수적인 값을 설정하여 대조군 대비 치료군(운동+침치료)의 효과를 확인하기 위한 시험대상자 수를 산출하는 경우 결과는 아래와 같이 군당 약 36명이 필요한 것으로 나타난다. 중도탈락률 20%를 고려하면 군당 44명씩 총 88명의 임상시험 대상자가 필요한 것으로 나타난다.

산출 공식:

$$\left\{\frac{2\left(z_{1-\frac{\alpha}{2}}+z_{\beta}\right)^{2}*\sigma^{2}}{\left|\mu_{T}-\mu_{c}\right|}\right\}=\left\{\frac{2(1.96+1.28)^{2}*6.5^{2}}{5^{2}}\right\}=35.515\approx 36$$

중도탈락률 20%를 고려: 35.515/0.8=44.39375, 약 44명, 총 88명.

④ 참고 논문: Hackney, Madeleine E., and Gammon M. Earhart. "Tai Chi improves balance and mobility in people with Parkinson disease." Gait & posture 28.3 (2008): 456-460.

1) Number of subjects: experimental group (n=44), control group (n=44), total (n=88)
2) Calculation basis:

① The hypothesis of this clinical trial is as follows.

- H0 (null hypothesis): μt = μc; H1 (Alternative hypothesis): μt ≠ μc
- μt: Average change in UDPRS Part III score at 8 weeks compared to the baseline of the treatment group (exercise + acupuncture & usual care)
- μc: Average change in UDPRS Part III score at 8 weeks compared to the baseline of the control group (usual care only)
- Statistical hypothesis test of evaluation variables: two-tailed test
- Significance level (α): 0.05
- Type 2 error (β): 0.1, power (1-β): 90%
- The ratio of experimental group and control group is 1:1.

② The calculation was based on a previous study (Hackney & Earhart, 2008) that was most similar to this one in terms of research design, treatment period, method and number of treatments, and evaluation variables.

A total of 33 subjects were recruited for Hackney & Earhart's study: 17 for the exercise therapy group and 16 for the control group. The treatment period lasted 10-13 weeks, with 20 treatment sessions. MDS-UPDRS Part III was the primary efficacy endpoint. The treatment and control groups' average (standard deviation) changes

<table>
<tr><td></td><td>in MDS-UPDRS Part III were -1.5 (6.6) and 4.3 (5.6), respectively, and the mean difference in the effect of the treatment group (exercise) compared to the control group was 5.8. Additionally, the pooled standard deviation (Pooled SD) was 6.1.
③ When the number of test subjects required to confirm the effect of the treatment group (exercise + acupuncture) compared to the control group is calculated using a conservative value of 5 for the effect difference and 6.5 for the standard deviation, the results show that approximately 36 people are required per group, as shown below. Considering a 20% dropout rate, a total of 88 clinical trial subjects, 44 per group, are required.
Calculation formula:
$$\left\{\frac{2\left(z_{1-\frac{\alpha}{2}}+z_{\beta}\right)^{2}*\sigma^{2}}{\left|\mu_{T}-\mu_{c}\right|}\right\}=\left\{\frac{2(1.96+1.28)^{2}*6.5^{2}}{5^{2}}\right\}=35.515\approx 36$$
Considering a 20% dropout rate: 35.515/0.8=44.39375, approximately 44 subjects per group are required (a total of 88 subjects).
④ Reference paper: Hackney ME, Earhart GM. Tai Chi improves balance and mobility in people with Parkinson disease. Gait & posture. 2008;28(3):456-60.</td></tr>
<tr><td>시험 방법
Test Methods</td><td>1) 중재 기간 및 간격
지원자 중에서 임상시험에 대한 충분한 설명을 듣고 동의서에 서명한 후 선정기준 및 제외기준에 따라 본 임상시험에 참여하게 되는 대상자에게 대상자 식별코드를 부여하고, 시험군과 대조군으로 나누어 임상시험을 실시한다. 시험군에 속하는 대상자는 screening 후 8주간 총 16회(주 2회) 시험기관을 방문하여 파킨슨병에 대한 경근 활성 재활 시스템으로 치료를 받고, 시험 시작 전(baseline)과 4주, 8주, 12주에 유효성, 안전성 및 경제성을 평가하기 위한 검사를 받는다. 대조군에 속하는 대상자는 baseline 방문 시에 유효성, 안전성 및 경제성을 평가하기 위한 검사를 동일하게 받고, 4주, 8주, 12주에 시험기관을 방문하여 재검사를 받는다.
2) 시험군: 파킨슨병에 대한 경근 활성 재활 시스템(MARS-PD) (8주 동안 16회, 주 2회 실시)
3) 대조군: 통상적 치료(usual care, 기존 복용하던 병용약물 및 병용치료(한의 치료 제외)는 허용하며, 본원에서 그 이외의 추가적인 중재를 시행하지 않음) 및 생활 습관에 대한 조언 (시험군 및 대조군 모두 스마트 밴드를 통한 기본적인 생활 관리를 제공함.)
4) 다른 처치: 시험 기간 동안 파킨슨병에 대해 한의 치료를 제외한 약물, 비약물 요법 등 모든 병용치료를 허용함. 단, 약물을 복용하는 경우에는 최근 4주 동안 의사 처방에 따른 항파킨슨 약물의 복용량이 일정해야 하고 시험 기간 동안 역시 일정하게 유지해야 함. 복용하고 있는 약물이나 다른 치료 및 시험 도중 변경되는 약물이나 치료에 대해서는 연구자가 확인하여 기록함. 또한, 시험 기간 동안에는 개인적으로 수행하는 운동은 허용하나, 의사의 처방에 따른 파킨슨병에 대한 도수치료, 운동치료, 재활치료는 허용하지 않음.
5) 진단 및 시술자의 요건: 한방내과 전문의, 석사, 박사 또는 해당 과정을 연수 중에 있는 한의사로 1년 이상의 해당 분야 임상경험이 있는 자
1) Intervention period and interval
Those who will participate in this clinical trial are assigned a subject</td></tr>
</table>

	identification code based on the inclusion and exclusion criteria after receiving a sufficient explanation of the clinical trial and signing the consent form. The clinical trial will be conducted by dividing them into an experimental group and a control group. After screening, subjects in the experimental group will visit the test site 16 times (twice a week) and receive MARS-PD. Before beginning the trial (baseline), examinations will be performed to assess efficacy, safety, and economic feasibility, as well as at 4, 8, and 12 weeks. At the baseline visit, subjects in the control group will be subjected to the same tests to assess effectiveness, safety, and economic feasibility, and they will also return to the test site at 4, 8, and 12 weeks to be re-examined.<br>2) Experimental group: MARS-PD (16 times for 8 weeks, twice a week)<br>3) Control group: Usual care (usual care, existing concomitant medications, and concomitant treatments (excluding Korean Medicine treatment) are permitted, and no further intervention will be performed at our hospital) and lifestyle advice (both experimental and control groups may receive basic lifestyle guidance via smart band).<br>4) Other treatments: All Parkinson's disease combination treatments, including drug and non-drug treatments (except Korean medicine treatment), are permitted during trial participation. If taking anti-Parkinson medication, the dosage prescribed by a doctor must have been consistent for the previous four weeks and must remain constant during the test period. The researcher confirms and records any medications or other treatments that are being taken, as well as any medications or treatments that change during the test. Personal exercise is permitted during the test period, but doctor-prescribed manual therapy, exercise therapy, and Parkinson's disease rehabilitation treatment are not.<br>5) Korean medicine internal medicine specialist, holder of a master's or doctorate in the relevant field, or doctor of Korean medicine, all with at least one year of clinical experience in the relevant field.
Efficacy endpoint	1) 일차 유효성 평가변수(Primary Outcomes measure)<br>① MDS-UPDRS Part Ⅲ: 시험 시작 전과 8주 후 MDS-UPDRS Part Ⅲ 점수의 변화<br>2) 이차 유효성 평가변수(Secondary Outcome measures)<br>① MDS-UPDRS Part III: 시험 시작 전(baseline)과 4주, 12주 후 MDS-UPDRS Part III 점수의 변화<br>② MDS-UPDRS: 시험 시작 전(baseline)과 4주, 8주, 12주 후 MDS-UPDRS 점수의 변화<br>③ IPAQ: 시험 시작 전(baseline)과 4주, 8주, 12주 후 IPAQ 점수의 변화<br>④ 파킨슨 자가 검사지(PSQ): 시험 시작 전(baseline)과 4주, 8주, 12주 후 파킨슨 자가 검사지 점수의 변화<br>⑤ PDSS: 시험 시작 전(baseline)과 4주, 8주, 12주 후 PDSS 점수 총점의 변화<br>⑥ TUG: 시험 시작 전(baseline)과 8주, 12주 후 TUG 점수의 변화<br>⑦ GAITRite: 시험 시작 전(baseline)과 8주 12주 후 GAITRite 측정값의 변화<br>⑧ fNIRS: 시험 시작 전(baseline)과 8주, 12주 후 fNIRS 측정값의 변화

⑨ 스마트밴드**: 시험 시작 전(baseline)과 4주, 8주, 12주 갤럭시 핏2를 이용하여 측정한 걸음수, 수면시간, 수면패턴 등의 변화
⑩ 홍채검사 1 & 2: 홍채검사를 통한 홍채의 모양과 구조
⑪ 장내미생물 (baseline)과 8주 후 미생물별 비율 변화량

3) Cost-effectiveness outcome measures
① QALYs: 총 연구기간(12주)에 대한 QALYs
② EQ-5D: 시험 시작 전(baseline)과 4주, 8주, 12주 후 EQ-5D 점수의 변화
③ EQ-VAS: 시험 시작 전(baseline)과 4주, 8주, 12주 후 EQ-VAS 점수의 변화
④ 직접의료비, 직접비의료비, 간접비용
⑤ ICER(cost per QALYs)
⑥ NMB(Net monetary benefit)
⑦ CEAC(Cost effectiveness acceptability curve)

* Baseline: 시험 시작 전
4, 8주(Treatment phase): 시험 시작 후 4, 8주 후
12주(Follow-up phase) : 시험 시작 후 12주 후(추적관찰)
** 삼성 갤럭시 핏2(Samsung Galaxy Fit2) 및 삼성 갤럭시 핏(Samsung Galaxy Fit) 어플리케이션(갤럭시, iOS 공통)을 활용한다.
*** 스마트밴드는 착용을 원하는 대상자에게 선택적으로 제공하며, 연구 완료자에 한하여 12주차에 데이터를 일괄 수집한다. 단, 중도탈락자의 경우 데이터를 수집하지 않는다.

1) Primary outcome measure
① MDS-UPDRS Part Ⅲ: Change in MDS-UPDRS Part Ⅲ score before and 8 weeks after starting the test

2) Secondary outcome measures
① MDS-UPDRS Part III: Change in MDS-UPDRS Part III score before starting the test (baseline) and after 4 and 12 weeks
② MDS-UPDRS: Change in MDS-UPDRS score before starting the test (baseline) and after 4, 8, and 12 weeks.
③ IPAQ: Change in IPAQ score before starting the test (baseline) and after 4, 8, and 12 weeks.
④ PSQ: Change in PSQ score before starting the test (baseline) and after 4, 8, and 12 weeks.
⑤ PDSS: Change in total PDSS score before starting the test (baseline) and after 4, 8, and 12 weeks.
⑥ TUG: Change in TUG score before starting the test (baseline) and after 8 and 12 weeks
⑦ GAITRite: Changes in GAITRite measurements before starting the test (baseline) and after 8 and 12 weeks
⑧ fNIRS: Changes in fNIRS measurements before starting the test (baseline) and after 8 and 12 weeks.
⑨ Smart Band**: Changes in step count, sleep time, sleep pattern, etc. measured using Galaxy Fit 2 before the start of the test (baseline) and at 4, 8, and 12 weeks.
⑩ iris examination 1 & 2: Shape and structure of the iris through iris examination
⑪ Gut microbiome (baseline) and percentage change by

	microorganism after 8 weeks<br><br>* Baseline: Before the test begins<br>Weeks 4 and 8 (Treatment phase): 4 and 8 weeks after starting the test<br>12 weeks (Follow-up phase): 12 weeks after starting the test (follow-up)<br>** Use the Samsung Galaxy Fit2 and Samsung Galaxy Fit applications (for Galaxy and iOS).<br>*** Smart bands will be optionally provided to those who wish to wear them, and data will be collected collectively only for those who complete the study in the 12th week. However, data on dropouts will not be collected.
안전성 평가변수<br>Safety endpoints	1) 이상반응 평가<br>이상반응 유무 및 중재 방법과의 관련 여부 평가<br>2) 활력징후와 임상검사실 검사<br>활력징후와 임상검사실 검사를 통한 안전성 평가<br>1) Evaluation of adverse events: Assessment of the presence or absence of adverse events and their relationship to the intervention method.<br>2) Vital signs and clinical laboratory tests: Vital signs and clinical laboratory tests are used to assess safety.
통계분석<br>Statistical analysis	1) 유효성 분석 방법<br>① 1차 유효성 평가변수<br>• 무작위 배정을 받은 시험대상자 중 적어도 한 번 이상 중재를 받고 시험 전과 중재 시술 이후 적어도 한 번 이상 MDS-UPDRS Part III 점수가 측정된 대상자의 MDS-UPDRS Part III 점수를 분석한다.<br>• MDS-UPDRS Part III 점수의 결측이 발생한 경우, 결측량과 기전에 대한 진단을 먼저 시행한 후 적합한 imputation 방법을 선정하여 Full Analysis Set (FAS) 분석을 시행한다.<br>• MDS-UPDRS Part III 점수의 변화에 대한 두 군의 차이를 independent t test를 통해 검증한다. 기저 값의 유의한 차이가 있는 경우는 이를 보정한 공분산 분석을 시행하고, 기타 기저 변수의 유의한 차이를 보정하기 위해서는 다중회귀분석을 시행한다.<br>• 각 시점별 MDS-UPDRS Part III 점수에 대한 반복 측정된 값을 대상으로 반복측정분산분석을 통해 시간과 치료간의 교호작용을 검정한다.<br>② 2차 유효성 평가변수<br>• MDS-UPDRS, TUG, PSQ, PDSS, IPAQ, GAITRite, fNIRS, 스마트밴드 (걸음수, 수면시간, 수면패턴 등) 등 검사는 공분산분석, 혹은 기저조사의 변수를 보정한 다중회귀분석을 사용하여 분석한다. 이때 시험 시작 전 점수가 측정된 경우는 이를 공변량으로 하며, 결측치에 대한 별도의 처리는 하지 않는 per-protocol 분석을 시행한다.<br>③ 장내미생물 (baseline)과 8주 후 미생물별 비율 변화량<br>• 정규성 검정을 시행하여 정규성을 따르는 경우 paired t-test를, 정

	규성을 따르지 않는 경우 Wilcoxon singed-rank test를 시행한다.<br>2) 경제성평가 분석<br>- 주 경제성 평가지표(Primary economic endpoint)는 Quality Adjusted Life Years(cost per QALY) gained, 이차평가지표는 cost per EQ-VAS이다.<br>- 일차 분석 기간은 12주로 시행, 이후의 기간에 대한 추정이 필요할 경우 비용과 효과를 회귀모형 등을 통하여 외삽(Extrapolation)하여 추정하거나 Decision modeling analysis 등을 시행한다.<br>- 임상시험과 관련하여 발생하는 치료비용은 치료 횟수와 단위비용을 결합하여 산출하며, 임상시험 기관 내에서 발생하는 치료비용은 맹검 해제 후 연구자가 전산 자료를 조사하여 기록한다. QALY 산출을 위한 삶의 질 추정은 EQ-5D로 도출된 삶의 질을 주 평가변수로 사용한다.(Area under the curve method)<br>- 분석관점은 사회적 관점이며, Baseline analysis에서는 연구에서 사용되는 모수들(parameter)의 대표값(평균 등)을 사용하여 시행하고, 민감도분석은 가능한 모든 추정모수들의 분포와 대표값들을 사용하여 확률적 민감도 분석(Probabilistic Sensitivity Analysis)을 시행한다.<br>3) 안전성 평가변수<br>- 이상반응의 빈도, 발현율, 각각의 목록, 상세한 발현시간, 심각한 정도 및 시험약물과의 인과관계 등을 제시하며, 필요한 경우 그래프 형태로 보고한다. 통계적 분석이 필요한 경우는 변수의 특성과 목적에 따라 paired t-test, Mcnemar test, ANOVA, independent t-test, chi-square test 혹은 Fisher's exact test 등을 실시한다.<br>1) Efficacy analysis method<br>① Primary efficacy endpoint<br>- Analyze the MDS-UPDRS Part III scores of randomly assigned subjects who received the intervention at least once and had their MDS-UPDRS Part III scores measured before and after the intervention.<br>- If an MDS-UPDRS Part III score is missing, the missing amount and mechanism are first determined, followed by an appropriate imputation method and Full Analysis Set (FAS) analysis.<br>- An independent t-test will confirm the difference in MDS-UPDRS Part III score change between the two groups. Covariance analysis will be used to correct for significant differences in baseline values, and multiple regression analysis will be used to correct for significant differences in other baseline variables.<br>- The interaction between time and treatment is investigated using repeated measures analysis of variance on MDS-UPDRS Part III scores at each time point.<br>② Secondary efficacy endpoint<br>- MDS-UPDRS, TUG, PSQ, PDSS, IPAQ, GAITRite, fNIRS, smart band (step count, sleep time, sleep pattern, and so on) results are subjected to covariance analysis or multiple regression with baseline survey variables corrected. If the score is measured before the start of the test, it is used as a covariate, and per-protocol analysis is performed without treating missing values separately.<br>③ Gut microbiome (baseline) and microorganism percentage change after 8 weeks

	- Perform a normality test. If normality is maintained, the paired t-test will be used. If normality is not followed, the Wilcoxon signed-rank test will be used.<br>2) Economic evaluation analysis<br>- The primary economic endpoint is Quality Adjusted Life Years (cost per QALY) gained, and the secondary endpoint is the cost per EQ-VAS.<br>- The primary analysis period is 12 weeks, and if a subsequent period estimate is required, the cost and effect will be extrapolated and estimated using a regression model, etc., or decision modeling analysis will be performed.<br>- Treatment costs associated with clinical trials will be computed by combining the number of treatments and unit costs, and treatment costs incurred within the clinical trial institution will be recorded by researchers examining computerized data after blinding has been removed. For calculating QALY, quality of life derived from EQ-5D will be used as the main evaluation variable (area under the curve method).<br>- The analytical viewpoint is a social point of view. Baseline analysis will be carried out using representative values (average, etc.) of the study's parameters. The distribution and representative values of all possible estimated parameters are used in the sensitivity analysis.<br>3) Safety evaluation variables<br>- The frequency, occurrence rate, list of each adverse event, detailed onset time, severity, causal relationship with the test drug, and other information will be presented and, if necessary, graphed. When statistical analysis is required, the paired t-test, Mcnemar test, ANOVA, independent t-test, chi-square test, or Fisher's exact test will be used, depending on the variable's characteristics and purpose.

임상시험 일정 (Clinical Trial Schedule)

기간period	스크리닝screening & 처치treatment						추적관찰 follow-up
주week	0~4				5~8		12
방문visit[1]	Screening	experimental group 1<br>control group 1	2~7W±1D	8W±1D<br>2W±7D	9~15W±1D	16W±1D<br>3W±7D	17W±7D<br>4W±7D
동의서 취득<br>Obtain informed consent form	●						
Hoehn & Yahr	●						
MMSE-K	●						
인구학적 조사<br>Demographic survey	●						
심전도 검사<br>Electrocardiography	●						
흉부 x-ray 검사<br>Chest x-ray examination	●						
홍채검사 1 & 2<br>Iris examination 1 & 2	●						
임상검사실 검사[2]<br>lab test	●					●	
병력 및 치료력 조사<br>Medical history and treatment history survey	●	●	●	●	●	●	●
활력징후 측정<br>Vital signs	●	●	◇	●	◇	●	●
선정/제외기준 확인<br>Check inclusion/exclusion criteria	●						
무작위 배정<br>Random assignment		●					
장내미생물검사<br>Gut microbiome		●				●	
신장/체중 측정[3][4]<br>Height/weight measurements		●		●		●	●
병용약물 확인<br>Check concomitant medications		●	●	●	●	●	●
이상반응 확인<br>Check for adverse events		●	●	●	●	●	●
MARS-PD[4]		◇	◇	◇	◇	◇	
MDS-UPDRS		●		●		●	●
IPAQ		●		●		●	●
PSQ		●		●		●	●
PDSS		●		●		●	●
TUG		●				●	●
GAITRite		●				●	●

**fNIRS**[5)]		●				●	●
**효용측정**[6)]<br>Utility measurement		●		●		●	●
**비용측정**[7)]<br>Cost measurement		●		●		●	●
**스마트밴드**[8)]<br>Smart Band		●					●
**순응도 확인**<br>Check compliance			●	●	●	●	●
**방문일정 교육**<br>Visit schedule information		●	◇	●	◇	●	

1) Screening은 방문1로부터 0-7일 이내에 행해져야 한다. 방문1은 Baseline 방문이다. (방문허용일: 시험군은 Baseline으로부터 주±1일/ 방문17에서는 Baseline으로부터 12주 ±3일, 대조군은 Baseline으로부터 각 방문마다 허용일 ±7을 둔다.)
   Screening must be performed within 0-7 days from Visit 1. Visit 1 is the baseline visit. (Visit Allowed Date: For the experimental group, ±1 day per week from the baseline, for visit 17, 12 weeks ±3 days from the baseline, for the control group, ±7 days per week are allowed from the baseline for each visit.)
2) 임상검사실 검사, 임신진단검사: 임상시험 대상자는 내원하여 다음의 항목을 검사한다. 방문1 기준 14일 이내의 검사결과가 있다면 적용 가능하며, 시험자의 판단에 따라 비정상적인 결과에 대한 재검사를 시행할 수 있다.
   Clinical laboratory test, pregnancy diagnostic test: Clinical test subjects visit the hospital and test the following items. It is applicable if there are test results within 14 days of visit 1, and re-testing can be performed for abnormal results at the investigator's discretion.
   - 혈액학적 검사: 헤모글로빈, 헤마토크릿, 적혈구수, 백혈구수, 혈소판수, HbA1c
     Hematological tests: hemoglobin, hematocrit, red blood cell count, white blood cell count, platelet count, HbA1c
   - 혈액화학적 검사: AST, ALT, γ-GTP, BUN, creatinine
     Blood chemical tests: AST, ALT, γ-GTP, BUN, creatinine
   - 소변검사: SG, pH, RBC(erythrocyte), WBC(leukocyte), Nitrite, Protein, Glucose, Ketone, Urobilinogen, Bilirubin
     Urine test: SG, pH, RBC (erythrocyte), WBC (leukocyte), Nitrite, Protein, Glucose, Ketone, Urobilinogen, Bilirubin
   - 임신진단검사: 폐경이 되지 않은 여성 대상자는 Urine HCG를 시행하며 결과는 음성이어야 한다. 단, Screening에서 menstruation에는 visit1에 시행하며, 방문16(대조군 방문3)에서 menstruation에는 방문17(대조군 방문4)에 시행한다.
     Pregnancy diagnostic test: Urine HCG is performed on female subjects who have not entered menopause, and the results must be negative. However, in screening, menstruation is conducted on visit 1, and menstruation on visit 16 (control group visit 3) is performed on visit 17 (control group visit 4).
3) 신장은 방문1에서만 측정하며, 체중은 방문1, 8, 16, 17에 측정하여 기록한다. 신장은 0.1cm, 체중은 0.1kg 단위까지 반올림하여 측정한다.
   Height is measured only at visit 1, and weight is measured and recorded at visits 1, 8, 16, and 17. Height is measured to the nearest 0.1 cm and weight is measured to the nearest 0.1 kg.
4) MARS-PD와 ◇로 표시한 항목은 모두 시험군에 한해 실시한다.
   MARS-PD and all items marked with ◇ are administered only to the experimental group.
5) fNIRS 검사 시에 보행검사(GAITRite)를 수행한다.
   During the fNIRS test, a gait test (GAITRite) is performed.
6) 효용측정은 EQ-5D, EQ-VAS를 사용하여 수행한다.
   Utility measurement is performed using EQ-5D and EQ-VAS.
7) 비용측정은 직접의료비, 직접비의료비, 간접비의 조사를 위하여 별도 개발된 비용 조사지를 사용하여 조사한다.
   Cost measurement is conducted using a separate cost survey form developed to investigate direct medical costs, non-direct medical costs, and indirect costs.
8) 스마트밴드는 착용을 원하는 대상자에게 선택적으로 제공하며, 연구 완료자에 한하여 12주차에 데이터를 일괄 수집한다. 단, 중도탈락자의 경우 데이터를 수집하지 않으며, 스마트밴드를 지급한 대상자에 한하여 방문시마다 밴드 착용여부를 확인한다.
   Smart bands are optionally provided to those who wish to wear them, and data is collected in batches in the 12th week only for those who complete the study. However, data will not be collected for those who drop out, and only those who have been provided with a smart band will be checked to see if they are wearing the band at each visit.

1. 임상시험의 명칭
Name of clinical trial

[국문] 파킨슨병에 대한 경근 활성 치료 시스템의 평가를 위한 단일기관, 평가자 맹검, 평행설계 무작위 대조 임상시험
[English] MARS-PD: Meridian Activation Remedy System for Parkinson's Disease — A Single-center, Rater-blinded, Parallel Randomized Controlled Trial

2. 임상시험 실시기관, 임상시험 책임자/담당자
Clinical trial implementation institution, clinical trial director/person in charge

2.1 주관기관
Host organization

대전대학교 대전한방병원. 대전광역시 서구 대덕대로 176번길 75
전화번호: 042-470-9131
Daejeon Korean Medicine Hospital of Daejeon University. 75, Daedeok-daero 176beon-gil, Seo-gu, Daejeon.
Phone: 042-470-9131

2.2 실시기관
Implementing organization

대전대학교 대전한방병원. 대전광역시 서구 대덕대로 176번길 75
전화번호: 042-470-9131
Daejeon Korean Medicine Hospital of Daejeon University. 75, Daedeok-daero 176beon-gil, Seo-gu, Daejeon.
Phone: 042-470-9131

2.3 임상시험 책임자
Clinical trial director (Principal Intestigator)

유 호 룡 대전대학교 한의학과 한방내과 교수
HoRyong Yoo, Professor of Internal Korean Medicine,
Daejeon University College of Korean Medicine

2.2.2 임상시험 공동 연구자
Clinical trial co-investigator

[별첨 1] 임상시험 담당자 명단 참조

[Appendix 1] See list of clinical trial personnel

2.2.3 연구의사
Research clinician
[별첨 1] 임상시험 담당자 명단 참조
[Appendix 1] See list of clinical trial personnel

2.2.4 통계담당자
Statistician
[별첨 1] 임상시험 담당자 명단 참조
[Appendix 1] See list of clinical trial personnel

2.2.5 임상연구 코디네이터
Clinical Research Coordinator
[별첨 1] 임상시험 담당자 명단 참조
[Appendix 1] See list of clinical trial personnel

3. 임상시험 의뢰자
Clinical trial client
유 호 룡 대전대학교 한의학과 한방내과 교수
대전대학교 대전한방병원. 대전광역시 서구 대덕대로 176번길 75
전화번호: 042-470-9131
HoRyong Yoo, Professor of Internal Korean Medicine
Daejeon Korean Medicine Hospital of Daejeon University. 75, Daedeok-daero 176beon-gil, Seo-gu, Daejeon.
Phone: 042-470-9131

4. 임상시험 모니터
Clinical trial monitoring

대전대학교 대전한방병원 임상시험센터 A-CRO
Daejeon Korean Medicine Hospital of Daejeon University Clinical Trial Center A-CRO

5. 임상시험의 목적 및 배경
Purpose and background of clinical trials

5.1 목적
Purpose

파킨슨병 환자의 안정 시 떨림, 서동, 근육 경직, 자세 반사 장애, 보행장애 등의 운동증상과 변비, 자율신경 장애, 우울 등의 비운동증상에 대한 경근 활성 재활 시스템(MARS-PD)의 안전성, 유효성 및 경제성을 평가한다.
The purpose of this study is to assess the clinical efficacy and cost-effectiveness of MARS-PD. Because the goal of this trial is to assess the add-on effect of MARS-PD, we will use usual care as the comparator. Our hypothesis is that complex therapy (MARS-PD) will alleviate motor and nonmotor symptoms, improve gait performance, and enhance neuroplasticity in Parkinson's disease (PD) patients while remaining safe and cost-effective.

5.2 배경
Background

5.2.1 기술적 측면
Technical aspects

파킨슨병은 특히 노년기 유병률이 높은 질환으로 세계적 인구 고령화에 따라 환자가 빠르게 증가하고 있다. 또한, 파킨슨병의 유병률은 나이에 의한 영향을 보정하더라도 여전히 증가하고 있으며, 사회인구학적 지수가 높은 선진국일수록 증가하는 경향성을 보인다. 따라서 전 세계적으로 정밀하고 체계적인 연구와 대책 마련이 필요하다.
Parkinson's disease has a high prevalence in the elderly, and the number of patients is rapidly increasing as the global population ages. Furthermore, even after adjusting for the effect of age, the prevalence of Parkinson's disease continues to rise, and it tends to rise in developed countries with higher sociodemographic indices. As a result, precise and systematic research and countermeasures are required globally.

파킨슨병 치료에서 가장 보편적인 일차적 선택은 약물치료이다. 도파민계 약물로는 레보도파(Levodopa) 및 도파민 작용제(Dopamine agonists), 모노아민산화효소-B(Monoamine oxidase B; MAO-B) 억제제, 그리고 카테콜-O-메틸전이효소(Catechol-O-methyltransferase; COMT) 억제제가 있으며, 비-도파민계 약물로는 항콜린성제제(Anticholinergics), NMDA 길항제(NMDA antagonist)가 있다. 파킨슨병에 대한 약물적 개입을 초기부터 시작하는 것과 최대한 미루는 것

에 대해 학자 간 견해가 분분하다. 약물치료 시 질병 진행을 어느 정도 늦출 수는 있으나, 약물의 사용 기간에 비례하여 약효가 감소하며, 결국 증상 조절이 불가한 상황으로 진행될 수 있어, 약물의 적절한 사용과 모니터링이 요구된다.
Drug therapy is the most commonly used first-line treatment for Parkinson's disease. Levodopa and dopamine agonists, monoamine oxidase B (MAO-B) inhibitors, and catechol-O-methyltransferase (COMT) inhibitors are all dopaminergic drugs. Anticholinergics and NMDA antagonists are examples of non-dopaminergic drugs. Scholars do not agree on whether to begin pharmacological intervention for Parkinson's disease as soon as possible or to wait as long as possible. Although drug treatment can slow the progression of the disease, the efficacy of the drug decreases in proportion to the length of drug use, and symptoms may eventually progress to the point where control is impossible, so appropriate drug use and monitoring are required.

2005~2006년 파킨슨병 환자를 대상으로 한 설문 연구에서 123명의 환자 중 94명(76%)이 파킨슨병 운동증상, 피로, 통증, 변비 등의 증상 개선을 목적으로 한의 치료와 같은 보완대체요법을 시행한 적이 있다고 보고된 바 있고, 이들 중에서 파킨슨병 이환 기간이 길고, 레보도파 복용량이 많을수록 보완대체요법을 이용하는 비율이 높은 것으로 나타난다. 특히, 파킨슨병 운동치료의 중요성이 점차 강조되고 있으며, 관련 연구도 늘어나는 추세이다. 파킨슨병의 균형, 자세 안정성, 보행, 낙상을 키워드로 PubMed에서 2009년부터 2018년 사이 검색되는 리뷰 논문이 167편, 임상시험 논문이 299편이다. 이 중 파킨슨병 환자의 균형장애, 보행장애, 근력 약화, 낙상 등에 대한 운동치료의 효과를 확인하고자 하는 연구들이 많았다.
In a survey of Parkinson's disease patients conducted between 2005 and 2006, 94 out of 123 patients (76%) reported having received Oriental medicine treatment and complementary and alternative therapy to improve motor symptoms, fatigue, pain, and constipation. Among the respondents, the longer the period of Parkinson's disease and the higher the dose of levodopa, the higher the rate of use of complementary and alternative therapy. Exercise treatment for Parkinson's disease, in particular, is becoming increasingly important, and related research is expanding. Between 2009 and 2018, the keywords balance, postural stability, gait, and falls in Parkinson's disease were searched in PubMed, yielding 167 review papers and 299 clinical trial papers. Among these were numerous studies aimed at confirming the effects of exercise therapy on Parkinson's disease patients' balance disorders, gait disorders, muscle weakness, and falls.

5.2.2 경제.산업적 측면
파킨슨병은 만성 진행성 경과를 나타내는 질환으로, 환자가 지속적인 관리와 치료를 받아야 하므로 사회경제적 부담이 상당하다. 건강보험심사평가원 2016년 진료비통계지표에 의하면 파킨슨병으로 의과에 입원한 환자는 약 2.7만 명으로 전체 질환 중 92위에 해당하였으나, 요양급여비용은 약 3,238억 원에 달하여, 1인당 의료비용이 매우 많이 드는 편이다. 한편, 같은 해 한의과에 입원한 환자는 0.4만 명으로 전체 질환 중 17위에 해당하였고, 요양급여비용은 약 26억 원에 달하였다. 입원환자 수 기준으로 파킨슨병은 한의 의료기관에 입원하는 주요 질병 중 하나이다.

Parkinson's disease is a chronic, progressive disease that requires ongoing management and treatment, resulting in a significant socioeconomic burden. According to the Health Insurance Review and Assessment Service's 2016 medical cost statistics, approximately 27,000 Parkinson's disease patients were hospitalized, ranking 92nd among all diseases. The cost of nursing care for Parkinson's disease, on the other hand, is approximately 323.8 billion won, implying that the medical costs per person are extremely high. Meanwhile, the number of patients hospitalized at the Korean Medicine Department in the same year was 0.4 million, ranking 17th among all diseases, and the cost of nursing care was approximately 2.6 billion won. Parkinson's disease is one of the most common diseases hospitalized in Korean Medicine medical institutions, based on the number of inpatients.

파킨슨병이 고령자에게 더 빈번하게 발생하는 신경퇴행성질환으로 약물 치료를 통해 모든 증상을 관리하기 어렵고 부작용 발생까지 고려하면 향후 파킨슨병 환자의 한의 치료에 대한 수요가 계속 증가하리라 예상된다.
Because Parkinson's disease is a neurodegenerative disease that primarily affects the elderly, it is difficult to manage all symptoms with drug treatment. Given the possibility of side effects, it is expected that the demand for Korean Medicine treatment for Parkinson's disease patients will increase in the future.

5.2.3 사회.문화적 측면
Social and cultural aspects

파킨슨병은 지속적인 관리와 치료가 필요한 만성 퇴행성 질환이다. 그럴 뿐만 아니라 여러 비운동성 증상이 발생하기 때문에 이에 대한 다양한 치료적 접근 방법이 필요하다.
Parkinson's disease is a chronic degenerative disease that necessitates ongoing monitoring and treatment. Furthermore, because different non-motor symptoms occur, different therapeutic approaches are required.

5.2.4 국내·외 기술개발현황
Domestic and international technology development status

파킨슨병으로 인한 사회경제적 부담은 전 세계적으로 빠르게 증가하는 추세이다. 미국 내 파킨슨병 환자는 100만 명에 달하고, 2015년 기준 미국 국립보건원은 약 1억 4천6백만 달러(한화 약 1,606억 원)를 파킨슨병 연구에 지원하고 있다. 수많은 파킨슨병 신약 개발 연구에도 불구하고, 여전히 1960년대 개발된 레보도파가 파킨슨병에 가장 효과적인 치료제이다. 그러나 장기 투여 시 여러 부작용이 발생해 이환 기간이 길어지면서 치료 한계를 경험하게 된다. 특히 파킨슨병의 병리기전 자체를 조절할 수 있는 치료가 부재한 상태로, 이에 치료제 개발에 대한 요구가 증가하고 있다.
The socioeconomic burden of Parkinson's disease is rapidly increasing around the world. In the United States, there are approximately 1 million Parkinson's disease patients, and the National Institutes of Health supports approximately $146 million (approximately 160.6 billion KRW) in Parkinson's disease research as of 2015. Despite numerous studies on the

development of new Parkinson's disease drugs, levodopa, developed in the 1960s, remains the most effective Parkinson's disease treatment. Long-term administration, on the other hand, causes a variety of side effects, prolongs the disease period, and limits treatment options. Because there is currently no treatment that can control the pathological mechanism of Parkinson's disease, there is an increasing demand for the development of a treatment.

5.2.5 현기술상태의 취약성(문제점)

Vulnerabilities (problems) of the current state of technology

현재 파킨슨 치료제로 널리 사용되고 있는 L-dopa와 같은 도파민계 약물은 파킨슨병의 운동 완서, 경직, 떨림과 같은 증상을 완화시키는 효과가 있지만, 복용 기간이 길어질수록 약효가 나타나는 기간은 단축되며 이상운동증(Levodopa Induced Dyskinesia)과 같은 문제가 심해진다. 또한, COMT 억제제, MAO-B 억제제, 도파민 효현제 등이 치료약으로 사용되고 있지만, 어지럼, 오심, 두통, 구토, 졸림, 이상행동 및 정신증, 환각, 혼돈, 저혈압 등의 부작용이 나타날 수 있다. 항콜린성 제제나 아만타딘 같은 비-도파민계 약물 역시 입 마름, 변비, 시야 흐림, 요 저류, 두통 및 오심, 혼돈, 이상행동 등이 발생할 수 있다. 뇌 심부 자극술은 뇌 기저부의 이상 부분에 전기자극을 주어 이상 신경을 조절하여 이상 운동증상을 호전시키는 치료방법이다. 드물지만 뇌 심부 자극술로 인한 사망, 뇌내출혈, 감염, lead misplacement 등 부작용이 발생할 수 있다.
Dopaminergic drugs, such as L-dopa, which are currently widely used as Parkinson's treatments, are effective in alleviating symptoms of Parkinson's disease such as bradykinesia, rigidity, and tremors. However, as the duration of treatment increases, the duration of effect shortens and problems such as Levodopa Induced Dyskinesia worsen. COMT inhibitors, MAO-B inhibitors, and dopamine agonists are also used as treatments, but they can cause dizziness, nausea, headache, vomiting, drowsiness, abnormal behavior, psychosis, hallucinations, confusion, and low blood pressure. Non-dopaminergic medications, such as anticholinergics or amantadine, can cause dry mouth, constipation, blurred vision, urinary retention, headache and nausea, confusion, and abnormal behavior. Deep brain stimulation is a treatment method that improves symptoms of abnormal motor symptoms by controlling abnormal nerves by providing electrical stimulation to the abnormal part of the deep brain. Deep brain stimulation can cause side effects such as death, intracerebral hemorrhage, infection, and lead misplacement in rare cases.

5.2.6 앞으로의 전망

Future prospects

최근 고령화가 급속도로 진행 중인 만큼 고령에서 빈발하는 파킨슨병과 같은 만성 퇴행성 질환의 치료방법에 관한 연구는 필수적이다. 본 연구에서 유효한 결과를 도출한다면 국민 보건 향상과 삶의 질 개선에 이바지할 수 있을 것으로 사료된다.
As the world's population ages, research into treatment methods for chronic degenerative diseases such as Parkinson's disease, which commonly affect the elderly, is critical. If this study yields valid results, it is hoped that it will contribute to bettering public health and

quality of life.

6. 대상질환과 증상
Target diseases and symptoms

파킨슨병의 운동증상 및 비운동증상
Motor and non-motor symptoms of Parkinson's disease

7. 중재방법
Intervention method

7.1 침치료 방법
Acupuncture treatment method

7.1.1 침치료 방법 및 설정의 합리성
Rationality of acupuncture treatment methods and settings

STRICTA 항목<br>STRICTA Item	항목기술<br>Item	본 연구에서 사용할 방법의 기술<br>Methods to be used in this study
1) Acupuncture rationale	1a) Style of acupuncture	정경침<br>12 meridian acupuncture
	1b) 치료법에 대한 근거와 만약 개별 치료를 하였다면 그에 대한 근거<br>Treatment rationale and, if individual treatment was used, evidence for it	경락경혈학 교과서, 파킨슨병 한의임상진료지침, 파킨슨병 침 치료에 대한 문헌 고찰을 통하여 가장 효과적일 것으로 예상되는 치료법 선정<br>The most effective treatment was chosen after a review of literature on meridian acupuncture textbooks, Korean Medicine clinical practice guidelines for Parkinson's disease, and articles on acupuncture treatment for Parkinson's disease.
	1c) 이론을 정당화할 수 있는 문헌적 근거<br>Documentary support for the theory	대한경락경혈학각론(상), (하), 파킨슨병 한의표준임상진료지침, 파킨슨병 침치료 관련 다수 논문<br>Introduction to Korean Meridian Acupuncture Points (volume 1), (volume 2), Standard Clinical Practice Guidelines for Korean Medicine for Parkinson's Disease, and numerous papers related to acupuncture treatment for Parkinson's Disease
2) Needling details	2a) 사용혈<br>Aupoints used	중충 외관 어제 양릉천 족삼리 태충 합곡 내관 곤륜 태계 후계<br>Hegu (LI4), Houxi (SI3), Waiguan (TE5), Neiguan (PC6), Zhongchong (PC9), Yuji (LU10), Zusanli (ST36), Yanglingquan (GB34), Taichong (LR3),

		Kunlun (BL60), and Taixi (KI3)
	2b) 사용한 침의 개수<br>Nmber of needles	총 22개<br>Total: 22
	2c) 자침 깊이(조직, mm나 촌)<br>Depth of insertion, based on a specified unit of measurement, or on a particular tissue level	수직 자입으로 1~1.5 ㎜<br>About 1~1.5 ㎜, intradermally
	2d) 유발된 반응<br>Response sought	없음<br>None
	2e) 침 자극의 형태(수기침이나 전침)<br>Needle stimulation	수기침<br>manual, intradermal acupuncture
	2f) 유침 시간<br>needle retention time	30분<br>30 minutes
	2g) 침의 형태(두께, 길이, 생산회사 및 재질)<br>Needle type	0.18mmX1.3mmX1.5mm, 동방메디컬, stainless steel<br>0.18x1.3 stainless steel intradermal needle (Dongbang Medical Co., South Korea)
3) Treatment regimen	3a) 치료 횟수<br>Number of treatment sessions	16회<br>Total: 16
	3b) 치료 빈도<br>Frequency and duration of treatment sessions	8주간 주당 2회 (총 16회)<br>2 times per week, a total of 8 weeks
4) Co-intervention	4a) 다른 처치 (뜸, 부항, 약물, 운동, 생활 습관에 대한 조언 등)<br>Details of other interventions administered to the acupuncture group	스마트밴드를 활용하여 생활습관 관리를 하며, 환자가 기존 복용하던 병용약물 및 병용치료(한의 치료를 제외한 usual care)는 허용하되 본원에서 추가 중재를 시행하지는 않는다. 또한, 시험 기간 동안 환자가 개인적으로 수행하는 운동은 허용하나, 의사의 처방에 따른 파킨슨병에 대한 도수치료, 운동치료, 재활치료는 허용하지 않는다.<br>Smart bands will be provided to participants, and lifestyle management using an application can be provided. Existing concomitant medications and treatments (except for Korean Medicine treatment) will be permitted, but no additional intervention will be performed at our hospital. Furthermore, patients will be permitted to exercise individually during the test period, but manual therapy, exercise therapy, and Parkinson's disease rehabilitation treatment prescribed by a doctor will not be permitted.
5) Practitioner	5a) 관련분야 훈련 기간	1년 이상의 해당 분야 임상경력을 가진 한의사가

background	Training period in related field	시행<br>Performed by a Korean Medicine Doctor with more than 1 year of clinical experience in the relevant field
	5b) 임상 경험 기간<br>Length of clinical experience	1년 이상<br>more than 1 year
	5c) 특정 질병상황에 있어서 전문적 기술<br>Professional skills in specific disease situations	한방내과 전문의, 석사, 박사 또는 해당 과정을 연수 중에 있는 한의사로 1년 이상의 해당 분야 임상경험이 있는 자가 시행<br>Internal Korean medicine specialist, master's or doctorate degree holder, or doctor of Korean medicine with at least one year of clinical experience in the relevant field performs the procedure.
6) Control intervention(s)	6a) 대조군에서 기대하는 효과와 연구에 있어서 선택한 대조군이 적당한지 여부 및 적당한 경우 참가자의 맹검 여부<br>6a) Justification for the control or comparator in the context of the research question, including sources to back up this choice	MARS-PD 치료의 효과를 확인하기 위하여 시험군에만 침 시술 방법을 적용함. 스마트밴드를 활용한 생활습관 관리 및 활동량 모니터링은 두 군 모두 시행함. 모든 환자에 대하여 환자가 기존 복용하던 병용약물 및 병용치료(한의 치료를 제외한 usual care)는 허용하되 본원에서 추가 중재를 시행하지는 않는다. 또한, 시험 기간 동안 환자가 개인적으로 수행하는 운동은 허용하나, 의사의 처방에 따른 파킨슨병에 대한 도수치료, 운동치료, 재활치료는 허용하지 않는다.<br>The acupuncture method was only used on the experimental group to confirm the efficacy of MARS-PD treatment. Both groups used smart bands for lifestyle management and activity monitoring. Concomitant medications and concomitant treatments (usual care excluding Korean Medicine treatment) are permitted for all patients, but no additional intervention is performed at our hospital. Furthermore, patients will be allowed to exercise individually during the test period, but manual therapy, exercise therapy, and Parkinson's disease rehabilitation treatment prescribed by a doctor will not be permitted.

7.2 파킨슨병에 대한 경근 활성 재활 시스템(MARS-PD) Meridian Activation remedy system for Parkinson's Disease

(MARS-PD)

7.2.1 파킨슨병에 대한 경근 활성 재활 시스템 수행방법
Meridian Activation remedy system for Parkinson's Disease (MARS-PD) method

MARS-PD: 치료군에 대해 실시한다.
MARS-PD: will be conducted on the experimental group.

1년 이상의 임상경력을 가진 한의사가 시행한다.
It will be performed by a Korean Medicine Doctor with more than one year of clinical experience.

- 처치 부위 : 중충 외관 어제 양릉천 족삼리 태충 합곡 내관 곤륜 태계 후계
- Acupoints: Hegu (LI4), Houxi (SI3), Waiguan (TE5), Neiguan (PC6), Zhongchong (PC9), Yuji (LU10), Zusanli (ST36), Yanglingquan (GB34), Taichong (LR3), Kunlun (BL60), and Taixi (KI3)

- 시술 방법 : 양측 중충 외관 어제 양릉천 족삼리 태충 합곡 내관 곤륜 태계 후계 각각 1개(총 22개)의 0.18×1.3×1.5 mm 피내침(동방 침구 제작소)을 이용하여 각 혈위에 자침한다.
- Acupuncture intervention method: bilateral Hegu (LI4), Houxi (SI3), Waiguan (TE5), Neiguan (PC6), Zhongchong (PC9), Yuji (LU10), Zusanli (ST36), Yanglingquan (GB34), Taichong (LR3), Kunlun (BL60), and Taixi (KI3) will be stimulated using 0.18x1.3 stainless steel intradermal needle (Dongbang Medical Co., South Korea).
- 운동 방법 : 준비 스트레칭, 상지운동, 하지운동, 전신운동 등으로 구성된 경근 활성 운동을 서서 또는 의자에 앉아서 비트 소리에 맞춰 천천히(약 30bpm), 빠르게(약 120bpm) 번갈아 수행한다.
- Exercise method: While standing or sitting on a chair, perform MARS exercises consisting of preparatory stretching, upper extremity exercises, lower extremity exercises, and full body exercises alternately slowly (approximately 30 bpm) and quickly (approximately 120 bpm).

[예시]
[example]
준비 스트레칭
Preparation Stretching

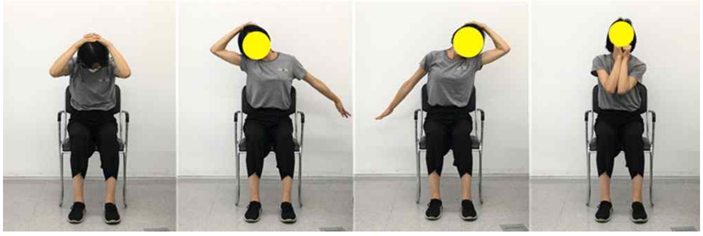

상지운동
Upper limb exercise

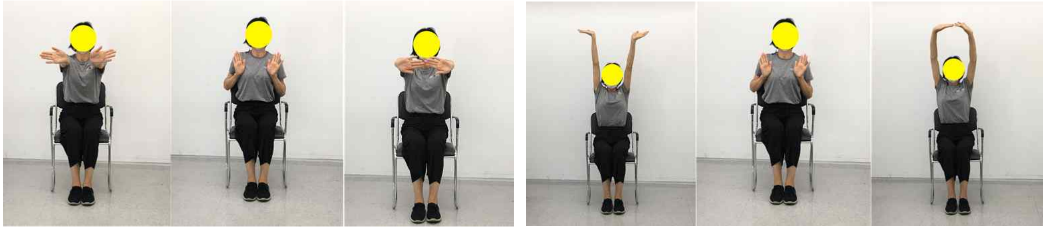

하지운동
Lower limb exercise

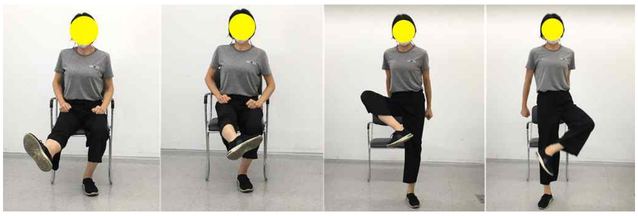

전신운동
Full body exercise

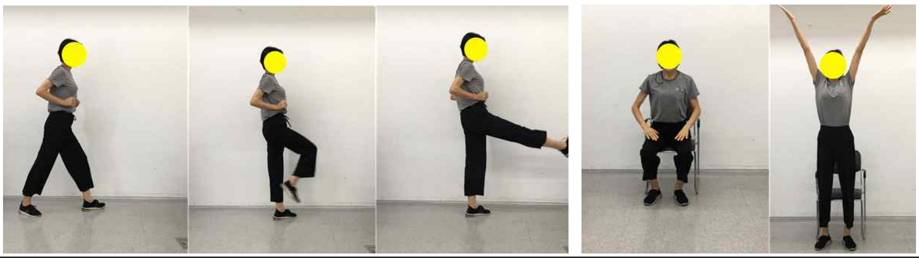

- 치료 횟수 : 주당 2회, 8주간 총 16회
  Number of treatments: 2 times per week, total 16 times over 8 weeks

- 참고 문헌: MARS-PD의 운동 동작을 구성하기 위해 아래와 같은 문헌들을 참고하였다. 조사 결과에 따르면 파킨슨병 환자들에게 고강도 훈련이 가능한 것으로 보이며, 특히 저항 운동과 유산소 운동을 결합한 형태의 운동이 효과적인 것으로 보였다. 본 연구에서는 태극권에서 주로 사용하는 동작들을 참고하여 이를 바탕으로 하되, 낙상 방지를 위해 의자를 적절히 활용하여 환자의 운동 능력에 따라 간편하면서도 효과적으로 저항 운동 및 유산소 운동을 할 수 있도록 동작을 구성하였다.
  References: The following literature was used to construct the MARS-PD exercise movements. According to the survey results, high-intensity training appears to be feasible for Parkinson's disease patients, and exercise that combines resistance and aerobic exercise appears to be especially effective. The movements primarily used in Tai Chi were referenced and based on in this study, but the movements were structured in such a way that resistance and aerobic exercise could be performed simply and effectively according to the patient's motor ability by appropriately utilizing a chair to prevent falls.

- 파킨슨병 환자를 대상으로 수행된 저항 훈련(resistance training, RT), 지구력 훈련(endurance training, ET), 그리고 기타 격렬한 훈련 방법(other intensive training modalities, OITM)에 대한 무작위 대조군 연구들을 조사하였다. 파킨슨병 환자들을 대상으로 고강도 운동(intensive

exercise)이 가능하며, 안전하다는 문헌들이 많았다. 저항 훈련, 지구력 훈련, 그리고 기타 훈련들은 모두 환자들의 균형 감각, 보행 능력, 그리고 파킨슨 환자들의 운동증상을 평가하는 UPDRS-III (Unified Parkinson's Disease Rating Scale Part III), 그리고 삶의 질을 개선하는 데 긍정적인 영향을 미칠 수 있다는 결과가 나타났으나, 연구에 따라 편차가 존재했다. 그 중 특히 저항 훈련을 시행한 연구에서 결과적으로 근력이 강화되는 등 효과가 가장 좋게 나타났다. 한편, 지구력 훈련을 시행한 연구에서는 참여한 환자들의 심폐기능이 다소 좋아진 것으로 나타났다. 운동 치료 후 환자가 악화되었다는 결과가 보고된 바는 없었다.
The study included randomized controlled trials of resistance training (RT), endurance training (ET), and other intensive training modalities (OITM) in Parkinson's disease patients. A large body of research shows that intense exercise is both possible and safe for Parkinson's disease patients. Resistance training, endurance training, and other exercises improved patients' balance, walking ability, and the Unified Parkinson's Disease Rating Scale Part III (UPDRS-III), which assesses motor symptoms in Parkinson's patients. The findings showed that different exercises can improve patients' quality of life, but the results varied depending on the study. Resistance training studies, in particular, produced the best results, including muscle strengthening. Meanwhile, studies on endurance training found that participants' cardiopulmonary function improved slightly. There have been no reports of patients deteriorating as a result of exercise therapy.

- 파킨슨병 환자를 대상으로 수행한 유산소 운동(aerobic exercise)에 대한 무작위 대조군 연구들을 조사하였다. 연구에 포함된 문헌들에서 중재로 사용하였던 유산소 운동의 종류로는 러닝머신(treadmill), 태극권, 보행 훈련, 춤 등이 있었다. 조사 결과, 유산소 운동은 UPDRS-III 점수를 개선하는 것으로 나타났으나, UPDRS-I, II, IV, 그리고 전체 UPDRS 점수에는 영향을 미치지 않는 것으로 나타났다. 절반 가량의 연구에서 유산소 운동이 환자들의 균형을 개선하는 효과가 있다고 보고하였으나 전체를 종합해서 평가했을 때는 이러한 개선 효과가 통계적으로 유의미하지는 않았다. 보행의 경우 유산소 운동의 효과가 아주 확실하게 나타났다. 일부 연구에서는 유산소 운동이 환자 삶의 질을 개선하였다고 보고하였으나, 다른 연구에서는 유산소 운동이 다른 중재에 비해 삶의 질 개선 효과가 차이가 날 정도로 크지는 않았다고 보고하였다.
  The researchers looked at randomized controlled trials of aerobic exercise on Parkinson's disease patients. Aerobic exercise interventions used in the study's literature included treadmill, tai chi, walking training, and dance. Aerobic exercise improved UPDRS-III scores but had no effect on UPDRS-I, II, IV, or overall UPDRS scores, according to the findings. Although approximately half of the studies reported that aerobic exercise was effective in improving patients' balance, this improvement effect was not statistically significant when evaluated overall. Walking clearly demonstrated the effect of aerobic exercise. Some studies found that aerobic exercise improved patients' quality of life, while others found that the effect of aerobic exercise on improving quality of life compared to other interventions was insignificant.

- 파킨슨병 환자를 대상으로 한 전반적인 운동 치료에 대한 무작위 대조군 연구들을 대상으로 한 체계적 문헌 고찰에 포함된 문헌들에서 중재로 사용하였던 운동의 종류로는 기공, 스트레칭, 유

산소 운동, 근력 운동, 균형 운동, 러닝머신, 보행 훈련, 물리치료 등이 있었다. 조사 결과, 운동치료는 신체 기능, 건강 관련 삶의 질, 근력, 균형, 보행 속도 등에 긍정적인 영향을 미치는 것으로 확인되었다. 하지만 운동이 환자의 낙상이나 우울감에 대하여 개선 효과가 있는지는 불분명하였다.
This study looks into randomized controlled trials of overall exercise therapy for Parkinson's disease patients. Qigong, stretching, aerobic exercise, strength exercise, balance exercise, treadmill, gait training, and physical therapy were all used as interventions in the study's literature. Exercise therapy was found to improve physical function, health-related quality of life, muscle strength, balance, and walking speed as a result of the study. It was unclear, however, whether exercise improved patients' falls or depression.

7.2.2 MARS-PD 치료의 고려사항
MARS-PD treatment considerations

피내침 시술을 먼저 한 뒤에 운동치료를 시행한다.
Intradermal acupuncture treatment will be performed first, followed by exercise therapy.

8. 임상시험 기간

본 임상시험의 기간은 본 임상시험계획서에 대한 기관생명윤리위원회(IRB) 승인일로부터 39개월로 한다. 단, 피험자 등록 속도에 따라 연장될 수 있다.
This clinical trial will last 39 months from the date the protocol was approved by the institutional review board (IRB). It may, however, be extended depending on the rate of subject registration.

9. 대상자 선정기준, 제외기준, 목표한 대상자 수 및 그 근거, 배정방법
Subject inclusion and exclusion criteria, target number of subjects and their basis, allocation method

9.1 선정기준
Inclusion criteria

1) 만 45세 이상 75세 이하의 남녀
2) 영국 파킨슨병학회 뇌은행 진단 기준(UK Parkinson’s Diseases Society Brain Bank Criteria)에 따라 파킨슨병을 진단받은 자
3) 혼-야의 분류법(Hoehn and Yahr Scale) 중 1~3단계에 해당하는 자
4) 연구대상자 본인이 자의로 임상연구에 참여를 결정하고 동의서에 서명한 자

1) 45 to 75 years of age
2) Patients with PD, diagnosed according to the UK Parkinson’s Diseases Society Brain Bank Criteria
3) Hoehn and Yahr scale stage I to III

4) Patients who have voluntarily decided to participate in the clinical study and signed the informed consent form

9.2 제외기준

1) 심각하게 불안정한 상태인 경우 (예) AST 또는 ALT 혈중치가 연구기관 정상 상한치의 3배 이상인 경우, 심부전증, 호흡부전증 등)
2) 연구 기간 내에 심부 뇌자극술을 계획 중인 환자
3) 임산부 또는 수 유부
4) MMSE-K(Mini-Mental State Exam) 점수가 18점 이하인 경우
5) 최근 4주 이내 의사 처방에 따른 항파킨슨약물(예: L-dopa, COMT inhibitor, Dopamine agonist, MAO-B inhibitor 등)의 복용량 변화가 있는 경우
6) 최근 4주 이내 의사의 처방에 따라 파킨슨병에 대한 도수치료, 운동치료, 재활치료를 받고 있거나, 또는 연구 기간 내에 해당 치료를 계획 중인 환자
7) 연구자의 판단에 따라 본 임상연구 참가가 적합하지 않은 경우

1) Clinically unstable patients (e.g., elevated aspartate transaminase(AST) or alanine aminotransferase(ALT) more than three-fold the upper limit of normal in the research institute's laboratory, heart failure, respiratory failure, etc.)
2) Patients who are planning to undergo deep brain stimulation within the study period
3) Pregnant or lactating women
4) Patients with MMSE-K(Mini-Mental State Exam) score of 18 or less
5) If there has been a change in the dosage of antiparkinsonian drugs (e.g., L-dopa, COMT inhibitor, Dopamine agonist, MAO-B inhibitor, etc.) according to a doctor's prescription within the last 4 weeks prior to enrollment
6) Patients who are receiving manual therapy, exercise therapy, or rehabilitation therapy for Parkinson's disease according to a doctor's prescription within the last 4 weeks prior to enrollment, or are planning to receive such therapy within the study period
7) Patients who are not suitable for participation in this clinical study according to the judgment of the researcher

9.3 목표한 대상자 수 및 그 근거
Targeted number of subjects and rationale

9.3.1 무작위배정 시험대상자 수
Number of randomly assigned test subjects

총 88명(시험군 44명, 대조군 44명)

A total of 88 people (44 experimental group, 44 control group)

	시험군<br>Experimental group	대조군<br>Control group	총 대상자수<br>Total number of subjects
유효성 평가 대상자 수<br>The number of subjects tested for efficacy	n = 36	n = 36	n = 72
탈락률, 순응도를<br>고려한 대상자수<br>(Drop-out: 20%)<br>Number of subjects taken into account in terms of dropout rate and compliance<br>(Drop-out: 20%)	n = 44	n = 44	n = 88

9.3.2 산출근거
Basis for calculation

1) 본 임상시험의 가설은 다음과 같다.
- H0(귀무가설): μt = μc
- H1(대립가설): μt ≠ μc
- μt : 치료군(운동+침치료)의 베이스라인 대비 8주 시점의 UDPRS Part III 점수 평균 변화량
- μc : 대조군의 베이스라인 대비 8주 시점의 UDPRS Part III 점수 평균 변화량
- 평가변수의 통계적 가설검정: 양측 검정 유의수준(α): 0.05 제2종 오류(β): 0.1, 검정력(1-β): 90%
- 시험군과 대조군의 비율은 1:1로 동일하게 한다.

2) 본 연구와 연구 디자인, 처치의 기간, 방법 및 횟수, 평가변수 등이 가장 유사한 선행연구 (Hackney & Earhart, 2008)를 참고하여 계산하였다.
- 해당 논문에서는 운동치료군 17명, 대조군 16명으로 총 33명 대상자를 모집하였다. 처치 기간은 10-13주였으며, 총 처치 횟수는 20회였다. 1차 유효성 평가변수는 MDS-UPDRS Part III를 사용하였다.
- 치료군과 대조군의 MDS-UPDRS Part III의 변화량에 대한 평균(표준편차)는 각각 –1.5(6.6)과 4.3(5.6)로 나타났다.
- 대조군 대비 치료군(운동)의 효과 차이 (Mean Difference) 는5.8이고, 합동표준편차 (Pooled SD)는 6.1로 나타났다.

3) 효과 차이는 5, 표준편차는 6.5로 보수적인 값을 설정하여 대조군 대비 치료군(운동+침치료)의 효과를 확인하기 위한 시험대상자 수를 산출하는 경우 결과는 아래와 같이 군당 약 36명으로 나타난다. 중도탈락률 20%를 고려하면 군당 44명씩 총 88명의 임상시험 대상자가 필요한 것으로 나타난다.

$$\left\{\frac{2\left(z_{1-\frac{\alpha}{2}}+z_{\beta}\right)^{2}*\sigma^{2}}{\left|\mu_{T}-\mu_{c}\right|}\right\}=\left\{\frac{2(1.96+1.28)^{2}*6.5^{2}}{5^{2}}\right\}=35.515\approx 36$$

4) 필요한 대상자 수는 각 군당 약 36명이고, 중도탈락률 20% 고려하면 $\frac{35.515}{0.8}\simeq 44명/군$ 으로 나타난

다. 즉, 군당 44명, 총 88명의 대상자를 등록한다.

* Hackney, Madeleine E., and Gammon M. Earhart. "Tai Chi improves balance and mobility in people with Parkinson disease." Gait & posture 28.3 (2008): 456-460.

Calculation basis:

1) The hypothesis of this clinical trial is as follows.
   - H0 (null hypothesis): μt = μc; H1 (Alternative hypothesis): μt ≠ μc
   - μt: Average change in UDPRS Part III score at 8 weeks compared to the baseline of the treatment group (exercise + acupuncture & usual care)
   - μc: Average change in UDPRS Part III score at 8 weeks compared to the baseline of the control group (usual care only)
   - Statistical hypothesis test of evaluation variables: two-tailed test
   - Significance level (α): 0.05
   - Type 2 error (β): 0.1, power (1-β): 90%
   - The ratio of experimental group and control group is 1:1.
2) The calculation was based on a previous study (Hackney & Earhart, 2008) that was most similar to this one in terms of research design, treatment period, method and number of treatments, and evaluation variables.
   A total of 33 subjects were recruited for this study: 17 for the exercise therapy group and 16 for the control group. The treatment period lasted 10-13 weeks, with a total of 20 treatments. MDS-UPDRS Part III was the primary efficacy endpoint. The treatment and control groups' average (standard deviation) changes in MDS-UPDRS Part III were -1.5 (6.6) and 4.3 (5.6), respectively, and the mean difference in the effect of the treatment group (exercise) compared to the control group was 5.8. Furthermore, the pooled standard deviation (Pooled SD) was discovered to be 6.1.
3) When the number of test subjects required to confirm the effect of the treatment group (exercise + acupuncture) compared to the control group is calculated using a conservative value of 5 for the effect difference and 6.5 for the standard deviation, the results show that approximately 36 people are required per group, as shown below. Given a 20% dropout rate, a total of 88 clinical trial subjects, 44 per group, are required.
   Calculation formula:
   $$\left\{\frac{2\left(z_{1-\frac{\alpha}{2}}+z_{\beta}\right)^{2}*\sigma^{2}}{\left|\mu_{T}-\mu_{c}\right|}\right\}=\left\{\frac{2(1.96+1.28)^{2}*6.5^{2}}{5^{2}}\right\}=35.515\approx 36$$
   Considering a dropout rate of 20%: 35.515/0.8=44.39375, approximately 44 people, total 88 people.
4) Reference paper: Hackney, Madeleine E., and Gammon M. Earhart. "Tai Chi improves balance and mobility in people with Parkinson disease." Gait & posture 28.3 (2008): 456-460.

9.4 무작위배정 방법, 시험대상자 식별 방법
Randomization method, test subject identification method

임상시험의 수행 및 평가에 관여하지 않는 무작위배정 담당자(또는 통계 담당자)가 구체적 계획 및 재현 가능한 방법으로 무작위배정 목록을 생성하고 무작위 배정표는 봉인하여 봉인해제 여부를 확인할 수 방식으로 보관하며 연구책임자가 별도로 관리한다.

A randomizer (i.e., a statistician) who is not involved in the conduct or evaluation of the clinical trial will create the randomization list using a specific plan and reproducible method. The randomization table will be managed separately by the principal investigator

and will be sealed and stored in a way that can be checked for unsealing.

무작위배정 담당자는 통계프로그램 SAS® Version 9.4(SAS institute. Inc., Cary, NC)를 사용하여 각 개체가 뽑힐 가능성이 동일한 상태에서 무작위로 각 군에 배정한다. 블록 무작위배정 방법(block randomization)을 이용한다. 시험군과 대조군의 무작위 배정은 1:1 비율로 실시한다.
The person in charge of randomization will employ the statistical program SAS® Version 9.4 (SAS Institute, Inc., Cary, NC) to assign each subject to each group at random with the same probability of selection. Block randomization will be used. The experimental and control groups will be assigned at random in a 1:1 ratio.

시험담당자는 대상자 앞에서 순서대로 무작위배정 봉투를 개봉하여 대상자를 배정하고 개봉한 봉투에는 개봉한 날짜와 시험담당자의 서명을 기재하여 별도 보관한다.
The test staff will assign subjects by opening randomization envelopes in front of the subjects in order. The opened envelopes will be kept separately, with the date of opening and the signature of the test staff written on them.

임상시험 참여에 서면으로 동의하고, 스크리닝 검사를 시행하는 시험대상자에게 다음의 스크리닝 번호를 부여한다.
Subjects who agree to participate in clinical trials and undergo screening tests in writing will be assigned the screening number listed below.

DJ-S-001 [DJ: 대전대학교 대전한방병원, S: Screening의 첫 글자, 001: 일련번호(001, 002~)]
DJ-S-001 [DJ: Daejeon Korean Medicine Hospital of Daejeon University, S: Screening, 001: Serial number(001, 002~)]

임상시험 참여에 동의하고, 시험대상자 적합성 평가를 통하여 선정 및 제외기준에 적합한 자에 한하여 시험대상자 식별코드를 부여한다.
Only those who agree to participate in the clinical trial and meet the inclusion and exclusion criteria through a test subject suitability assessment will be assigned a test subject identification code.

DJ-E-001 [DJ: 대전대학교 대전한방병원, E: Enrollment의 첫 글자, 001: 일련번호(001, 002~)]
DJ-E-001 [DJ: Daejeon Korean Medicine Hospital of Daejeon University, E: Enrollment, 001: Serial number(001, 002~)]

10. 임상시험 방법
Clinical trial method

10-1 임상시험 디자인

Clinical trial design

본 임상시험은 무작위배정, 평가자맹검, 평행설계로 진행한다.
This clinical trial is conducted with randomized, rator-blinded, and parallel design.

- 시험군 MARS-PD 치료
  experimental group: MARS-PD intervention
  - 주 2회, 총 16회 방문하여 MARS-PD 실시, 통상적 치료 및 생활 습관에 대한 조언
    MARS-PD conducted at 2 visits a week, for a total of 16 visits, Usual care, lifestyle advice
- 대조군
  control group
  - 통상적 치료(usual care) 및 생활 습관에 대한 조언
    Usual care, lifestyle advice

10-2 임상시험 과정 요약

Summary of clinical trial process

본 시험에서는 파킨슨 환자를 대상으로 파킨슨병에 대한 경근 활성 재활 시스템(MARS-PD)의 안전성, 유효성 및 경제성을 확인하고자 한다.
The purpose of this study is to confirm the safety, efficacy, and economic feasibility of the light muscle activation remedy system for Parkinson's disease (MARS-PD) in Parkinson's patients.

본 시험에 참여를 신청하고 동의서에 자필서명한 지원자 중에서 홍채검사 1, 홍채검사 2를 실시하고, 인구학적 조사, 병력 조사, 활력징후(혈압, 맥박, 체온), 흉부 X-ray (chest-PA) 검사, 심전도 검사, 임상검사실 검사 및 Hoehn & Yahr scale, MMSE-K를 통하여 선정 및 제외기준에 부합하는 대상자를 선정한다.
Applicants who applied for and signed the consent form to participate in this test are eligible. Iris examinations 1 and 2 will be performed. There is a demographic survey, a medical history survey, vital signs (blood pressure, pulse rate, and body temperature), a chest X-ray (chest-PA) examination, an electrocardiogram examination, clinical laboratory tests, the Hoehn & Yahr scale, and the MMSE-K. Subjects who meet the selection and exclusion criteria should be chosen.

대상자를 시험군(MARS-PD 치료), 대조군(무처치)로 무작위 배정한 후 MARS-PD 치료는 8주간 주 2회, 총 16회 실시한다. 시험 기간 8주와 추적관찰 12주 동안 MDS-UPDRS, IPAQ, 파킨슨 자가 검사지, PDSS, 비용측정 및 효용측정(EQ-5D, EQ-VAS) 설문은 4회(0주, 4주, 8주, 12주) 실시하고, TUG, GAITRite, fNIRS는 3회(0주, 8주, 12주) 실시한다. 장내미생물 검사는 2회(0주, 8주) 실시한다.
MARS-PD treatment will be administered twice a week for 8 weeks, a total of 16 times, after randomly assigning subjects to the experimental group (MARS-PD treatment) and the

control group (no treatment). During the 8-week test period and 12 weeks of follow-up, the following tests will be performed: the MDS-UPDRS, IPAQ, Parkinson's self-test questionnaire, PDSS, cost measurement, and utility measurement (EQ-5D, EQ-VAS) will be administered four times (weeks 0, 4, 8, and 12). TUG, GAITRite, and fNIRS will be administered three times (weeks 0, 8, and 12). Gut microbiome tests will be performed twice a week (week 0 and week 8).

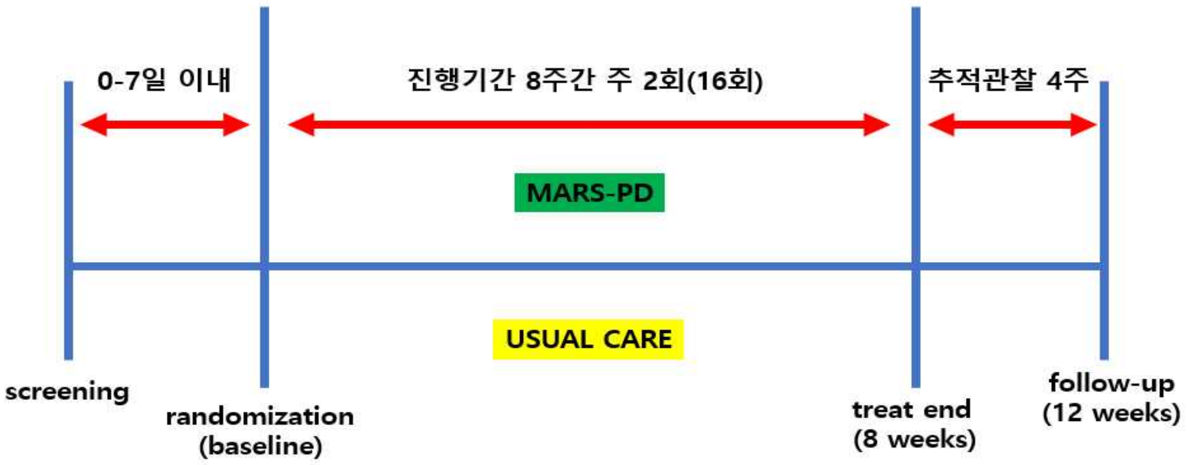

10-3 눈가림의 유지를 위한 고려
Considerations for maintaining blindding

10-3-1 시험대상자의 눈가림 유지
Maintain blinding of test subjects

본 임상시험의 중재 방법은 시험대상자의 눈가림을 유지할 수 없다.
The intervention method of this clinical trial cannot maintain blinding of subjects.

10-3-2 연구자 그룹의 눈가림 유지
Maintaining blinding of the researcher group

1) 임상시험 책임자 및 임상시험 담당자
Clinical trial director (principal investigator) and clinical trial staff

임상시험 담당자를 중재 담당자와 평가 담당자로 분리한다.
Divide clinical trial staff into intervention staff and evaluation staff.

중재 담당자는 시험대상자에 대한 중재 및 상담을 수행하고, 증례기록지를 작성한다. EMR로 인한 눈가림 해제 실수를 범하지 않기 위해 EMR 및 오더는 모두 평가자가 아닌 중재자의 이름으로 기록한다. CRF로 인한 눈가림 해제 실수를 범하지 않기 위해 CRF 서명은 중재자 또는 임상시험 책임자가 시행한다. 중재 담당자의 눈가림은 유지할 수 없다.
The intervention staff will mediate and counsel test subjects, and complete case report forms

(CRFs). EMRs and orders will all be recorded under the name of the intervention staff, not the evaluation staff, to avoid mistakes in unblinding caused by EMRs. The CRF signature will be performed by intervention staff or the clinical trial director to avoid errors in unblinding caused by CRF. The blinding of intervention staff cannot be sustained.

평가 담당자는 연구대상자의 적합성 판단, 동의서 획득, 시험대상자의 일련번호 부여 및 평가를 수행한다. 평가 담당자는 시험대상자가 어떤 종류의 치료를 받는지 모르도록 한다.
The evaluation staff will select research subjects, obtain consent forms, assign serial numbers to test subjects, and conduct evaluations. Assessors should not be aware of the subject's group.

2) 임상시험 코디네이터
Clinical Trial Coordinator

시험대상자별 일정 관리 등 임상시험 기초업무를 수행한다.
증례기록지를 작성한다.
코디네이터의 눈가림은 유지할 수 없다.
Perform basic clinical trial tasks such as schedule management for each test subject.
Complete the case report.
The coordinator's blindness cannot be maintained.

10.4 병용약물 투약 기준
Concomitant drug dosing standards

10.4.1 병용가능 약물
Possible concomitant drugs

임상시험 시작 전 4주 동안 의사 처방에 따른 항파킨슨 약물(예: L-dopa, COMT inhibitor, Dopamine agonist, MAO-B inhibitor 등)의 복용이 일정한 경우는 허용한다. 시험 기간 동안 의사 처방에 따른 약물 복용량은 일정하게 유지되어야 한다. 기타 질환의 일과성 치료를 목적으로 사용되는 약물은 연구의사와 상의를 통하여 병용 투여하기로 한다. 모든 병용약물 투여 시(타 질환 또는 이상반응 발현 시 치료약물 포함) 그 약물에 대한 정보(제품명, 투여목적, 투여용량, 투여기간 등)를 증례기록지에 상세히 기록한다.
Regular anti-Parkinson drugs (e.g., L-dopa, COMT inhibitor, Dopamine agonist, MAO-B inhibitor, etc.) prescribed by a doctor will be permitted to be taken for 4 weeks before the start of the clinical trial. The drug dosage prescribed by the doctor should be maintained throughout the trial period. Drugs used for the purpose of temporary treatment of other diseases will be administered together in consultation with the research doctor. When administering all concomitant drugs (including therapeutic drugs when other diseases or adverse events occur), information about the drug (product name, purpose of administration, dosage, administration period, etc.) should be recorded in detail in the case report form (CRF).

10.4.2 병용금지 약물

Prohibited drugs

병용금지 약물은 없으나, 연구자 판단에 따라 시험에 영향을 미칠 수 있는 약물은 금지할 수 있다. (예: 부신피질호르몬제제, 여성호르몬제제 등)

There are no drugs prohibited from concurrent use. However, drugs that may affect the test may be prohibited at the discretion of the researcher. (e.g., adrenocortical hormone preparations, female hormone preparations, etc.)

11. 관찰항목별 검사 및 시험 일정

Inspection and test schedule for each observation item

11.1 관찰항목별 방법

Method for each observation item

11.1.1 시험대상자 동의, 스크리닝 번호 부여 및 인구학적 조사

Subject consent, screening number assignment and demographic survey

본 임상시험을 실시하기에 앞서, 시험자는 '시험대상자 동의 설명서'에 관한 내용을 대상자 본인에게 설명하고, 대상자가 내용을 잘 이해한 것을 확인한 다음, 본인의 자유의사에 따른 임상시험 참가 동의를 문서로 받는다. 동의를 받는 순서에 따라 스크리닝 번호를 부여한 후 인구학적 정보를 조사한다.

The investigator will explain the contents of the 'informed consent form' to the subject before conducting this clinical trial. After confirming that the subject fully comprehends the content, the subject's written consent to participate in the clinical trial of his or her own free will will be obtained. Screening numbers will be assigned in the order that consent is received, and demographic data will be then investigated.

DJ-S-ZZZ [DJ: 대전대학교 대전한방병원, S: Screening의 첫 글자, ZZZ: 일련번호(001, 002~)] (ex: DJ-S-012: 대전대학교 대전한방병원에서 12번째 스크리닝 지원자)
DJ-S-ZZZ [DJ: Daejeon Korean Medicine Hospital of Daejeon University, S: Screening, ZZZ: serial number(001, 002~)] (ex: DJ-S-012: 12th screened participant at Daejeon Korean Medicine Hospital of Daejeon University)

기록사항은 서면 동의 여부 및 동의 일자, 시험대상자 이니셜, 성별, 생년월일, 연령, 주소 및 연락처 등이다.

Written consent, date of consent, initials, gender, date of birth, age, address, and contact information, etc. will be recorded.

11.1.2 병력 및 치료력 조사

Medical history and treatment history survey

임상시험에 들어가기 전에 시험대상자의 키, 체중, 병력 및 치료력 등에 대해 면담을 통하여 확인하고 증례기록지에 기록한다.

Before participating in a clinical trial, the test subject's height, weight, medical history, and treatment history will be confirmed via interview and recorded in the case report form (CRF).

11.1.3 시험대상자 적합성 평가
Test subject eligibility assessment

선정 및 제외기준에 따라 시험대상자로 적합한지 인구학적 조사, 병력 및 치료력 조사, 활력징후, Hoehn & Yahr scale, 임상검사실 검사 등을 통해 평가한다.

Suitability as a test subject will be evaluated using a demographic survey, medical and treatment history, vital signs, the Hoehn & Yahr scale, and clinical laboratory tests, depending on the inclusion and exclusion criteria.

11.1.4 시험대상자 식별코드 부여
Identification code to test subject

선정 및 제외기준에 따라 시험 참여가 적합한 자에 한해 시험대상자 식별코드를 부여한다.
시험대상자 식별코드는 다음 방법에 따라 기록한다.
Only those who are eligible to participate in the test based on the selection and exclusion criteria will be given a test subject identification code.
The following method will be used to record the test subject identification code.

DJ-E-ZZZ [DJ: 대전대학교 대전한방병원, E: Enrollment의 첫 글자, ZZZ: 일련번호(001, 002~)] (ex: DJ-E-015: 대전대학교 대전한방병원에서 15번째 등록 시험대상자)
DJ-E-ZZZ [DJ: Daejeon Korean Medicine Hospital of Daejeon University, E: Enrollment, ZZZ: serial number(001, 002~)] (ex: DJ-E-015: 12th enrolled participant at Daejeon Korean Medicine Hospital of Daejeon University)

11.1.5 임상검사항목
Clinical examination items

1) 활력징후
   Vital sign
   매 방문마다 급격한 체위 변동 없이 앉은 자세를 최소 3분 이상 유지한 상태에서 혈압, 맥박수를 측정한다. 체온은 실내온도에서 충분히 안정을 취한 상태에서 측정한다.
   At each visit, blood pressure and pulse rate will be measured while sitting for at least 3 minutes without any sudden movements. When the patient is sufficiently stable at room temperature, his or her body temperature will be measured.

2) 임상검사실 검사
   Laboratory tests

임상검사실 검사를 진행한다.
Conduct clinical laboratory tests.

- 혈액학적 검사: 헤모글로빈, 헤마토크릿, 적혈구수, 백혈구수, 혈소판수, HbA1c (3.5mL 채혈)
Hematological tests: hemoglobin, hematocrit, red blood cell count, white blood cell count, platelet count, HbA1c (3.5mL blood collection)

- 혈액화학적 검사: AST, ALT, γ-GTP, BUN, creatinine (5mL 채혈)
Blood chemical tests: AST, ALT, γ-GTP, BUN, creatinine (5mL blood collection)

- 소변검사: SG, pH, RBC(erythrocyte), WBC(leukocyte), Nitrite, Protein, Glucose, Ketone, Urobilinogen, Bilirubin
Urine test: SG, pH, RBC (erythrocyte), WBC (leukocyte), Nitrite, Protein, Glucose, Ketone, Urobilinogen, Bilirubin

3) 임신진단검사
Pregnancy test

폐경이 되지 않은 여성 대상자에 한해서 Urine HCG를 시행하며 결과는 음성이어야 한다. 단, Screening에서 menstruation에는 visit1에 시행하며, 방문16(대조군 방문3)에서 menstruation에는 방문17(대조군 방문4)에 시행한다.
Urine HCG testing will only be done on women who have not reached menopause, and the results must be negative. When menstruating during screening, however, Urine HCG will be performed on visit 1. Urine HCG will be performed on visit 17 (control group visit 4) if the subject is menstruating on visit 16 (control group visit 3).

4) 심전도 검사
Electrocardiography
12 lead EKG를 측정한다.
Measure 12 lead EKG.

5) 흉부 X-ray (chest-PA)검사
Chest X-ray (chest-PA) examination

흔히 서 있는 자세로 촬영하지만, 앉거나 누워서 촬영할 수도 있다. 촬영기를 가슴에 밀착시키고 촬영하는 동안 대부분 방사선사의 지시에 따라 숨을 깊이 들이마시고 참은 상태에서 촬영한다.
※ 선정 방문에서 임상검사실 검사, 심전도, 흉부 X-ray 검사는 대상자가 방문 14일 이내의 동일한 검사 결과를 제출할 경우, 해당 검사 결과로 대신할 수 있다.
It is usually filmed standing up, but it can also be filmed sitting or lying down. The camera is usually attached to the chest, and the radiologist's instructions are followed while

taking a deep breath and holding it.

※ Clinical laboratory tests, electrocardiograms, and chest X-rays can be replaced at the screening visit if the subject submits the same test results within 14 days of the visit.

6) Hoehn and Yahr Scale

파킨슨병의 운동 기능을 객관적으로 평가하기 위해 개발된 5단계 범주형 척도이다. 몸의 편측에 한정되어 안정 시 떨림이나 강직이 관찰되는 경우는 1단계, 양측의 떨림이나 강직이 관찰되는 경우 2단계로 평가한다. 몸의 양측에서 증상이 있으면서 자세불안정성이 있는 경우 3단계, 걷고 서기는 할 수 있으나 심하게 더뎌져서 혼자서는 생활이 어려운 상태를 4단계, 서기와 걷기를 할 수 없고 침대에 누워만 있는 상태를 5단계로 평가한다.

It is a 5-level categorical scale developed to objectively evaluate motor function in Parkinson's disease. If tremor or stiffness is observed at rest and is limited to one side of the body, it corresponds to stage 1. If bilateral tremor or stiffness is observed, evaluate to level 2. If there are symptoms on both sides of the body and postural instability, it is in stage 3. A condition in which the patient can walk and stand but is so slow that it is difficult to live alone corresponds to level 4. The condition in which one cannot stand or walk and is confined to bed is evaluated at level 5.

7) Mini Mental State Examination-K (MMSE-K)

MMSE-K는 12개 항목으로 구성된 설문지이다. 지남력, 기억력, 주의집중 및 계산, 언어기능, 이해 및 판단력을 진단하기 위한 평가로 신체적인 변화가 아닌 인지기능의 변화에 대해 초점을 맞춘 것이다.

무학이거나 문맹인 경우 평가가 어려운 점을 보완하기 위해 MMSE-K가 만들어졌으며 언어영역을 축소하고 이해와 판단 부분을 삽입하여 인지 능력을 평가할 수 있도록 만들어졌다.

지남력(시간, 주소, 장소), 기억회상, 주의집중 및 계산, 언어기능(사물이름, 3단계 명령, 복사, 반복), 이해 및 판단으로 구성된 12개 항목, 30점 만점으로 채점되며 18점 이하는 중증, 18~24점은 경증, 24점 이상은 정상인지상태 또는 경도인지장애로 판단할 수 있다.

MMSE-K is a questionnaire consisting of 12 items. It is an evaluation to diagnose orientation, memory, attention and calculation, language function, comprehension and judgment, and focuses on changes in cognitive function rather than physical changes. The MMSE-K was created to compensate for the difficulties in evaluating people who are uneducated or illiterate. It was crerequiring to evaluate cognitive ability by reducing the language area and inserting understanding and judgment parts. There are 12 items consisting of orientation (time, address, place), memory recall, attention and calculation, language function (object name, three-step command, copy, repetition), comprehension, and judgment. It is scored out of 30 points, with scores below 18 being considered severe, scores between 18 and 24 being mild, and scores above 24 being considered normal or mild cognitive impairment.

8) Movement Disorder Society Unified Parkinson’s Disease Rating Scale (MDS-UPDRS)

UPDRS는 파킨슨병환자 치료반응을 평가하기 위해 개발된 임상척도로 Goetz 등에 의해

MDS-UPDRS로 개정되어 임상에서 사용되고 있다. 주로 운동증상과 비운동증상 여부를 평가한다. 구성은 Part I(non-motor experiences of daily living)은 정신, 행동 및 정서, Part II(motor experiences of daily living)은 일상생활 활동, Part III(motor examination)은 운동기능검사, 그리고 Part IV(motor complications)은 이상운동 유무로 이루어져있다. 모든 항목의 점수는 높을수록 증세가 심한 것으로 평가된다.

UPDRS is a clinical scale developed to evaluate treatment response in Parkinson's disease patients. It was revised to MDS-UPDRS by Goetz et al. and is now used clinically. It mainly evaluates motor and non-motor symptoms. It consists of Part I (non-motor experiences of daily living), Part II (motor experiences of daily living), Part III (motor examination), and Part IV (motor complications). The higher the score for all items, the more severe the symptom.

9) International Physical Activity Questionnarie Short Form (IPAQ)

IPAQ(국제 신체활동 단축형 설문지)는 사람들이 평소에 하는 신체활동에 대해 알아보고자 지난 7일간 신체활동에 소모한 시간에 대한 7개 질문으로 구성되어 있다. 직장 및 집에서 하는 활동, 교통수단을 이용할 때 하는 활동, 여가시간에 시행하는 활동, 운동 또는 스포츠 모두를 포함하여 응답하도록 되어 있다.

IPAQ (International Physical Activity Questionnarie Short Form) consists of 7 questions about the time spent on physical activity over the past 7 days to find out about the physical activities people usually engage in. Responses are asked to include activities performed at work and at home, activities performed while using transportation, activities performed during leisure time, and exercise or sports.

10) 파킨슨 자가 검사지 (Parkinson Self Questionnaire, PSQ)

환자가 스스로 최근 한 달을 기준으로 신체의 불편한 부위 및 뻣뻣한 부위, 떨리는 부위, 느려지는 부위, 열감이 느껴지는 부위, 차가운 부위 등을 색칠하고 기입하여 자신의 상태를 표현할 수 있도록 한 검사지이다. 또한, 최근의 우울, 분노, 불안, 행복 등 감정 상태, 하루의 컨디션, 복용하는 약물을 기입할 수 있도록 하여 환자가 자신의 상태 변화를 확인하고 기록하는 용도로 활용할 수 있다.

This test sheet allows Parkinson's disease patients to express their condition by coloring and filling in areas of the body that are uncomfortable, stiff, trembling, slow, hot, and cold based on the previous month. Furthermore, patients can use it to monitor and record changes in their condition by entering their most recent emotional state, such as depression, anger, anxiety, or happiness, as well as their daily condition and medications.

11) Parkinson’s disease Sleep Scale (PDSS)

파킨슨병의 특성에 맞춘 수면장애를 평가하는 척도로 15문항으로 구성되어 있다. 각 항목은 전반적인 야간 수면의 질, 수면 시작과 불면의 지속 정도, 하지불안증후군, 야간 정신증상, 야뇨증세, 야간운동 증상, 수면 후 개운함, 일중 졸음 정도의 8개 카테고리를 반영한다. 총 15개 항목으로 각 항목은 10cm의 선으로 그어져 있고 증세가 가장 심함 0에서 증세가 하나도 없는 10까지 눈금이 매겨져 있으며 환자로 하여금 지난 방문과 비교하여 현재의 상태에 맞는 곳에 표시하도록 한다.

It is a 15-question scale for evaluating sleep disorders tailored to the characteristics of Parkinson's disease. Each item represents one of eight categories: overall nighttime sleep quality, sleep onset and duration of insomnia, restless legs syndrome, nocturnal mental symptoms, enuresis symptoms, nocturnal movement symptoms, feeling refreshed after sleep, and daytime sleepiness. There are 15 items in total, each drawn with a 10cm line and graded from 0 (the most severe symptom) to 10 (no symptom at all), and the patient is asked to mark the box that corresponds to their current condition compared to the previous visit.

12) Timed Up and Go (TUG) test
사람의 이동성을 평가하는 데 사용되는 간단한 테스트이며 정적 및 동적 균형을 모두 필요로 한다. 사람이 의자에서 일어나서 3미터를 걷고, 180도 회전하고, 다시 의자까지 걸어가서 180도 회전하면서 앉는 데 걸리는 시간을 사용한다.
It is a simple test that assesses both static and dynamic balance. We use the time it takes a person to get out of a chair, walk 3 meters, turn 180 degrees, return to the chair, turn 180 degrees, and sit down.

13) GAITRite 측정기기 (GAITRite measuring instrument)
GAITRite system은 보행의 시간, 공간적변수를 분석하는 기기로 총 길이 488cm, 센서가 받아들이는 길이 366cm 와 폭 61cm 인 전자식 보행매트 이다. 대상자가 보행매트 위를 걸으면, 감지 센서가 발의 압력에 반응하게 되고, 이들 정보를 직렬 인터페이스 케이블을 통해서 컴퓨터로 보내게 된다. 측정항목 중 보율(cadence)은 분당 보수(보수/분)이다. 속도(velocity)는 거리를 이동 시간으로 나눈 값(cm/s)이다. 보폭(step length)은 왼발의 발뒤꿈치 중심에서 오른발의 발뒤꿈치 중심까지의 거리(cm)이다. 한 발 지지 시간(single support)은 반대쪽 발이 스윙 하는 동안 한 발만 지면에 접촉해 있는 시간(초)이다. 이중 지지 시간(double support)은 두 발이 동시에 지면에 접촉해 있는 시간(초)이다. 스윙 시간(swing time)은 한 발이 지면에서 떨어져서 동일한 발이 지면에 접촉할 때까지의 시간(초)이다. 지지 시간(stance time)은 한 발이 지면에 접촉하여 떨어질 때까지의 시간(초)이다.
The GAITRite system analyzes the temporal and spatial variables of walking. It is a 488cm long electronic walking mat with a sensor-accepted length of 366cm and a width of 61cm. When the subject walks on the walking mat, the sensor detects foot pressure and transmits it to the computer via a serial interface cable. Among the measurement items, cadence is steps per minute (steps/minute). Velocity is the distance divided by the travel time (cm/s). Step length is the distance (cm) from the center of the heel of the left foot to the center of the heel of the right foot. Single support time is the number of seconds that one foot is in contact with the ground while the opposite foot swings. Double support is the time in seconds that both feet are in contact with the ground at the same time. Swing time is the time (in seconds) from when one foot leaves the ground until the same foot touches the ground. Stance time is the time (in seconds) from when one foot touches the ground to when it falls.

- 제조사: CIR Systems Inc.
- 제품명: GAITRite Electronic Walkway Platinum
- 품목명: 보행분석계
- 측정 위치: 지면에 접촉하는 발바닥
- 측정 방법: 대상자가 보행매트 위를 걸으면, 본 기기는 내장된 압력 센서를 이용하여 보행주기를 포함한 COP, 보행 중 압력 분포, 시간 및 거리 등을 측정하여 환자의 보행 능력을 기록하고 분석한다. 측정된 파라미터들은 PC와 연결하여 모니터링 할 수 있으며, 이를 통해 환자의 보행 능력의 감퇴 여부와 치료에 따른 기능 회복을 객관적으로 확인 할 수 있다.
- Manufacturer: CIR Systems Inc.
- Product Name: GAITRite Electronic Walkway Platinum
- Item name: Gait analysis system
- Measurement location: sole of the foot in contact with the ground
- Measurement method: When a subject walks on a walking mat, this device uses a built-in pressure sensor to measure COP including the gait cycle, pressure distribution during walking, time and distance, etc. to record and analyze the patient's walking ability. The measured parameters can be monitored by connecting to a PC, and through this, it is possible to objectively check whether the patient's walking ability has decreased and whether functional recovery has occurred following treatment.

14) Functional Near-Infrared Spectroscopy(fNIRS) 측정기기 (fNIRS measuring instrument)
NIRx회사의 NIRX 모델로 fNRIS기기 사용하기 쉬운 모듈 형 및 근적외선 분광법(fNIRS)으로 대뇌피질에서 신경활성화하는 oxy-deoxy-그리고 총 헤모글로빈의 변화를 통해 혈역학적 반응을 측정하는 기계로 fNIRS CAP을 활용하여 대상자의 전두엽 및 운동영역 혈류 변화를 측정한다.
The NIRX model from NIRx is a machine that measures hemodynamic responses through changes in oxy-, deoxy-, and total hemoglobin that activate nerves in the cerebral cortex using an easy-to-use modular fNRIS device and near-infrared spectroscopy (fNIRS). fNIRS CAP is used to measure changes in blood flow in the subject's frontal lobe and motor area.

- 제조사: NIRx Medical Technologies LLC
- 모델명: NIRScoutX
- 측정 위치: 두피
- 측정 방법: CAP을 통해 대상자의 두피에 센서를 접촉시킨 후 대상자의 전두엽 및 운동영역의 혈류 변화를 측정한다.
- Manufacturer: NIRx Medical Technologies LLC
- Model name: NIRScoutX
- Measurement location: scalp
- Measurement method: After contacting the sensor with the subject's scalp through CAP, changes in blood flow in the subject's frontal lobe and motor area are measured.

15) 스마트밴드 (Smart band)

걸음수, 수면시간, 수면패턴, 심박수, 생활습관 정보 등의 수집은 Samsung Galaxy Fit2(삼성 갤럭시 핏2) 스마트밴드 및 Galaxy Wearable 및 Samsung Health 앱을 활용한다.

The Samsung Galaxy Fit2 smart band and Galaxy Wearable and Samsung Health apps are used to collect step count, sleep time, sleep pattern, heart rate, and lifestyle information.

- 제조사: Huaqin(제조자), 삼성전자(주), 대한민국(수입자)
- 제품명: Samsung Galaxy Fit2(삼성 갤럭시 핏2)
- 품목명: 특정소출력 무선기기(무선데이터통신시스템용 무선기기)
- 측정 위치: 손목
- 측정 방법: Galaxy Wearable 및 Samsung Health 앱을 스마트폰(안드로이드/iOS 공통)에 설치하고 삼성 계정 회원가입 후 로그인을 한다. 대상자의 핸드폰에 Galaxy Wearable 및 Samsung Health 앱을 애플리케이션을 다운받은 후 ID를 가입하여 승인 받도록 한다. 초기화된 Samsung Galaxy Fit2를 대상자의 손목에 채우고 휴대폰과 동기화를 시킨다. Galaxy Wearable 및 Samsung Health 앱은 스마트폰을 사용하여 대상자의 걸음수, 수면시간, 수면패턴, 심박수 등을 자동으로 측정하고, 스마트밴드 설치에 동의한 연구 완료 대상자에 한하여 Visit 17 (대조군의 경우 Visit 4) 방문 시 어플리케이션을 통해 측정 데이터를 csv 파일을 일괄적으로 직접 다운받아 임상시험센터 컴퓨터에 저장하여 보관한다. 단, 중도탈락자의 경우 데이터를 수집하지 않으며, 스마트밴드를 지급한 대상자에 한하여 방문시마다 밴드 착용여부를 확인한다.
- Manufacturer: Huaqin (manufacturer), Samsung Electronics Co., Ltd., Korea (importer)
- Product Name: Samsung Galaxy Fit2
- Item name: Specific low-power wireless device (wireless device for wireless data communication system)
- Measurement location: wrist
- Measurement method: Install the Galaxy Wearable and Samsung Health apps on your smartphone (both Android/iOS), sign up for a Samsung account, and log in. After downloading the Galaxy Wearable and Samsung Health apps to the subject's cell phone, sign up for an ID and receive approval. The initialized Samsung Galaxy Fit2 is placed on the subject's wrist and synchronized with the mobile phone. The Galaxy Wearable and Samsung Health apps use a smartphone to automatically measure the subject's step count, sleep time, sleep pattern, and heart rate. For those who have completed the study and agreed to the installation of the smart band, when visiting Visit 17 (Visit 4 for the control group), the measurement data will be directly downloaded as a csv file through the application and stored on the clinical trial center computer. However, data will not be collected for those who drop out, and only those who have been provided with a smart band will be checked to see if they are wearing the band at each visit.

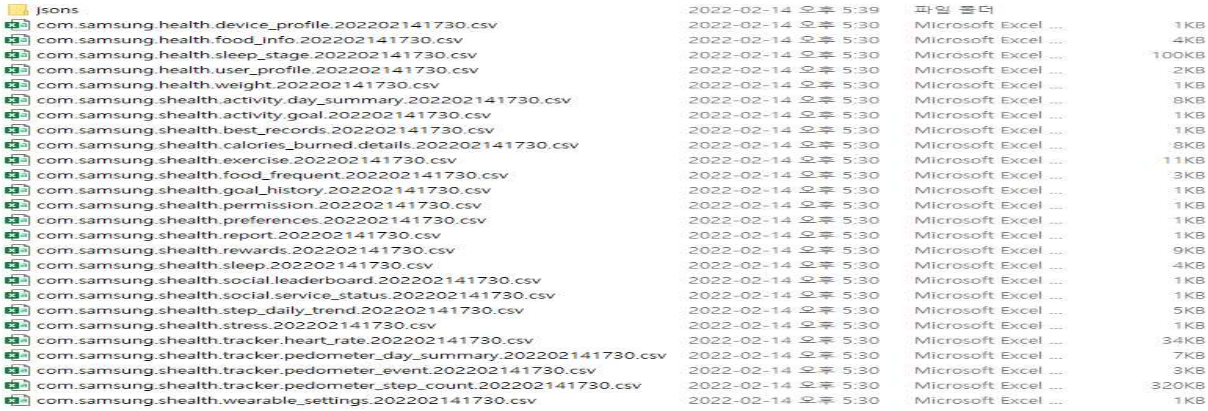

jsons	2022-02-14 오후 5:39	파일 폴더	
com.samsung.health.device_profile.202202141730.csv	2022-02-14 오후 5:30	Microsoft Excel ...	1KB
com.samsung.health.food_info.202202141730.csv	2022-02-14 오후 5:30	Microsoft Excel ...	4KB
com.samsung.health.sleep_stage.202202141730.csv	2022-02-14 오후 5:30	Microsoft Excel ...	100KB
com.samsung.health.user_profile.202202141730.csv	2022-02-14 오후 5:30	Microsoft Excel ...	2KB
com.samsung.health.weight.202202141730.csv	2022-02-14 오후 5:30	Microsoft Excel ...	1KB
com.samsung.shealth.activity.day_summary.202202141730.csv	2022-02-14 오후 5:30	Microsoft Excel ...	8KB
com.samsung.shealth.activity.goal.202202141730.csv	2022-02-14 오후 5:30	Microsoft Excel ...	1KB
com.samsung.shealth.best_records.202202141730.csv	2022-02-14 오후 5:30	Microsoft Excel ...	1KB
com.samsung.shealth.calories_burned.details.202202141730.csv	2022-02-14 오후 5:30	Microsoft Excel ...	8KB
com.samsung.shealth.exercise.202202141730.csv	2022-02-14 오후 5:30	Microsoft Excel ...	11KB
com.samsung.shealth.food_frequent.202202141730.csv	2022-02-14 오후 5:30	Microsoft Excel ...	3KB
com.samsung.shealth.goal_history.202202141730.csv	2022-02-14 오후 5:30	Microsoft Excel ...	1KB
com.samsung.shealth.permission.202202141730.csv	2022-02-14 오후 5:30	Microsoft Excel ...	1KB
com.samsung.shealth.preferences.202202141730.csv	2022-02-14 오후 5:30	Microsoft Excel ...	1KB
com.samsung.shealth.report.202202141730.csv	2022-02-14 오후 5:30	Microsoft Excel ...	1KB
com.samsung.shealth.rewards.202202141730.csv	2022-02-14 오후 5:30	Microsoft Excel ...	9KB
com.samsung.shealth.sleep.202202141730.csv	2022-02-14 오후 5:30	Microsoft Excel ...	4KB
com.samsung.shealth.social.leaderboard.202202141730.csv	2022-02-14 오후 5:30	Microsoft Excel ...	1KB
com.samsung.shealth.social.service_status.202202141730.csv	2022-02-14 오후 5:30	Microsoft Excel ...	1KB
com.samsung.shealth.step_daily_trend.202202141730.csv	2022-02-14 오후 5:30	Microsoft Excel ...	5KB
com.samsung.shealth.stress.202202141730.csv	2022-02-14 오후 5:30	Microsoft Excel ...	1KB
com.samsung.shealth.tracker.heart_rate.202202141730.csv	2022-02-14 오후 5:30	Microsoft Excel ...	34KB
com.samsung.shealth.tracker.pedometer_day_summary.202202141730.csv	2022-02-14 오후 5:30	Microsoft Excel ...	7KB
com.samsung.shealth.tracker.pedometer_event.202202141730.csv	2022-02-14 오후 5:30	Microsoft Excel ...	3KB
com.samsung.shealth.tracker.pedometer_step_count.202202141730.csv	2022-02-14 오후 5:30	Microsoft Excel ...	320KB
com.samsung.shealth.wearable_settings.202202141730.csv	2022-02-14 오후 5:30	Microsoft Excel ...	1KB

Samsung Health 어플리케이션을 통해 다운받은 스마트워치 측정 데이터 예시
Example of smartwatch measurement data downloaded through the Samsung Health application

16) 홍채검사 1 (Iris examination 1)

홍채는 복잡한 섬유막 구조로 되어있고 신경을 통하여 대뇌와 신체 각 부위에 연결되어 있기 때문에 신체 내의 각 조직, 장기에서 일어나는 화학적, 물리적 변화에 따른 정보가 전달되어 섬유조직의 형태를 변화시키게 된다. 이러한 홍채 구조 및 조직 질감 변화에 대한 분석을 통해 건강 상태 확인 및 신체의 체질 구별이 가능하다. 홍채검사 1은 다음 장비를 이용하여 시행한다.

Because the iris has a complex fibrous membrane structure and is connected to the cerebrum and each part of the body via nerves, information is transmitted based on chemical and physical changes that occur in each tissue and organ within the body, changing the shape of the fibrous tissue. It is possible to check health status and distinguish body constitution by analyzing changes in iris structure and tissue texture. The following equipment is used for Iris test 1.

- 제조사: AnMo Electronics Corporation
- 제품명: AM4113-RUT Dino-Lite Digital Iriscope 홍채현미경
- 품목명: 홍채 카메라
- 측정 위치: 홍채
- 측정 방법: 콘택트렌즈를 착용하지 않은 상태에서, 대상자에게 정면을 바라보게 한 뒤 정해진 절차에 따라 동공 및 홍채의 상하좌우 가장자리가 모두 나온 영상을 좌안, 우안 차례대로 획득한다. 흔들리지 않은 사진이 촬영될 때까지 반복한다.
- Manufacturer: AnMo Electronics Corporation
- Product Name: AM4113-RUT Dino-Lite Digital Iriscope Iris Microscope
- Item name: Iris camera
- Measurement location: Iris
- Measurement method: Without contact lenses, have the subject look straight ahead and capture images of the pupil as well as the top, bottom, left, and right edges of the iris for the left and right eyes, respectively. Repeat until a clear image is obtained.

17) 홍채검사 2 (Iris examination 2)
홍채검사 2는 다음 장비를 이용하여 시행한다.
Iris examination 2 is performed using the following equipment.
- 제조사: IriTech Inc./Iri screen
- 품목명: 전안부 촬영장치
- 측정 위치: 홍채
- 측정 방법: 콘택트렌즈를 착용하지 않은 상태에서, 대상자에게 정면을 바라보게 한 뒤 정해진 절차에 따라 동공 및 홍채의 상하좌우 가장자리가 모두 나온 영상을 좌안, 우안 차례대로 획득한다. 흔들리지 않은 사진이 촬영될 때까지 반복한다.
- Manufacturer: IriTech Inc./Iri screen
- Item name: Anterior segment imaging device
- Measurement location: Iris
- Measurement method: Without contact lenses, have the subject look straight ahead and capture images of the pupil as well as the top, bottom, left, and right edges of the iris for the left and right eyes, respectively. Repeat until a clear image is obtained.

18) EuroQoL five dimension (EQ-5D)
EQ-5D는 1987년에 설치된 EuroQol 그룹에 의해 설치되고 연속적으로 개발되었다. Korean EuroQol-5 dimension(KEQ-5D)는 건강관련 삶의 질을 효용(utility)으로 측정하는 도구로 다차원적 선호도 근거 건강 관련 삶의 질 측정도구(multidimensional preference based HRQOL measure)이다. 본 시험에서는 교차-문화적 적응 작업과 확인 과정이 완료된 한국어판 EQ-5D를 사용한다. EQ-5D는 두 가지 방법으로 삶의 질을 평가한다. 먼저 기술체계(descriptive system)를 이용하여 운동능력, 자기관리, 일상활동, 통증/불편, 불안/우울 등 5개 영역에서 삶의 질을 평가한다. 각 영역은 5단계로 평가한다. 따라서 점수가 낮을수록 HRQoL은 높다. 둘째로 20cm의 세로 선으로 구성된 유추 척도(visual analogue scale: VAS)를 이용하여, 전반적인 건강상태에 대한 평가를 시행한다. 점수는 최저의 건강상태를 0점으로 최고의 건강상태를 100점으로 표시하는데, 점수가 높을수록 HRQoL이 높다.
The EuroQol group, which was founded in 1987, established and developed the EQ-5D. Korean EuroQol-5 dimension (KEQ-5D) is a multidimensional preference-based health-related quality of life measurement tool (HRQOL measure) that measures health-related quality of life through utility. This test employs the Korean version of the EQ-5D, which has been cross-culturally adapted and verified. The EQ-5D assesses life satisfaction in two ways. First, a descriptive system is used to assess the quality of life in five areas: exercise ability, self-management, daily activities, pain/discomfort, and anxiety/depression. Each area is graded on a 5-point scale. As a result, the lower the score, the better the HRQoL. Second, a visual analog scale (VAS) with a 20cm vertical line is used to assess overall health status. The score assigns a value of 0 to the worst health status and a value of 100 to the best health status. The higher the HRQoL, the higher the score.

EQ-5D는 area under the curve 방법을 사용하여 Quality adjusted life years (QALYs)로 계산한다. 따라서, QALYs는 삶의 질(quality of life)와 양(quantity of life)를 고려한다. 일반적으로 1년 동안의 1 QALY는 1년 동안 완벽한 건강상태를 의미한다.
EQ-5D is calculated as Quality adjusted life years (QALYs) using the area under the curve method. Therefore, QALYs consider both quality of life and quantity of life. Generally, 1 QALY in 1 year means perfect health for 1 year.

'QALYs differences × national willingness to pay – cost differences' 식으로 net monetary benefit (NMB)를 계산한다.
Net monetary benefit (NMB) is calculated using the formula 'QALYs differences × national willingness to pay – cost differences'.

19) EuroQol Visual analog scale (EQ-VAS)
전반적인 건강상태에 대한 평가를 시행한다. 점수는 최저의 건강상태를 0점으로 최고의 건강상태를 100점으로 표시하며 점수가 높을수록 HRQoL이 높다.
Make an evaluation of your overall health. The score gives a 0 to the worst health status and a 100 to the best health status. The higher the score, the better the HRQoL.

20) 비용 측정 (Cost measurement)
시험군과 대조군의 효과가 비용대비 유의한 차이가 있는지를 평가하기 위하여 직접의료비용, 직접비의료비용, 생산성손실비용을 조사한다.
Direct medical costs, direct non-medical costs, and productivity loss costs are studied to determine whether there is a significant difference in the effectiveness of the experimental and control groups when compared to the cost.

21) 장내미생물 검사 (Gut microbiome examination)
장내미생물 분석을 위해 대상자는 미리 배부한 분변 채취 키트를 통해 Visit 1 방문 전 3일 이내 획득한 분변을 가져오도록 한다. 대상자는 대변을 보기 전 방광을 비우고, 소변이나 변기 물이 대변에 묻지 않도록 하여 미리 제공된 분변 채취 키트를 통해 대변을 채취한다. 장내미생물 분석을 위해 검체를 포장하여 분석기관으로 보낸다. 수집된 분변 샘플을 대상으로 DNA를 분리 정제한다. 정제한 DNA를 이용하여 차세대 염기서열 분석 방법(NGS)을 이용하여 미생물 분포를 분석한다. 분변 채취는 week 1, 8 총 2회 시행한다.
Subjects are asked to bring feces collected within three days of Visit 1 using the pre-assigned fecal collection kit for gut microbiome analysis. Before defecating, the subject empties the bladder, avoids contacting the stool with urine or toilet water, and collects stool with a fecal collection kit provided in advance. The sample is packaged and sent to an analysis facility for analysis of gut microbiome. DNA is extracted and purified from fecal samples. Purified DNA and next-generation sequencing (NGS) are used to study the distribution of microorganisms. The feces are collected twice a week, on week 1 and week 8.

- 종류: 분변

- 인체유래물 연구 동의: 인체유래물 취득 전 인체 유래물 연구 동의서를 득한다.
- 수집방법: (주) 랩지노믹스에서 제공한 kit를 사용한다.
- 보관방법: 분변에 담긴 튜브는 동봉된 상자에 적힌 (주) 랩지노믹스 주소로 택배 발송을 한다. 해당 튜브는 상온에서 미생물증식 억제 보존액이 담겨있어서 택배발송으로 이동시에도 장내미생물에 영향을 미치지 않는다.
- Type: feces
- Consent for research on human specimens: Before acquiring human specimens, obtain consent for research on human specimens.
- Collection method: Use the kit provided by Lab Genomics Co., Ltd.
- Storage method: Tubes containing feces are couriered to the Lab Genomics Co., Ltd. address written on the enclosed box. The tube contains a preservation solution that inhibits microbial growth at room temperature, ensuring that intestinal microorganisms are not harmed even when delivered by courier.

11.1.7 순응도 평가
Compliance assessment

중재에 대한 순응도를 아래와 같이 평가하도록 한다.
시험 참여 기간 동안의 전체 순응도는 75% 이상이어야 하며, 75% 미만인 경우 해당 시험대상자는 순응도가 나쁜 것으로 간주하여 PP분석군에서 제외한다.
The following criteria should be used to assess compliance with the intervention.
During the trial period, the overall compliance rate must be greater than 75%. If it is less than 75%, the subject is deemed to be noncompliant and is removed from the PP analysis group.

$$\text{순응도(compliance)} = \frac{\text{실제 시행된 중재횟수 (actual number of interventions)}}{\text{계획된 중재횟수 (planned number of interventions)}} \times 100$$

11.1.8 이상반응 조사
Adverse event investigation

연구책임자 혹은 연구의사는 임상시험 참여 후 나타나는 이상반응 여부를 방문 시 마다 시험대상자에 대한 진찰로서 관찰하여 시험 치료법과의 인과관계에 대하여 ‘이상반응 기록표’에 기록한다. 이상반응 발현 시 발현일 및 소실일, 이상반응의 정도 및 결과, 시험 치료법과 관련하여 취해진 조치 및 시험 치료법과의 인과관계, 시험 치료법 이외 의심되는 원인, 이상반응에 대한 치료 여부 및 내용 등을 상세히 기록한다.
At each visit, the principal investigator or research doctor examines the test subject to determine whether any adverse events occur following participation in the clinical trial and records the causal relationship with the test treatment in the 'adverse event record table.' When an adverse event occurs, the date of onset and disappearance, the extent and results of the adverse event, the measures taken in relation to the test treatment and the

causal relationship with the test treatment, suspected causes other than the test treatment, whether or not the adverse event is treated, and its contents are all meticulously recorded.

11.1.9 활력징후에 대한 점검
Checking vital signs

각 방문 시 체온, 맥박, 호흡수 및 혈압을 기록하고 이상이 있다면 중재와의 인과관계를 평가한다.

Body temperature, pulse rate, respiratory rate, and blood pressure are all recorded at each visit, and if there are any abnormalities, the causal relationship with intervention is investigated.

11.1.10 임상검사실 검사를 통한 점검
Inspection through clinical laboratory tests

시험 시작 후 8주(16회차) 방문 시에 임상검사실 검사를 시행하고 비정상적인 변화는 추적 검사를 시행하도록 하며 예상 원인을 기록한다. 또한, 임상검사실 검사 시 검사내용에 지장을 줄 수 있는 요인(음주, 심각한 피로 등)이 있었는지 면담을 통하여 기록한다.

A clinical laboratory test is performed at the 8-week (16th) visit after starting the test, any abnormal changes are followed up on, and the probable cause is recorded. Furthermore, any factors that may interfere with test results (drinking, extreme fatigue, etc.) are recorded through interviews during clinical laboratory tests.

11.2 시험 일정

Trial Schedule

11.2.1 시험 일정표

Trial schedule (table)

임상시험 일정 (Clinical Trial Schedule)

기간period	스크리닝screening & 처치treatment						추적관찰 follow-up
주week	0~4				5~8		12
방문visit[1)]	Screening	experimental group 1	2~7W±1D	8W±1D	9~15W±1D	16W±1D	17W±7D
		control group 1		2W±7D		3W±7D	4W±7D
동의서 취득<br>Obtain informed consent form	●						
Hoehn & Yahr	●						
MMSE-K	●						
인구학적 조사<br>Demographic survey	●						
심전도 검사<br>Electrocardiography	●						
흉부 x-ray 검사<br>Chest x-ray examination	●						
홍채검사 1 & 2<br>Iris examination 1 & 2	●						
임상검사실 검사[2)]<br>lab test	●					●	
병력 및 치료력 조사<br>Medical history and treatment history survey	●	●	●	●	●	●	●
활력징후 측정<br>Vital signs	●	●	◇	●	◇	●	●
선정/제외기준 확인<br>Check inclusion/exclusion criteria	●						
무작위 배정<br>Random assignment		●					
장내미생물검사<br>Gut microbiome		●				●	
신장/체중 측정[3)4)]<br>Height/weight measurements		●		●		●	●
병용약물 확인<br>Check concomitant medications		●	●	●	●	●	●
이상반응 확인<br>Check for adverse events		●	●	●	●	●	●
MARS-PD[4)]		◇	◇	◇	◇	◇	
MDS-UPDRS		●		●		●	●

**IPAQ**		●		●		●	●
**PSQ**		●		●		●	●
**PDSS**		●		●		●	●
**TUG**		●				●	●
**GAITRite**		●				●	●
**fNIRS**[5)]		●				●	●
**효용측정**[6)] Utility measurement		●		●		●	●
**비용측정**[7)] Cost measurement		●		●		●	●
**스마트밴드**[8)] Smart Band		●					●
**순응도 확인** Check compliance			●	●	●	●	●
**방문일정 교육** Visit schedule information		●	◇	●	◇	●	

9) Screening은 방문1로부터 0-7일 이내에 행해져야 한다. 방문1은 Baseline 방문이다. (방문허용일: 시험군은 Baseline으로부터 주±1일/ 방문17에서는 Baseline으로부터 12주 ±3일, 대조군은 Baseline으로부터 각 방문마다 허용일 ±7을 둔다.)
Screening must be performed within 0-7 days from Visit 1. Visit 1 is the baseline visit. (Visit Allowed Date: For the experimental group, ±1 day per week from the baseline, for visit 17, 12 weeks ±3 days from the baseline, for the control group, ±7 days per week are allowed from the baseline for each visit.)

10) 임상검사실 검사, 임신진단검사: 임상시험 대상자는 내원하여 다음의 항목을 검사한다. 방문1 기준 14일 이내의 검사결과가 있다면 적용 가능하며, 시험자의 판단에 따라 비정상적인 결과에 대한 재검사를 시행할 수 있다.
Clinical laboratory test, pregnancy diagnostic test: Clinical test subjects visit the hospital and test the following items. It is applicable if there are test results within 14 days of visit 1, and re-testing can be performed for abnormal results at the investigator's discretion.
  - 혈액학적 검사: 헤모글로빈, 헤마토크릿, 적혈구수, 백혈구수, 혈소판수, HbA1c
    Hematological tests: hemoglobin, hematocrit, red blood cell count, white blood cell count, platelet count, HbA1c
  - 혈액화학적 검사: AST, ALT, γ-GTP, BUN, creatinine
    Blood chemical tests: AST, ALT, γ-GTP, BUN, creatinine
  - 소변검사: SG, pH, RBC(erythrocyte), WBC(leukocyte), Nitrite, Protein, Glucose, Ketone, Urobilinogen, Bilirubin
    Urine test: SG, pH, RBC (erythrocyte), WBC (leukocyte), Nitrite, Protein, Glucose, Ketone, Urobilinogen, Bilirubin
  - 임신진단검사: 폐경이 되지 않은 여성 대상자는 Urine HCG를 시행하며 결과는 음성이어야 한다. 단, Screening에서 menstruation에는 visit1에 시행하며, 방문16(대조군 방문3)에서 menstruation에는 방문17(대조군 방문4)에 시행한다.
    Pregnancy diagnostic test: Urine HCG is performed on female subjects who have not entered menopause, and the results must be negative. However, in screening, menstruation is conducted on visit 1, and menstruation on visit 16 (control group visit 3) is performed on visit 17 (control group visit 4).

11) 신장은 방문1에서만 측정하며, 체중은 방문1, 8, 16, 17에 측정하여 기록한다. 신장은 0.1cm, 체중은 0.1kg 단위까지 반올림하여 측정한다.
Height is measured only at visit 1, and weight is measured and recorded at visits 1, 8, 16, and 17. Height is measured to the nearest 0.1 cm and weight is measured to the nearest 0.1 kg.

12) MARS-PD와 ◇로 표시한 항목은 모두 시험군에 한해 실시한다.
MARS-PD and all items marked with ◇ are administered only to the experimental group.

13) fNIRS 검사 시에 보행검사(GAITRite)를 수행한다.
During the fNIRS test, a gait test (GAITRite) is performed.

14) 효용측정은 EQ-5D, EQ-VAS를 사용하여 수행한다.
Utility measurement is performed using EQ-5D and EQ-VAS.

15) 비용측정은 직접의료비, 직접비의료비, 간접비의 조사를 위하여 별도 개발된 비용 조사지를 사용하여 조사한다.
Cost measurement is conducted using a separate cost survey form developed to investigate direct medical costs, non-direct medical costs, and indirect costs.

16) 스마트밴드는 착용을 원하는 대상자에게 선택적으로 제공하며, 연구 완료자에 한하여 12주차에 데이터를 일괄 수집한다. 단, 중도탈락자의 경우 데이터를 수집하지 않으며, 스마트밴드를 지급한 대상자에 한하여 방문시마다 밴드 착용여부를 확인한다.
Smart bands are optionally provided to those who wish to wear them, and data is collected in batches in the 12th week only for those who complete the study. However, data will not be collected for those who drop out, and only those who have been provided with a smart band will be checked to see if they are wearing the band at each visit.

11.2.2 선정 방문(0주)
Screening visit

내원 혹은 전화로 임상시험 참여를 신청한 지원자를 대상으로 다음 순서에 따라 스크리닝을 한다.

지원자가 서명한 시험대상자 동의서를 얻는다. 이 시험에 참여하는 지원자는 시험대상자 동의를 얻기 위한 설명을 듣고 자유의사에 의해 동의서에 서면 동의를 한 후 시험에 참여할 수 있다.

Applicants who apply in person or by phone to participate in clinical trials are screened in the order listed below.

Obtain a signed subject consent form from the applicant. Applicants who wish to participate in this trial must first receive an explanation for obtaining test subject consent and provide written consent in the form of their own free will.

스크리닝 번호 부여 후, 인구학적 정보 및 병력, 치료력 등에 대한 문진을 실시한다.

- 인구학적 정보: 시험대상자 이니셜, 성별, 생년월일, 연령, 흡연력, 음주력 등
- 병력 및 치료력 조사: 주소증, 발병일, 발병인자, 현병력, 과거력, 약물투여력, 음주·흡연력
- 활력징후 측정: 혈압, 맥박, 체온

Following the assignment of a screening number, a questionnaire containing demographic information, medical history, and treatment history is administered.

- Demographic data: test subject initials, gender, date of birth, age, smoking and drinking history, and so on.
- Medical and treatment history investigation: chief complaint, date of onset, onset factors, present illness, past history, medication history, drinking/smoking history
- Vital signs monitoring: blood pressure, pulse rate, and body temperature

Hoehn & Yahr scale, MMSE-K 검사를 실시한다.

흉부 x-ray 검사, 심전도 검사를 실시한다.

임상검사실 검사를 시행한다.

- 혈액학적 검사: 헤모글로빈, 헤마토크릿, 적혈구수, 백혈구수, 혈소판수, HbA1c
- 혈액화학적 검사: AST, ALT, γ-GTP, BUN, creatinine
- 소변검사: SG, pH, RBC(erythrocyte), WBC(leukocyte), Nitrite, Protein, Glucose, Ketone, Urobilinogen, Bilirubin
- 홍채검사 1, 홍채검사 2를 시행한다.

Conduct the Hoehn & Yahr scale and MMSE-K tests.

Perform a chest x-ray and electrocardiogram.

Perform clinical laboratory tests.

- Hematological tests: hemoglobin, hematocrit, red blood cell count, white blood cell count, platelet count, HbA1c
- Blood chemical tests: AST, ALT, γ-GTP, BUN, creatinine
- Urine test: SG, pH, RBC (erythrocyte), WBC (leukocyte), Nitrite, Protein, Glucose, Ketone, Urobilinogen, Bilirubin

- Perform Iris examination 1 and iris examination 2.

※ 선정 방문에서 임상검사실 검사, 심전도, 흉부 X-ray(chest-PA) 검사는 대상자가 방문 14일 이내의 동일한 검사 결과를 제출할 경우, 해당 검사 결과로 대신할 수 있다.
임신진단검사로 폐경이 되지 않은 여성 대상자에 한해서 Urine HCG를 시행한다. 단, menstruation에는 visit1에 시행한다.
종합적으로 선정, 제외기준을 확인한다.
다음 방문일을 지정한다.

※ Clinical laboratory tests, electrocardiograms, and chest X-ray (chest-PA) tests can be replaced at the screening visit with existing test results if the subject submits the same test results within 14 days of the visit.
Through a pregnancy test, urine HCG is only performed on women who have not reached menopause. In the case of menstruation, however, urine HCG is measured during the visit1.
Comprehensively check inclusion and exclusion criteria.
Specify the next visit date.

11.2.3 시험 시행
Trial conduct

11.2.3.1 방문 1 (0주)
Visit 1 (week 0)

선정방문 후 무작위배정 번호를 부여하고 다음의 평가를 실시한다.
방사선검사, 임상검사실 검사 등 선정 방문의 결과를 확인한다.
무작위배정을 실시하여 식별 코드를 부여한다.
시험대상자의 병력이나 치료력의 변화여부를 확인한다.
병용약물 변화여부를 확인한다.
활력징후, 신장/체중을 측정한다.
중재 시술 전 MDS-UPDRS, IPAQ, 파킨슨 자가 검사지, PDSS 검사를 실시한다.
대상자에게 스마트밴드를 제공하고 사용법을 안내한다. (스마트밴드 착용에 동의한 대상자에 한함.)
보행검사(TUG 및 GAITRite)를 실시하며 fNIRS 검사를 수행한다.
시험군은 MARS-PD 치료를 실시한다.
비용측정 및 효용측정(EQ-5D, EQ-VAS)을 실시한다.
장내미생물 검사를 실시한다.
다음 방문일을 교육한다.
After the selection visit, a randomization number is assigned and the following evaluation is conducted.
Check the results of selected visits, including radiology tests and clinical laboratory tests.
Random allocation is performed and an identification code is assigned.
Check for changes in the test subject's medical history or treatment history.
Check for changes in concomitant medications.

Measure vital signs and height/weight.
Before interventional treatment, MDS-UPDRS, IPAQ, Parkinson's self-test questionnaire, and PDSS tests are performed.
Provide a smart band to the subject and guide them on how to use it. (Limited to those who agree to wear the smart band.)
Gait tests (TUG and GAITRite) are performed and fNIRS tests are performed.
The experimental group will receive MARS-PD treatment.
Conduct cost measurement and utility measurement (EQ-5D, EQ-VAS).
Perform gut microbiome examination.
Educate about the next visit date.

11.2.3.2 방문 2~7 (0~4주)

Visits 2-7 (weeks 0-4)

시험대상자의 병력이나 치료력의 변화여부를 확인한다.
병용약물 변화여부를 확인한다.
활력징후를 측정한다.
이상반응을 확인한다.
시험대상자의 병력이나 치료약물의 변화여부를 확인하여 기록한다.
시험대상자의 스마트밴드 착용여부를 확인한다. (스마트밴드 착용에 동의한 대상자에 한함.)
시험군은 MARS-PD 치료를 실시한다.
해당 방문까지의 순응도를 확인한다.
다음 방문일을 지정한다(단, 계획된 방문일로부터 계산한다).
Check for changes in the test subject's medical history or treatment history.
Check for changes in concomitant medications.
Measure vital signs.
Check for adverse events.
Any changes in the test subject's medical history or treatment medications are checked and recorded.
Check whether the test subject is wearing a smart band. (Limited to those who agree to wear the smart band.)
The experimental group will receive MARS-PD treatment.
Check compliance up to the visit.
Specify the next visit date (however, it is calculated from the planned visit date).

11.2.3.3 방문 8 (4주)

Visit 8 (week 4)

시험대상자의 병력이나 치료력의 변화여부를 확인한다.
병용약물 변화여부를 확인한다.
활력징후, 체중을 측정한다.
이상반응을 확인한다.

시험대상자의 병력이나 치료약물의 변화여부를 확인하여 기록한다.
시험대상자의 스마트밴드 착용여부를 확인한다. (스마트밴드 착용에 동의한 대상자에 한함.)
중재 시술 후 MDS-UPDRS, IPAQ, 파킨슨 자가 검사지, PDSS 검사를 실시한다.
비용측정 및 효용측정(EQ-5D, EQ-VAS)을 실시한다.
해당 방문까지의 순응도를 확인한다.
다음 방문일을 지정한다(단, 계획된 방문일로부터 계산한다).
Check for changes in the test subject's medical history or treatment history.
Check for changes in concomitant medications.
Measure vital signs and weight.
Check for adverse events.
Any changes in the test subject's medical history or treatment medications are checked and recorded.
Check whether the test subject is wearing a smart band. (Limited to those who agree to wear the smart band.)
After the intervention procedure, MDS-UPDRS, IPAQ, Parkinson's self-test questionnaire, and PDSS tests are performed.
Conduct cost measurement and utility measurement (EQ-5D, EQ-VAS).
Check compliance up to the visit.
Specify the next visit date (however, it is calculated from the planned visit date).

11.2.3.4 방문 9~15 (5~8주)
Visits 9-15 (weeks 5-8)

시험대상자의 병력이나 치료력의 변화여부를 확인한다.
병용약물 변화여부를 확인한다.
활력징후를 측정한다.
이상반응을 확인한다.
시험대상자의 병력이나 치료약물의 변화여부를 확인하여 기록한다.
시험대상자의 스마트밴드 착용여부를 확인한다. (스마트밴드 착용에 동의한 대상자에 한함.)
시험군은 MARS-PD 치료를 실시한다.
해당 방문까지의 순응도를 확인한다.
다음 방문일을 지정한다(단, 계획된 방문일로부터 계산한다).
Check for changes in the test subject's medical history or treatment history.
Check for changes in concomitant medications.
Measure vital signs.
Check for adverse events.
Any changes in the test subject's medical history or treatment medications are checked and recorded.
Check whether the test subject is wearing a smart band. (Limited to those who agree to wear the smart band.)
The experimental group will receive MARS-PD treatment.
Check compliance up to the visit.

Specify the next visit date (however, it is calculated from the planned visit date).

11.2.3.5 방문 16 (8주)
Visit 16 (week 8)

시험대상자의 병력이나 치료력의 변화여부를 확인한다.
병용약물 변화여부를 확인한다.
활력징후, 체중을 측정한다.
이상반응을 확인한다.
시험대상자의 스마트밴드 착용여부를 확인한다. (스마트밴드 착용에 동의한 대상자에 한함.)
시험군은 MARS-PD 치료를 실시한다.
중재 시술 후 MDS-UPDRS, IPAQ, 파킨슨 자가 검사지, PDSS 검사를 실시한다.
보행검사(TUG 및 GAITRite)를 실시하며 fNIRS 검사를 수행한다.
임상검사실 검사를 시행한다.

- 혈액학적 검사: 헤모글로빈, 헤마토크릿, 적혈구수, 백혈구수, 혈소판수, HbA1c
- 혈액화학적 검사: AST, ALT, γ-GTP, BUN, creatinine
- 소변검사: SG, pH, RBC(erythrocyte), WBC(leukocyte), Nitrite, Protein, Glucose, Ketone, Urobilinogen, Bilirubin

임신진단검사로 폐경이 되지 않은 여성 대상자에 한해서 Urine HCG를 시행한다. 단, menstruation에는 방문17(대조군 방문4)에 시행한다.
비용측정 및 효용측정(EQ-5D, EQ-VAS)을 실시한다.
장내미생물 검사를 실시한다.
해당 방문까지의 순응도를 확인한다.
다음 방문일을 교육한다.
Check for changes in the test subject's medical history or treatment history.
Check for changes in concomitant medications.
Measure vital signs and weight.
Check for adverse events.
Check whether the test subject is wearing a smart band. (Limited to those who agree to wear the smart band.)
The experimental group will receive MARS-PD treatment.
After the intervention procedure, MDS-UPDRS, IPAQ, Parkinson's self-test questionnaire, and PDSS tests are performed.
Gait tests (TUG and GAITRite) are performed and fNIRS tests are performed.
Perform clinical laboratory tests.

- Hematological tests: hemoglobin, hematocrit, red blood cell count, white blood cell count, platelet count, HbA1c
- Blood chemical tests: AST, ALT, γ-GTP, BUN, creatinine
- Urine test: SG, pH, RBC (erythrocyte), WBC (leukocyte), Nitrite, Protein, Glucose, Ketone, Urobilinogen, Bilirubin

Urine HCG testing is only done on women who have not reached menopause and have passed a pregnancy test. In the case of menstruation, however, urine HCG is measured at

visit 17 (control group visit 4).
Conduct cost measurement and utility measurement (EQ-5D, EQ-VAS).
Perform gut microbiome examination.
Check compliance up to the visit.
Educate about the next visit date.

11.2.3.6 방문 17 (12주)

Visit 17 (week 12)

시험대상자의 병력이나 치료력의 변화여부를 확인한다.
병용약물 변화여부를 확인한다.
활력징후, 신장/체중을 측정한다.
이상반응을 확인한다.
시험대상자의 스마트밴드 착용여부를 확인한다. (스마트밴드 착용에 동의한 대상자에 한함.)
스마트밴드 설치에 동의한 연구 완료 대상자의 스마트밴드 측정 내역을 파일로 수집한다. (스마트밴드 착용에 동의한 대상자에 한함.)
MDS-UPDRS, IPAQ, 파킨슨 자가 검사지, PDSS 검사를 실시한다.
보행검사(TUG 및 GAITRite)를 실시하며 fNIRS 검사를 수행한다.
비용측정 및 효용측정(EQ-5D, EQ-VAS)을 실시한다.
Check for changes in the test subject's medical history or treatment history.
Check for changes in concomitant medications.
Measure vital signs and height/weight.
Check for adverse events.
Check whether the test subject is wearing a smart band. (Limited to those who agree to wear the smart band.)
Smart band measurement details of study completion subjects who agreed to smart band installation are collected in a file. (Limited to those who agree to wear the smart band.)
MDS-UPDRS, IPAQ, Parkinson's self-test questionnaire, and PDSS tests are conducted.
Gait tests (TUG and GAITRite) are performed and fNIRS tests are performed.
Conduct cost measurement and utility measurement (EQ-5D, EQ-VAS).

11.2.3.9 추가방문

Additional visit

추가방문은 예정된 방문 외에 시험대상자 요청 또는 시험자의 판단에 의해 필요하다고 판단될 때 수시로 이루어질 수 있다. 시험대상자가 예정되지 않은 날에 방문한 경우에는 이상반응, 병용약물, 시행한 검사의 결과 및 그에 따른 의학적 처치 등을 증례기록지에 기록하여야 한다. 중도 탈락된 시험대상자의 경우라도 추가방문을 실시하면 임상검사실검사, 유효성평가 검사를 시행할 수 있다.
Additional visits may be made at any time, in addition to the scheduled visit, when deemed necessary at the request of the test subject or at the discretion of the investigator. If a test subject visits on an unscheduled day, adverse events, concomitant

medications, results of tests performed, and subsequent medical treatment, etc. must be recorded in the case record. Even for test subjects who drop out, clinical laboratory tests and efficacy evaluation tests can be performed during additional visits.

12. 예측 이상반응 및 사용상의 주의사항
Predicted adverse events and precautions for use

피내침 부착에 관한 부작용으로 피하출혈(멍듦), 가벼운 통증이나 저림 등이 발생할 수 있음을 임상시험 시작 전에 대상자에게 주지시키고, 치료기간동안 방문마다 이상반응을 점검하도록 한다. 중재가 내복약이 아니기 때문에 간기능 및 신장기능과 같은 장기 손상 관련 혈액검사는 실시하지 않는다.

침 시술과 관련하여 발생 가능한 adverse event의 종류는 다음과 같다.

- 국소적이상 : 피하출혈 및 출혈반 형성, 말초신경염/봉와직염, 침에 대한 알레르기 반응, 자침부위의 통증(2주 이상 지속의 경우에 국한)
- 자율신경계 및 중추신경계 이상 : 혈압강하 및 실신/과도한 발한, 졸린 증세(1주일 이상 지속의 경우에 국한), 혼미(1일 이상 지속의 경우에 국한)/ 혼수
- 위장관 이상 : 오심/구토
- 정신적 문제 : 불안, 공포(사건 당 60시간 이상 지속의 경우에 국한), 기면
- 기타 : 두통(3일 이상 지속의 경우에 국한), 저린 증상을 동반한 과민감각(3일 이상 지속의 경우에 국한), 자침 후의 발작, 불명료 언어

상기에 언급된 예측된 이상반응 외에 새롭게 나타날 가능성을 배제할 수 없으며 본 임상시험 중 나타나는 이상반응에 대해서는 해당 기관의 연구의사가 적절한 조치를 취할 것이며 또한, 수집되는 이상반응은 대상자 또는 대상자의 법정대리인에게 즉시 정보를 제공할 예정이다.

Before beginning the clinical trial, subjects are informed that side effects from intradermal needle attachment, such as subcutaneous bleeding (bruising) and mild pain or numbness, may occur, and adverse events are monitored at each visit throughout the treatment period. Because the intervention is not administered orally, blood tests for organ damage, such as liver and kidney function, are not performed.

The following are examples of adverse events that may occur as a result of acupuncture treatment.

- Local abnormalities: subcutaneous bleeding and hemorrhagic spot formation, peripheral neuritis/cellulitis, needle allergy, pain at the needle site (cases lasting more than 2 weeks)
- Autonomic nervous system and central nervous system abnormalities: low blood pressure, syncope/excessive sweating, drowsiness (limited to cases lasting more than a week), stupor (limited to cases lasting more than a day), and coma.
- Gastrointestinal tract abnormalities: nausea/vomiting
- Mental problems: anxiety, fear (limited to cases lasting more than 60 hours per event), lethargy.
- Others: Headache (limited to cases lasting more than 3 days), hypersensitivity with numbness (limited to cases lasting more than 3 days), seizures after needling, slurred

speech.

In addition to the previously mentioned anticipated adverse events, the possibility of new occurrences cannot be ruled out, and the research doctor at the relevant institution will take appropriate measures for any adverse events that occur during this clinical trial. Furthermore, information on collected adverse events will be immediately provided to the subject or the subject's legal representative.

13. 중지 및 탈락 기준
Criteria for suspensions and dropouts

13.1 중지의 정의
definition of suspensions

부작용, 이상사례 발생 등으로 인하여 임상시험을 진행할 수 없거나 임상시험의 진행이 대상자의 안전보호를 위협하여 그 진행을 멈추는 것으로 정의한다.

It is defined as the inability to continue with a clinical trial due to side effects, adverse events, and so on, or stopping the progress of a clinical trial because it threatens subject safety.

13.2 중지의 기준
criteria for suspensions

1) 대상자의 안전과 임상시험의 진행에 심각한 영향을 줄 수 있는 이상반응이 발생한 경우
2) 기타 시험자의 판단에 의해 시험 진행이 적합하지 못하다고 판단되는 경우

임상시험 진행 중 시험대상자의 안전과 임상시험의 심각한 영향을 줄 수 있는 이상반응이 발생할 경우, 그 시점에서 진행 중인 시험대상자의 시험은 가능한 한 종료하고, 추가 시험대상자를 대상으로 한 시험 역시 일단 중지한다. 다만 임상시험계획서에 명시된 안전성 평가는 그대로 시행한다. 중재 방법의 안전성에 대해 임상시험책임자와 임상시험의뢰자가 논의하여 임상시험의 진행 또는 중단 여부를 결정하고, 결정사항에 대하여 IRB에 보고한다.

1) If an adverse event occurs that threatens the subject's safety or the progress of the clinical trial
2) Other instances where the test is deemed unsuitable at the tester's discretion

   If an adverse event occurs during a clinical trial that threatens the safety of the test subject or the clinical trial, the test for the test subject in progress will be terminated if possible, and testing for additional test subjects will be halted. The safety evaluation specified in the clinical trial protocol, on the other hand, will be carried out as planned. The clinical trial director and the clinical trial client discuss the safety of the intervention method to decide whether to continue or stop the clinical trial, and the decision is reported to the IRB.

13.3 탈락의 정의

Definition of Dropout

계획서에 명시되어 있는 대로 치료시행과 그 전후의 관찰과정을 완료한 경우 시험대상자가 시험을 완료한 것으로 정의한다. 이상반응 또는 다른 이유로 인해 시험을 완료하지 못한 경우 시험대상자가 탈락된 것으로 본다. 시험대상자의 시험탈락은 시험 기간 중 어느 시점에서나 판정 가능하며, 탈락 시에는 시험대상자에게 통지하고 시험책임자에게 보고하도록 한다.

A test subject is considered to have completed the test if he or she has followed the protocol's treatment and observation procedures before and after the test. The test subject is considered to have dropped out if he or she is unable to complete the test due to adverse events or other reasons. A test subject's failure can be determined at any time during the test period, and in the event of failure, the test subject is notified and the failure is reported to the test director.

13.4 탈락의 기준

Criteria for dropout

시험자는 치료와 관찰을 중단시키고 시험대상자를 탈락시킬 수 있으며, 시험대상자는 언제나 시험에서 자유의사로 탈락할 수 있다.

The investigator can discontinue treatment and observation and drop the test subject, and the test subject can opt out of the test at any time.

탈락의 기준은 다음과 같다.

1) 시험대상자에게 중대한 이상반응이 발생한 경우 혹은 이상반응으로 인하여 시험 진행이 어려운 경우
2) 투여 전 검사에서 발견치 못한 전신질환이 발견된 경우
3) 임상시험 기간 중 만족스럽지 못한 효과로 인하여 시험대상자 또는 시험대상자의 법정 대리인이 시험 중단을 요구하는 경우
4) 시험대상자가 시험자의 지시에 불응하는 경우
5) 시험대상자가 임상시험 참가 동의를 철회한 경우
6) 시험대상자의 추적이 안 되는 경우
7) 시험 기간 또는 경과 관찰 기간 동안 연구의사의 지시나 동의 없이 시험결과 판정에 영향을 미칠 수 있는 약물 등을 복용한 경우
8) 기타 연구의사의 판단에 의해 시험의 진행이 적합하지 못하다고 판단되는 경우

The criteria for dropout are as follows.

1) When a serious adverse event occurs in a test subject or when it is difficult to proceed with the test due to an adverse event
2) If a systemic disease that was not detected in the pre-administration examination is discovered
3) When the test subject or the test subject's legal representative requests discontinuation of the test due to unsatisfactory effects during the clinical trial period
4) If the test subject does not comply with the investigator’s instructions
5) When a test subject withdraws consent to participate in a clinical trial

6) When the test subject cannot be traced
7) If you take drugs that may affect the test results without the research doctor's instructions or consent during the test period or observation period
8) In other cases where the research doctor determines that it is not appropriate to proceed with the test

13.5 순응도와 임상시험 계획서 위반에 대한 처리 Compliance and handling of trial protocol violations

임상시험 책임자와 담당자는 임상시험 계획서의 위반이 발생하지 않도록 계획서에 대해 충분히 숙지하고 철저히 이행한다. 시험담당자는 대상자의 방문, 검사 일정 준수를 위하여 적절한 조치, 예를 들어 휴대폰 문자메시지, 전화 모니터링, 서면 통보, 이메일 등을 실시한다. 부득이하게 발생된 계획서 위반사항에 대해서는 다음에 따라 처리한다.
To avoid any violations of the clinical trial protocol, the clinical trial director and person in charge must be fully familiar with it and thoroughly implement it. The test person employs appropriate measures, such as text messaging on a mobile phone, phone monitoring, written notification, and e-mail, to ensure that the subject visits and adheres to the test schedule. Any unavoidable plan violations will be dealt with as follows.

중대한 임상시험계획서 위반의 경우 예를 들어 선정/제외기준 위반, 동의서 미취득, 임상시험 결과에 영향을 미칠 수 있는 약물 등을 복용한 경우 등 시험대상자의 안전과 시험결과에 중대한 영향을 미칠 수 있는 위반의 경우, 해당 시험대상자의 정보는 분석에서 제외함을 원칙으로 한다.
Significant violations of the clinical trial protocol (e.g., failure to obtain consent, taking drugs that may affect clinical trial results, etc.), as well as those that may have a significant impact on the safety of test subjects and test results — in the case of such a violation, the test subject's information is, in principle, excluded from the analysis.

기타 경미한 위반사항은 위반 또는 지연 정도와 사유를 정확히 기재하여 분석할 때 임상시험 결과에 영향을 주었는지 평가한다.
Other minor violations must be accurately described, as well as the reason for the violation or delay, and whether they affected the clinical trial results when analyzed.

13.6 중지 및 탈락의 처리 과정 Process of suspensions and dropouts

시험대상자가 탈락 및 중지한 날짜와 시간, 사유를 종료보고서에 기록한다. 어떠한 사유든지 탈락 및 중지한 시험대상자를 추적 관찰하기 위한 모든 노력을 기울여야 하며 안전성에 대한 추적관찰 평가를 탈락 및 중지 후 1주일 이내에 수행하여야 한다. 시험대상자는 어떠한 사유든지 언제나 시험에서 자유의사로 탈락할 수 있으며 탈락 사유에 대하여 진술할 의무는 없다. 그러나 시험자는 탈락 및 중지한 사유 또는 사유 파악의 실패 등을 종료보고서에 기록하여야 하

며 탈락한 시험대상자를 추적 관찰하는 데 모든 노력을 기울여야 한다. 시험자는 시험대상자를 시험에서 제외시킬 수 있는 선택권 또한 가지고 있다. 중지 및 탈락한 시험대상자는 새로운 시험대상자로 대체되지 않는다.
임상시험 참여 중 발생한 부작용 등의 사유로 인해 연구 참여를 중단하는 경우에는 예측하지 못한 추가 비용의 발생이 가능하므로, 해당 비용 발생 항목 및 금액에 대해 수집하기 위하여 경제성평가 증례기록 설문을 시행할 수 있다.
The date, time, and reason for the test subject's withdrawal or discontinuation are recorded in the termination report. Test subjects are free to discontinue at any time for any reason and are not required to explain their decision. The investigator must, however, record the reason for dropping out or stopping, or failure to identify the reason, in the termination report, and must make every effort to follow up on the dropped test subjects. The investigator has the option of excluding a subject from the test. Subjects who quit or withdraw will not be replaced by new ones.
If a subject discontinues participation in the study due to reasons such as side effects that occurred while participating in the clinical trial, additional unexpected costs may occur. An economic evaluation case record survey can be conducted to collect relevant cost items and amounts.

13.7 임상시험의 조기 종결
Early termination of clinical trials

시험 기간 중에 시험자, 모니터요원, 해당 정부기관에서 본 임상시험을 중지하여야 하는 상황이 발생했을 때 임상시험 책임자는 본 시험을 조기 종결할 수 있다.
If a situation arises during the trial period in which the investigator, monitor, or relevant government agency must stop the clinical trial, the clinical trial director may terminate the trial early.

14. 평가기준, 평가방법 및 통계분석방법
Evaluation criteria, evaluation methods and statistical analysis methods

14.1. 평가기준
Evaluation criteria

14.1.1 일차 유효성 평가변수
Primary efficacy endpoint

MDS-UPDRS Part III: 시험 시작 전(baseline)과 8주 후 MDS-UPDRS Part III 점수
MDS-UPDRS Part III: MDS-UPDRS Part III scores before testing (baseline) and after 8 weeks

14.1.2 이차 유효성 평가변수

Secondary efficacy endpoints

1) MDS-UPDRS Part III: 시험 시작 전(baseline)과 4주, 12주 후 MDS-UPDRS Part III 점수의 변화
2) MDS-UPDRS: 시험 시작 전(baseline)과 4주, 8주, 12주 후 MDS-UPDRS 점수의 변화
3) IPAQ: 시험 시작 전(baseline)과 4주, 8주, 12주 후 IPAQ 점수의 변화
4) 파킨슨 자가 검사지: 시험 시작 전(baseline)과 4주, 8주, 12주 후 파킨슨 자가 검사지 점수의 변화
5) PDSS: 시험 시작 전(baseline)과 4주, 8주, 12주 후 PDSS 점수 총점의 변화
6) TUG: 시험 시작 전(baseline)과 8주, 12주 후 TUG 점수의 변화
7) GAITRite: 시험 시작 전(baseline)과 8주 12주 후 GAITRite 측정값의 변화
8) fNIRS: 시험 시작 전(baseline)과 8주, 12주 후 fNIRS 측정값의 변화
9) 스마트밴드: 시험 시작 전(baseline)과 4주, 8주, 12주 갤럭시 핏2를 이용하여 측정한 걸음수, 수면시간, 수면패턴 등의 변화
10) 홍채검사 1 & 2: 홍채검사를 통한 홍채의 모양과 구조
11) 장내미생물 (baseline)과 8주 후 미생물별 비율 변화량

1) MDS-UPDRS Part III: Change in MDS-UPDRS Part III score before starting the test (baseline) and after 4 and 12 weeks
2) MDS-UPDRS: Change in MDS-UPDRS score before starting the test (baseline) and after 4, 8, and 12 weeks.
3) IPAQ: Change in IPAQ score before starting the test (baseline) and after 4, 8, and 12 weeks.
4) PSQ: Change in PSQ score before starting the test (baseline) and after 4, 8, and 12 weeks.
5) PDSS: Change in total PDSS score before starting the test (baseline) and after 4, 8, and 12 weeks.
6) TUG: Change in TUG score before starting the test (baseline) and after 8 and 12 weeks
7) GAITRite: Changes in GAITRite measurements before starting the test (baseline) and after 8 and 12 weeks
8) fNIRS: Changes in fNIRS measurements before starting the test (baseline) and after 8 and 12 weeks.
9) Smart Band**: Changes in step count, sleep time, sleep pattern, etc. measured using Galaxy Fit 2 before the start of the test (baseline) and at 4, 8, and 12 weeks.
10) Iris examination 1 & 2: Shape and structure of the iris through iris examination
11) Gut microbiome (baseline) and percentage change by microorganism after 8 weeks

14.1.3 안전성 평가변수

Safety endpoints

이상반응 평가

- 이상반응유무 및 MARS-PD와의 관련 여부를 평가

활력징후와 임상검사실 검사

활력징후와 임상검사실 검사를 통해 중재 방법의 안전성을 평가

이상반응을 포함한 안전성의 평가 기준 및 평가방법

① 부작용, 활력징후, 임상검사실 검사결과를 기록

② 안전성 평가 대상: 시험에 참여한 전례 (ITT군)에 대하여 평가

③ 임상시험 시작 전에 관찰되지 않은 증상이 발생하는 모든 바람직하지 않은 의학적 소견을 이상반응으로 분류

시험 기간 중 발생할 수 있는 이상 증상에 대한 대비

- 임상연구 시작 전에 관찰되지 않은 증상이 발생하면 이에 대한 의학적 조치를 제시

adverse event evaluation

- Evaluate the presence of adverse events and whether they are related to MARS-PD

Vital signs and laboratory tests

Assess the safety of interventions using vital signs and laboratory tests

Safety evaluation criteria and evaluation methods, including adverse events

① Record side effects, vital signs, and clinical laboratory test results

② Safety evaluation target: Evaluation of precedents (ITT group) that participated in the test

③ All undesirable medical findings that cause symptoms not observed before the start of the clinical trial are classified as adverse events.

Prepare for abnormal symptoms that may occur during the test period

- If symptoms that were not observed before the start of clinical research occur, medical measures are suggested.

14.1.4 경제성 평가변수
Economic evaluation variables

주 경제성평가지표(Primary economic endpoint): cost per QALY(Quality Adjusted Life Years) gained

- QALY: QALY 산출을 위한 삶의 질 추정은 EQ-5D로 도출된 삶의 질을 주 평가변수로 사용한다. (Area under the curve method)
- cost: 임상시험과 관련하여 발생하는 치료비용은 치료 횟수와 단위비용을 결합하여 산출하며, 임상시험 기관 내에서 발생하는 치료비용은 맹검 해제 후 연구자가 전산 자료를 조사하여 기록한다.

이차평가지표: Cost per EQ-VAS

Primary economic endpoint: cost per QALY(Quality Adjusted Life Years) gained

- QALY: For calculating QALY, quality of life derived from EQ-5D is used as the main evaluation variable (area under the curve method).
- Cost: Treatment costs associated with clinical trials are calculated by combining the number of treatments and unit costs, and treatment costs incurred within the clinical trial institution are recorded by researchers examining computerized data after blinding has been removed.

Secondary evaluation indicator: Cost per EQ-VAS

14.2 통계분석의 일반적 원칙
General principles of statistical analysis

14.2.1 분석군
analysis group

1) 전체 분석군(Full Analysis Set, FAS)
ITT 분석 원칙에 가능한 부합하게 분석 대상군을 정한다. 본 시험에서는 아래의 제외기준에 해당하는 대상자를 제외한 모든 대상자에 대하여 무작위 배정된 대로 시험결과를 분석한다. 다음에 해당하는 시험대상자는 FAS 분석에서 제외한다.
- 주요 선정기준을 만족하지 못한 경우
- 임상시험계획서에 명시된 중재를 한 번도 받지 않은 경우
- 무작위 배정 이후 한 번도 평가되지 않아 어떠한 자료도 수집하지 못한 경우

1) Full Analysis Set (FAS)
Determine the analysis target group by the ITT analysis principles. The test results in this trial are analyzed as randomly assigned to all subjects except those who meet the exclusion criteria listed below. The FAS analysis excludes the following test subjects.
- When major selection criteria are not met
- If you have never received the intervention specified in the clinical trial protocol
- If no data were collected because it was never evaluated after random assignment.

2) 계획서 순응 분석군(Per-Protocol, PP)
계획서에 명시되어 있는 대로 시험 시작 후 임상시험 계획서 위배 없이 시험의 전 과정을 완료한 시험대상자를 대상으로 분석하며, 다음에 해당하는 시험대상자는 PP분석에서 제외한다.
- 중재 기간 동안 중도 탈락한 대상자
- 선정/제외 기준을 위반한 대상자
- 전체 중재 시술 순응도가 75% 미만인 대상자
- 그 외 중대한 임상시험계획서 위반으로 간주할 수 있는 경우

2) Protocol compliance analysis group (Per-Protocol, PP)
The analysis is performed on test subjects who have completed the entire process of the test without violating the clinical trial protocol after starting the test, as specified in the protocol, and the following test subjects are excluded from the PP analysis.
- Subjects who dropped out during the intervention period
- Subjects who violated selection/exclusion criteria
- Subjects whose compliance with the overall intervention procedure is less than 75%
- In other cases that can be considered a serious violation of the clinical trial protocol

14.2.2 자료분석의 일반적 원칙
General principles of data analysis

중재방법의 유효성은 1차 평가변수의 FAS 분석 결과에 근거하여 평가하며, PP 분석군의 결과는 보조적으로 활용한다. 결측이 발생한 경우 결측량과 기전에 대한 진단을 먼저 시행한 후 적

합한 imputation 방법을 선정하여 처리한다. 이 방법은 파킨슨병의 질환특성(일상적인 상태에서는 시간에 경과함에 따른 악화되는 경향)을 고려할 때 시험결과를 보수적으로 평가할 것으로 기대된다. 통계적 검정은 양측검정을 원칙으로 하며 유의수준은 5%로 한다.

The effectiveness of the intervention method is assessed using the FAS analysis results of the primary endpoint, with the PP analysis group results serving as an auxiliary. In the case of missing data, a diagnosis of the missing amount and mechanism is performed first, followed by the selection and processing of an appropriate imputation method. This method is expected to conservatively evaluate test results while taking into account Parkinson's disease disease characteristics (the tendency to worsen over time under normal conditions). The statistical test is a two-sided test with a significance level of 5%.

14.3 인구학통계학적 자료와 시험 시작 시 임상병력 자료의 분석 Analysis of demographic data and clinical history data at trial start

본 임상시험에 포함된 시험대상자의 인구통계학적 자료 및 시험 시작 시 임상병력 자료(이하 기초자료)들은 각 시험군 별로 연속형 자료는 평균(mean), 표준편차(SD) 등을 제시하고 범주형 자료는 빈도(frequency), 백분율(percentile)을 제시한다. 처리군 별로 인구통계학적 자료와 기초자료의 비교 평가방법은 연속형 변수의 경우, 정규성 여부에 따라 Student`s independent sample t-test 또는 Wilcoxon rank sum test를 시행하고 범주형 변수의 경우, Pearson chi-squared test 또는 Fisher's exact test를 시행한다.

The following are the demographic and clinical history data (hereinafter referred to as basic data) of the subjects included in this clinical trial. Continuous data for each test group shows mean and standard deviation (SD), while categorical data shows frequency and percentage. The following is the comparative evaluation method for demographic and basic data for each treatment group. Depending on normality, the Student's independent sample t-test or Wilcoxon rank sum test is used for continuous variables, and the Pearson chi-squared test or Fisher's exact test is used for categorical variables.

14.4 유효성 평가변수의 분석방법 Analysis method of efficacy endpoints

14.4.1 일차 유효성 평가변수의 분석 Analysis of primary efficacy endpoints

1) MDS-UPDRS Part III: 시험 시작 전(baseline)과 8주 후 MDS-UPDRS Part III 점수 변화
시험군과 대조군 간의 평균 차이의 추정값과 95% 신뢰구간, 그리고 p-value 값을 제시한다.
무작위 배정을 받은 시험대상자 중 적어도 한 번 이상 중재를 받고 시험 전과 중재 시술 이후 적어도 한 번 이상 MDS-UPDRS Part III 점수가 측정된 대상자의 MDS-UPDRS Part III 총점을 분석한다. MDS-UPDRS Part III 점수의 결측이 발생한 경우, 결측량과 기전에 대한 진단을 먼저 시행한 후 적합한 imputation 방법을 선정하여 FAS 분석을 시행한다.
MDS-UPDRS Part III 점수의 변화에 대한 두 군의 차이를 independent t test를 통해 검증한다.

기저 값의 유의한 차이가 있는 경우는 이를 보정한 공분산 분석을 시행하고, 기타 기저 변수의 유의한 차이를 보정하기 위해서는 다중회귀분석을 시행한다.

각 시점별 MDS-UPDRS Part III 점수에 대한 반복 측정된 값을 대상으로 반복측정분산분석을 통해 시간과 치료간의 교호작용을 검정한다.

1) MDS-UPDRS Part III: Change in MDS-UPDRS Part III score before starting the test (baseline) and after 8 weeks

   The estimated mean difference between the experimental and control groups, 95% confidence interval, and p-value are shown. The MDS-UPDRS Part III total score of subjects who received the intervention at least once and had their MDS-UPDRS Part III score measured at least once before and after the intervention was analyzed among randomly assigned subjects. If an MDS-UPDRS Part III score is missing, the missing amount and mechanism are first determined, followed by an appropriate imputation method and FAS analysis.

   An independent t-test is used to confirm the difference in MDS-UPDRS Part III score change between the two groups. Covariance analysis is used to correct for significant differences in baseline values, and multiple regression analysis is used to correct for significant differences in other baseline variables.

   The interaction between time and treatment is investigated using repeated measures analysis of variance on MDS-UPDRS Part III scores at each time point.

14.4.2 이차 유효성 평가변수의 분석
Analysis of secondary efficacy endpoints

1) MDS-UPDRS Part III: 시험 시작 전(baseline)과 4주, 12주 후 MDS-UPDRS Part III 점수의 변화
2) MDS-UPDRS: 시험 시작 전(baseline)과 4주, 8주, 12주 후 MDS-UPDRS 점수의 변화
3) IPAQ: 시험 시작 전(baseline)과 4주, 8주, 12주 후 IPAQ 점수의 변화
4) 파킨슨 자가 검사지: 시험 시작 전(baseline)과 4주, 8주, 12주 후 파킨슨 자가 검사지 점수의 변화
5) PDSS: 시험 시작 전(baseline)과 4주, 8주, 12주 후 PDSS 점수 총점의 변화
6) TUG: 시험 시작 전(baseline)과 4주, 8주, 12주 후 TUG 점수의 변화
7) GAITRite: 시험 시작 전(baseline)과 8주 12주 후 GAITRite 측정값의 변화
8) fNIRS: 시험 시작 전(baseline)과 8주, 12주 후 fNIRS 측정값의 변화
9) 스마트밴드: 시험 시작 전(baseline)과 4주, 8주, 12주 갤럭시 핏2를 이용하여 측정한 걸음수, 수면시간, 수면패턴 등의 변화
10) 홍채검사 1 & 2: 홍채검사를 통한 홍채의 모양과 구조

* MDS-UPDRS, IPAQ, 파킨슨 자가 검사지, PDSS, TUG, GAITRite, fNIRS 검사는 공분산분석, 혹은 기저조사의 변수를 보정한 다중회귀분석을 사용하여 분석한다. 이 때, 시험 시작 전 점수가 측정된 경우는 이를 공변량으로 하며, 결측치에 대한 별도의 처리는 하지 않는 per-protocol 분석을 시행한다.

11) 장내미생물 (baseline)과 8주 후 미생물별 비율 변화량

* 정규성 검정을 시행하여 정규성을 따르는 경우 paired t-test를, 정규성을 따르지 않는 경우 Wilcoxon singed-rank test를 시행한다.

1) MDS-UPDRS Part III: Change in MDS-UPDRS Part III score before starting the test (baseline) and after 4 and 12 weeks.
2) MDS-UPDRS: Change in MDS-UPDRS score before starting the test (baseline) and after 4, 8, and 12 weeks.
3) IPAQ: Change in IPAQ score before starting the test (baseline) and after 4, 8, and 12 weeks.
4) PSQ: Changes in PSQ scores before starting the test (baseline) and after 4, 8, and 12 weeks
5) PDSS: Change in total PDSS score before starting the test (baseline) and after 4, 8, and 12 weeks.
6) TUG: Change in TUG score before starting the test (baseline) and after 4, 8, and 12 weeks.
7) GAITRite: Changes in GAITRite measurements before starting the test (baseline) and after 8 weeks and 12 weeks.
8) fNIRS: Changes in fNIRS measurements before starting the test (baseline) and after 8 and 12 weeks.
9) Smart band: Changes in step count, sleep time, sleep pattern, etc. measured using Galaxy Fit 2 before starting the test (baseline) and at 4, 8, and 12 weeks.
10) Iris examination 1 & 2: Shape and structure of the iris through iris examination

* Analysis of covariance or multiple regression analysis is used to adjust for baseline survey variables in the MDS-UPDRS, IPAQ, Parkinson's self-test, PDSS, TUG, GAITRite, and fNIRS tests. If the score is measured before the start of the test, it is used as a covariate, and per-protocol analysis is performed without treating missing values separately.

11) Gut microbiome (baseline) and percentage change by microorganism after 8 weeks

* A normality test is executed: if normality is observed, a paired t-test is carried out; if normality is not observed, a Wilcoxon signed-rank test is carried out.

14.4.3 경제성 평가 분석
Economic evaluation analysis

경제성평가분석은 FAS 분석을 원칙으로 하며 결측 분석 결과에 대한 민감도를 확인하기 위한 PP 분석을 같이 시행한다. 경제성평가의 비용 및 효과(Utility) 추정은 임상시험의 효과추정과 동일한 방식으로 missing data에 대한 기전 등을 분석한 후 적합한 imputation 방식을 선택하여 시행한다.

FAS analysis is used for economic evaluation, and PP analysis is used to test sensitivity to missing analysis results. Economic evaluation cost and utility estimation is performed by analyzing the mechanism for missing data in the same way that clinical trial effectiveness estimation is performed, and then selecting an appropriate imputation method.

임상시험과 동시에 진행하는 경제성평가는 임상연구 대상 치료와 기존치료(Usual care)간의 비용효과성을 확인하고자 시행한다. 주 경제성평가지표(Primary economic endpoint)는 Quality Adjusted Life Years(cost per QALY) gained이고, 이차평가지표는 cost per EQ-VAS 등 효과성 평가지표들이다.

Economic evaluation, which is carried out concurrently with clinical trials, is carried out to confirm the cost-effectiveness of the clinical study's treatment versus existing treatment (usual care). The primary economic endpoint is the cost per QALY gained, and the secondary endpoints are effectiveness evaluation indicators such as cost per EQ-VAS.

1차 분석기간은 18주(총 추적 기간)로 시행하며, 이후의 기간에 대한 추정이 필요할 경우 추적 기간 이후의 비용과 효과를 회귀모형 등을 통하여 외삽(extrapolation)하여 추정하거나 decision modeling analysis 등을 시행하는 2차 분석을 시행한다.
The first analysis period (total follow-up period) will be 18 weeks. If an estimate for the longer period is required, the costs and effects after the follow-up period are extrapolated and estimated using a regression model, for example, or a secondary analysis such as decision modeling analysis is performed.

임상시험과 관련하여 발생하는 치료비용은 치료 횟수와 단위비용을 결합하여 산출하며, 임상시험 기관 내에서 발생하는 치료비용은 맹검 해제 후 연구자가 전산 자료를 조사하여 기록한다. QALY 산출을 위한 삶의 질 추정은 EQ-5D로 도출된 삶의 질을 주 평가변수로 사용하고, 산출 방식은 Area under the curve method(Brazier J, Ratcliffe J, Salomon JA, Tsuchiya A: Measuring and Valuing Health Benefits for Economic Evaluation. Oxford, UK: Oxford University Press; 2007)를 이용한다.
Treatment costs associated with clinical trials are calculated by combining the number of treatments and unit costs, and treatment costs incurred within the clinical trial institution are recorded by researchers examining computerized data after blinding has been removed. For calculating QALY, the quality of life derived from EQ-5D is used as the main evaluation variable, and the area under the curve method is used as the calculation method (Brazier J, Ratcliffe J, Salomon JA, Tsuchiya A: Measuring and Valuing Health Benefits for Economic Evaluation. Oxford, UK: Oxford University Press; 2007).

총 분석 기간(Time Horizon)이 12개월 이상이 될 경우 비용단위는 2021년 대한민국 화폐단위(원)으로 통일하고, 건강보험심사평가원 경제성평가 지침에 근거하여 5%의 할인율을 적용한다.
If the total analysis period (time horizon) exceeds 12 months, the cost unit is converted to Korean won in 2021, and a 5% discount rate is applied based on the Health Insurance Review and Assessment Service economic evaluation guidelines.

본 연구의 분석관점은 사회적 관점이며, Baseline analysis에서는 연구에서 사용되는 모수들(parameter)의 대표값(평균 등)을 사용하여 시행하고, 민감도분석은 가능한 모든 추정모수들의 분포와 대표값 들을 사용하여 확률적 민감도 분석(Probabilistic Sensitivity Analysis)을 시행한다.
This study's analytical perspective is a social perspective. Baseline analysis is carried out using representative values (average, etc.) of the study's parameters. The distribution and representative values of all possible estimated parameters are used in the sensitivity analysis.

분석 결과의 제시는 Incremental cost effectiveness ratio를 포함한 결과 분석 테이블들과 함께, 비모수적 방법으로 확인된 신뢰구간을 포함하는 Cost Effectiveness plane과, National threshold 의 변화에 따른 비용효과성의 민감도를 확인할 수 있는 Cost Effectiveness Acceptability Curve(CEAC), 대상 인구집단의 정보의 가치를 추정할 수 있는 Value of information analysis 결과 그래프 등을 함께 제시한다.
The results of the analysis are presented along with result analysis tables that include the incremental cost effectiveness ratio. There is also a Cost Effectiveness plane with a confidence interval confirmed by a non-parametric method, a Cost Effectiveness Acceptability Curve (CEAC) that can check the sensitivity of cost effectiveness to changes in the national threshold, Value of information analysis results that can be used to estimate the value of information on the target population group, and so on.

본 연구의 통계분석은 표본 수가 30 이하인 경우 모든 연속형 변수들의 정규성을 Shapiro-Wilk test 등을 통해 먼저 검증한 후 시행하며, 통계적 유의수준은 p-value 0.05로 검정하고, 분석 프로그램은 Stata 14 MP version과 R program(4.0.2 version)을 사용하여 시행하며, 모델링을 통한 외삽을 수행한 경우에는 Treeage Pro 2016을 사용하여 연구를 수행한다.
The statistical analysis of this study is performed after first verifying the normality of all continuous variables through the Shapiro-Wilk test when the sample number is 30 or less. The statistical significance level is tested at p-value 0.05, and the analysis program is performed using Stata 14 MP version and R program (4.0.2 version). When extrapolation through modeling is performed, the study is conducted using Treeage Pro 2016.

14.5 안전성 변수의 분석방법
Analysis method of safety variables

임상검사실 검사의 결과치에 대해 시험 전과 비교하여 임상적으로 어떤 변화가 있었는지를 평가한다. 발현된 모든 이상반응은 자세한 설명과 함께 나열한다. 중대한 이상반응은 모두 서술적으로 기술된다. 이상반응은 환자의 증상 보고, 연구자의 관찰 등을 통해 수집된다. 시험약과 연관성이 있는 이상반응과 연관성이 없는 이상반응의 빈도를 기록하며, 기술통계량으로 제시한다.
이상반응의 빈도, 발현율, 각각의 목록, 상세한 발현시간, 심각한 정도 및 시험약물과의 인과관계 등을 제시하며, 필요한 경우 그래프 형태로 보고한다. 통계적 분석이 필요한 경우는 변수의 특성과 목적에 따라 paired t-test, Mcnemar test, ANOVA, t-test, chi-square test 혹은 Fisher's exact test 등을 실시한다.
Examine whether there have been any clinical changes in the results of clinical laboratory tests since the test. All adverse events are documented and explained in detail. All severe adverse events are described in detail. Patients' symptom reports and researchers' observations are used to collect adverse events. The frequency of adverse events associated with and unassociated with the test drug is documented and presented as descriptive statistics.

The frequency, occurrence rate, list of each adverse event, detailed onset time, severity, and causal relationship with the test drug are presented and, if required, graphically reported. When statistical analysis is required, the paired t-test, Mcnemar test, ANOVA, t-test, chi-square test, or Fisher's exact test are used, depending on the variable's characteristics and purpose.

15. 이상반응
Adverse events

15.1 안전성 관련 용어의 정의
Definition of safety-related terms

1) 이상반응(Adverse Event/Adverse Experience, AE)
임상시험 중재를 받은 시험대상자에게 발생한 모든 유해하고 의도하지 않은 증후(sign), 증상(symptom) 또는 질병을 말하며, 해당 중재방법과 반드시 인과관계를 가져야 하는 것은 아니다.
It refers to any harmful and unintended sign, symptom, or disease that occurs in a test subject who has received a clinical trial intervention and is not necessarily caused by the relevant intervention method.

2) 이상약물반응(Adverse Drug Reaction, ADR)
임상시험용 의약품의 임의 용량에서 발생한 모든 유해하고 의도하지 않은 반응으로서 임상시험용 의약품과의 인과관계를 부정할 수 없는 경우를 말한다. 본 임상시험에서는 의약품이 중재가 아니기 때문에 해당사항이 없다.
This refers to any unintended and harmful reaction that occurs at any dose of a clinical investigational drug and where the causal relationship with the clinical investigational drug cannot be denied. Because the drug is not an intervention, this is not applicable in this clinical trial.

3) 중대한 이상반응/이상약물반응(Serious AE/SAE)
임상시험 중재로 인하여 발생한 이상반응 또는 이상약물반응 중에서 다음의 어느 하나에 해당하는 경우를 말한다.
① 사망하거나 생명에 대한 위험이 발생한 경우
② 입원할 필요가 있거나 입원 기간을 연장할 필요가 있는 경우
③ 영구적이거나 중대한 장애 및 기능 저하를 가져온 경우
④ 태아에게 기형 또는 이상이 발생한 경우
This refers to any of the following cases among adverse events or adverse drug reactions occurring due to clinical trial intervention.
① In case of death or danger to life
② When there is a need to be hospitalized or to extend the hospitalization period
③ In case of permanent or serious disability or decline in function
④ If a deformity or abnormality occurs in the fetus

4) 예상하지 못한 이상약물반응(Unexpected ADR)
   임상시험자 자료집 또는 의약품의 첨부 문서 등 이용 가능한 의약품 관련 정보에 비추어 이상약물반응의 양상이나 위해의 정도에서 차이가 나는 것을 말한다. 본 임상시험에서는 의약품이 중재가 아니기 때문에 해당사항이 없다.
   This refers to differences in the pattern of adverse drug reactions or the degree of harm based on drug-related information available, such as the clinical investigator data book or drug product attachment documents. Because the drug is not an intervention, this is not applicable in this clinical trial.

15.2 이상반응의 평가 Evaluation of adverse events

15.2.1 안전성의 평가 Evaluation of safety

부작용, 활력징후, 임상검사실 검사결과를 기록한다.
본 임상시험의 중재를 1회 이상 받은 모든 시험대상자를 안전성 평가 대상으로 한다.
Keep track of any side effects, vital signs, and lab test results.
All subjects who received the intervention of this clinical trial at least once are being evaluated for safety.

15.2.2 이상반응의 평가 원칙 Principles for evaluating adverse events

- 이상반응은 중재를 받기 전에 관찰되지 않은 증상이 중재 기간 중에 새로 나타난 증상으로서 중재 방법과의 인과관계에 상관없이 의도하지 않았던 증후(임상검사실 검사상 비정상치 포함) 및 증상과 약물 사용과 관련된 일시적인 현상 등을 총칭한다.
  중재 방법의 이상반응으로 예상되는 현상(증상, 시작일, 지속기간 등)은 이상반응 보고서에 빠짐없이 기록하도록 한다.
  이상반응 정도에 대한 평가는 시험담당자가 평가 기준을 참고하여 증상의 경중에 따라 단계별로 평가하는 것을 원칙으로 한다.
  중재 방법과의 인과관계는 시험담당자가 평가 기준에 따라 6단계로 분류하여 평가한다.
- Adverse events are symptoms that were not present prior to receiving intervention but appear during the intervention period. It refers to unintended symptoms (including abnormal clinical laboratory test results) and temporary phenomena associated with symptoms and drug use, regardless of the causal relationship with the intervention method.
  All anticipated adverse events associated with the intervention method (symptoms, start date, duration, and so on) must be fully documented in the adverse event report.
  In general, the tester evaluates the severity of adverse events in stages based on the severity of the symptoms, referring to the evaluation criteria.

The test staff evaluates the causal relationship with the intervention method by categorizing it into six levels based on the evaluation criteria.

15.2.3 중증도(Severity) 평가
Severity assessment

이상반응의 중증도는 최대강도(maximum intensity)에 의거하여 아래의 기준에 의하여 분류한다. 아래의 기준에 의하여 분류가 어려운 경우에 CTCAE Version 4.0을 참고할 수 있다.

The severity of adverse events is classified using the following criteria based on maximum intensity. CTCAE Version 4.0 can be used if classification is difficult based on the criteria listed below.

1 = 경증<br>(Mild)	시험대상자가 거의 느끼지 못할 정도로 정상적인 일상생활(기능)을 저해하지 않는 정도, 대부분 치료가 필요하지 않은 정도<br>A degree that does not interfere with normal daily life (function) to the extent that the test subject barely notices it and, in most cases, does not necessitate treatment.
2 = 중등증<br>(Moderate)	시험대상자가 불편감을 느낄 수 있으며, 정상적인 일상적인(기능)을 저해하는 정도, 시험대상자가 시험을 계속할 수는 있으나 치료가 필요할 수도 있는 경우<br>The test subject may experience discomfort, to the extent that normal daily life (functioning) is hampered, and the test subject may be able to continue the test but may require treatment.
3 = 중증<br>(Severe)	시험대상자가 매우 불편하여 일상생활(기능)이 불가능하고, 시험의 지속적인 참여가 불가능한 정도, 치료나 입원이 필요할 수 있는 정도<br>The extent to which the test subject is so uncomfortably uncomfortable that daily life (function) is impossible, continuous participation in the test is impossible, and treatment or hospitalization may be required.

15.2.4 시험치료법과의 인과관계 평가
Evaluation of causal relationship with test treatment

임상시험 중재 방법과의 인과관계는 통계학적으로 검정할 수 없으므로 개인적 상황, 의학적(생리학적, 병리학적, 약리학적) 가능성, 문헌 등을 이용하여 판단하며, 시간적 관계를 고려하는 것도 도움이 된다. 또한, 처치 중지 및 재처치에 의한 소실, 재발현 여부, 병용요법 및 복용 약제 등을 고려한다.

이상반응과 임상시험 중재 방법과의 관계(또는 기타 원인 - 기저 질환의 진행, 병용 치료 등)의 확실성의 정도는 아래의 관점에서 이상반응이 얼마나 잘 설명될 수 있는가에 따라 결정한다.

- 임상시험 중재 방법에 대해 알려진 효과 및 기전
- 임상시험용 중재 방법과 유사한 치료법에서 이전에 관찰되었던 유사한 작용
- 유사한 중재 방법과 관련 있다고 자주 보고된 반응들
- 중재 방법 시술의 시간과 연관되어 나타나는 반응(중지 시 없어지고, 재시술 시 다시 발생)

Because the causal relationship with clinical trial intervention methods cannot be tested statistically, it is judged based on personal circumstances, medical (physiological,

pathological, pharmacological) possibilities, literature, and other factors, including temporal relationships. Furthermore, disappearance and re-occurrence due to treatment discontinuation and re-treatment, combination therapy, and medications used are taken into account.

The degree of certainty about the relationship between an adverse event and the clinical trial intervention method (or other causes such as disease progression, concomitant treatment, etc.) is determined by how well the adverse event can be explained in terms of:

- Known effects and mechanisms of clinical trial intervention methods
- Similar actions previously observed in treatments similar to the investigational intervention
- Reactions frequently reported to be associated with similar intervention methods
- Reactions that appear in relation to the time of the intervention method procedure (disappear when stopped, and occur again when the procedure is repeated)

1) 관련성이 명백함(Definitely related)
   - 중재 방법과 이상반응 발현의 시간적 순서가 타당한 경우
   - 이상반응이 다른 어떤 이유보다 중재 방법에 의해 가장 개연성이 있게 설명되는 경우
   - 중재 방법 시술 중단으로 이상반응이 사라지는 경우
   - 재치료(rechallenge, 가능한 경우에만 실시) 결과가 양성인 경우
   - 이상반응이 동일한 중재 방법에 대해 이미 알려져 있는 정보와 일관된 양상을 보이는 경우
   - When the intervention method and temporal sequence of adverse event occurrence are reasonable
   - When the adverse event is most likely explained by the intervention method rather than any other reason.
   - When adverse events disappear when the intervention method is discontinued
   - Rechallenge (performed only when possible) If the result is positive
   - If the adverse event shows a consistent pattern with information already known about the same intervention method

2) 관련성이 많음(Probably related)
   - 중재 방법과 이상반응 발현의 시간적 순서가 타당한 경우
   - 이상반응이 다른 원인보다 중재 방법에 의해 더욱 개연성이 있게 설명되는 경우
   - 중재 방법 시술 중단으로 이상반응이 사라지는 경우
   - When the intervention method and temporal sequence of adverse event occurrence are reasonable
   - When the adverse event is more likely explained by the intervention method than by other causes
   - When adverse events disappear when the intervention method is discontinued

3) 관련성이 의심됨(Possibly related)
   - 중재 방법과 이상반응 발현의 시간적 순서가 타당한 경우
   - 이상반응이 다른 가능성이 있는 원인들과 같은 수준으로 중재 방법에 기인한다고 판단되는

경우
- 중재 방법 시술 중단으로(실시된 경우) 이상반응이 사라지는 경우
- When the intervention method and temporal sequence of adverse event occurrence are reasonable
- When the adverse event is judged to be attributable to the intervention method to the same extent as other possible causes.
- When adverse events disappear by discontinuing the intervention method (if performed)

4) 관련성이 적음(Probably not related)
- 이상반응에 대해 보다 가능성 있는 원인이 있는 경우
- 중재 방법 시술 중단 결과(실시된 경우)가 음성이거나 모호한 경우
- 재치료(실시된 경우) 결과가 음성이거나 모호한 경우
- When there is a more likely cause for the adverse event
- If the results of intervention (if performed) are negative or ambiguous.
- If retreatment (if performed) results are negative or ambiguous

5) 관련성이 없음(Definitely not related)
- 시험대상자가 중재 방법을 시술받지 않은 경우
- 중재 방법과 이상반응 발현 간의 시간적 순서가 타당하지 않은 경우
- 이상반응에 대해 다른 명백한 원인이 있는 경우
- When the test subject has not received the intervention method
- When the temporal sequence between the intervention method and the occurrence of adverse events is not reasonable.
- If there is another obvious cause for the adverse event

6) 평가 불가능(Unknown)
- 정보가 불충분하거나 상충되어 판단할 수 없고 이를 보완하거나 확인할 수 없는 경우
- If the information is insufficient or conflicting, it is impossible to make a decision and it cannot be supplemented or confirmed.

15.3 이상반응 보고
adverse event reporting

15.3.1 이상반응 교육

The principal investigator shall provide training to the test staff and test subjects or guardians to explain all adverse events that may occur after the intervention method and to report any phenomena that appear after treatment.

15.3.2 중대한 이상반응의 보고
Reporting of serious adverse events

시험담당자는 시험 기간 중 발생한 모든 중대한 이상반응을 중재와의 관련성 여부와 상관없이

24시간 이내에 연구책임자에게 보고하도록 한다. 이외에도 시험담당자가 중대하다고 간주하거나, 중재와 연관 지을 수 있는 유의한 위험, 금기, 부작용, 주의사항을 시사하는 사건 등도 중대한 이상반응으로 증례기록지에 기록한다.
All serious adverse events that occurred during the study period must be reported to the principal investigator within 24 hours, regardless of whether they were related to the intervention. Furthermore, events deemed serious by the test administrator or indicating significant risks, contraindications, side effects, or precautions associated with the intervention are recorded as serious adverse events in the case record.

중대한 이상반응이 발생하는 경우에는 시험책임자는 계획서나 임상시험자 자료집 등에서 즉시 보고하지 않아도 된다고 명기한 것을 제외한 모든 중대한 이상반응을 즉시 의뢰자에게 알리고, 발생 5일 이내에 문서로 상세한 내용이 포함된 추가 보고를 실시한다. 별도의 지시가 있을 때까지 일단 임상 시험을 중지한다.
If a serious adverse event occurs, the study director must immediately notify the sponsor of all serious adverse events, with the exception of those specified in the protocol or clinical investigator data sheet as not requiring immediate reporting. Within 5 days of the occurrence, an additional report with detailed information will be made in writing. Clinical trials will be halted until further notice is provided.

의뢰자는 기타 관련된 시험자, 기관생명윤리위원회 및 식품의약품 안전처장에게 중대하고 예상하지 못한 모든 이상 반응을 다음 각 호에서 정한 기간 내에 신속히 보고한다.
Within the timeframes specified in the following items, the sponsor must promptly report all serious and unexpected adverse events to other relevant investigators, the institutional review board, and the Minister of Food and Drug Safety.

① 사망을 초래하거나 생명을 위협하는 경우에는 의뢰자가 이 사실을 보고 받거나 알게 된 날로부터 7일 이내, 다만, 이 경우 상세한 정보를 최초 보고일로부터 8일 이내로 추가로 보고한다.
② 다른 모든 중대하고 예상하지 못한 이상반응의 경우에는 의뢰자가 이 사실을 보고 받거나 알게 된 날로부터 15일 이내에 추가로 보고한다.
① In cases where death or life is threatened, the information must reported with the fact within 7 days of the client receiving or becoming aware of the fact. In this case, however, detailed information must be reported within 8 days of the initial report date.
② In the case of all other serious and unexpected adverse events, additional reports must be made within 15 days from the date the requester receives or becomes aware of this fact.
최종 보고 시에는 가능하다면 다음의 정보가 제공되어야 한다: 발생시간, 정도, 처치, 경과, 중재와의 인과관계 등에 대한 정보를 중대한 이상반응 보고서에 기록하여야 한다.
의뢰자는 식품의약품안전처장에게 이상약물반응을 보고할 경우, 이상약물반응 보고서에 임상시험책임자 또는 임상시험담당자로부터 보고 받은 내용을 첨부하여 보고한다. 또한, 해당 이상약물반응이 종결(해당 이상약물반응의 소실 또는 추적조사의 불가 등)될 때까지 추가적인 안전성

정보를 주기적으로 보고한다. 복수의 실시기관에서 임상시험을 실시하는 경우에는 해당 실시기관에 즉시 통보해야 한다. 단, 본 임상시험은 의약품이 중재 방법이 아니기 때문에 해당 없다.
In the final report, the following information should be provided, if possible: Information on occurrence time, extent, treatment, course, causal relationship with intervention, etc. should be recorded in the serious adverse event report.
The information received from the clinical trial director or person in charge of the clinical trial to the adverse drug reaction report should be included when reporting an adverse drug reaction to the Minister of Food and Drug Safety. Furthermore, additional safety information is reported regularlya regular basis until the adverse drug reaction is terminated (abnormal drug reaction disappearance, inability to follow-up, etc.). If clinical trials are being conducted at multiple implementation institutions, the appropriate implementation institutions must be notified right away. This clinical trial, however, is inapplicable because pharmaceuticals are not an intervention method.

15.3.3 이상반응 발생 시 조치사항
Measures to be taken when adverse events occur

본 시험기간 중 시험책임자, 시험담당자는 시험대상자의 안전에 만전을 기하여야 하며, 예측되지 않은 중대한 이상반응 발생 시에는 신속하고 적절한 조치를 취하여 이상반응을 최소화하여야 한다.
During the testing period, the principal investigator and staff must make every effort to ensure the safety of the test subjects, and if unexpected serious adverse events occur, prompt and appropriate measures must be taken to minimize the adverse events.

15.3.4 이상반응의 추적관찰
Follow-up of adverse events

이상반응 발생으로 인하여 조기에 시험을 중지하여야 할 경우에는 이상반응 발생보고서를 작성하고 필요한 경우, 혈액검사 등을 실시한다.
시험담당자는 이상반응이 나타난 시험대상자에 대해 증상이 소실되고 상태가 안정될 때까지 추적 관찰해야 하며, 의뢰자가 요청하는 경우 이상반응의 이후 진행 경과에 대한 보고서를 제출하여야 한다.
If the test must be halted early due to an adverse event, an adverse event occurrence report must be prepared, and a blood test, etc. must be performed if necessary.
The test staff must follow up on test subjects who develop adverse events until the symptoms disappear and the condition stabilizes, and a report on the subsequent progress of the adverse event must be submitted if requested by the client.

16. 시험자의 의무
Investigator's Obligations

16.1 임상시험기록과 근거문서

Clinical trial records and supporting documents

시험자는 모든 임상시험 관련 통신, 시험대상자의 기록, 동의서를 연구 완료 후 임상시험 실시 기관 문서보관실에 3년간 보관한다.

After the study is completed, the investigator keeps all clinical trial-related communications, test subject records, and consent forms in the clinical trial institution's archives for 3 years.

16.2 임상시험계획서의 수정

Modification of clinical trial protocol

연구책임자는 임상시험계획서의 수정이 필요한 경우 기관생명윤리위원회에 이를 알려야 한다.

If modifications to the clinical trial protocol are necessary, the principal investigator must notify the Institutional Review Board (IRB).

16.3 근거자료 공개

Disclosure of supporting data

본 임상시험의 결과로 얻어진 개별 시험대상자의 의학적 정보는 기밀 사항으로 간주되며, 다음에 명시된 경우를 제외하고 제삼자에게 공개하는 것을 금한다. 그러한 의학적 정보는 시험대상자 개인의 의사나 시험대상자의 안녕에 책임이 있는 다른 의료인에게 제공될 수 있다. 본 임상시험의 결과로 생성된 근거자료는 모니터링이나 정도 관리, 감사 또는 실태조사를 위해 임상시험수탁기관, 기관생명윤리위원회 또는 식품의약품안전처에 공개될 것이다. 전자증례기록지를 통해 수집된 데이터는 한국한의약진흥원의 접근제어, 이중보안, 데이터베이스의 암호화등의 개인정보 보호 정책에 의해 보관되어 활용되며, 최대 10년의 보유 기간이 끝난 후 파기될 것이다.

Individual test subjects' medical information obtained as a result of this clinical trial is considered confidential, and disclosure to third parties is prohibited except as specified below. Such medical information may be disclosed to the subject's physician or another health care provider who is responsible for the subject's well-being. This clinical trial's supporting data will be disclosed to the clinical trial contract organization, institutional review board, or Ministry of Food and Drug Safety for monitoring, quality control, audit, or fact-finding purposes. Data collected through the electronic case record will be stored and used in accordance with personal information protection policies of the National Institute for Korean Medicine Development (NIKOM), such as access control, double security, and database encryption, and will be destroyed after a retention period of up to 10 years.

17. 시험의 윤리적 수행과 대상자의 안전보호에 관한 대책 Measures for ethical conduct of tests and safety protection of subjects

17.1 시험의 윤리적 수행

Ethical conduct of trial

본 시험은 ICH GCP 가이드라인, 헬싱키선언(서울 2008), 한국 GCP 가이드라인, 한국 약사법, 기관생명윤리위원회(IRB), 자료 보호에 관한 규정 등 모든 적용 가능한 규정을 준수하여 수행한다.

This trial will be conducted in compliance with all applicable regulations, including the ICH GCP Guidelines, Declaration of Helsinki (Seoul 2008), Korean GCP Guidelines, Korean Pharmaceutical Affairs Act, Institutional Review Board (IRB), and data protection regulations.

17.2 시험자의 역할

Role of investigator

시험자(Investigator)는 시험책임자, 시험담당자를 말한다.

시험 개시 이전에 본 시험계획서(개정판 포함), 서면 환자동의서, 동의 양식의 개정, 환자 등록 절차(예: 광고), 환자에게 제공되는 서면 정보, GCP 요건을 준수하겠다는 서약서에 대하여 IRB의 승인을 서면으로 날짜와 함께 받는다. IRB 승인서에서 계획서의 버전과 검토된 문서들이 확인되도록 한다.

시험자는 계획서를 준수하여 임상시험을 실시하여야 한다. 임상시험 중 또는 임상시험 이후에도, 시험자는 임상적으로 의미 있는 임상병리적 검사치의 이상을 포함하여 임상시험에서 발생한 모든 이상반응에 대해 시험대상자가 적절한 의학적 처치를 받을 수 있도록 조치하여야 하고, 시험자가 알게 된 시험대상자의 병발 질환에 대해 의학적 처치가 필요한 경우 이를 시험대상자에게 알려주어야 한다. 시험자는 임상시험계획을 정확히 분석 및 숙지하고 대상 시험대상자의 문제점에 적극적으로 대응한다.

Investigator refers to the principal investigator and test staff.

Obtain IRB approval for this study protocol (including any revisions), written patient consent, revisions to the consent form, patient registration procedures (e.g., advertisements), written information provided to patients, and a pledge to comply with GCP requirements prior to the start of the study. Ensure that the IRB approval letter specifies the protocol version and the documents reviewed.

Investigators must follow the protocol when conducting clinical trials. The investigator must ensure that the test subject receives appropriate medical treatment for all adverse events that occur during the clinical trial, including clinically meaningful abnormalities in clinicopathological test values, whether during or after the clinical trial. If the investigator becomes aware that medical treatment is required for a subject's concurrent disease, the subject must be informed. Investigators accurately analyze and comprehend the clinical trial plan and actively respond to test subjects' problems.

17.3 임상시험 실시기관의 역할

Role of clinical trial institute

실시기관의 장은 각 임상시험 단계별로 해당 임상시험의 실시에 필요한 임상시험실 및 설비와 전문인력을 갖추고 해당 임상시험을 적정하게 실시할 수 있도록 준비를 철저히 해야 한다.

The head of the implementing agency must thoroughly prepare to conduct the clinical trial by equipping the clinical laboratory, equipment, and professional personnel required for the clinical trial at each stage of the trial.

17.4 임상시험 계획서의 승인 및 수정
Approval and modification of clinical trial protocol

임상시험계획서는 기관생명윤리위원회에 보고하고 승인받아야 한다. 승인 이전에 시험대상자를 임상시험에 참여시킬 수 없다.
임상시험계획서를 시험절차가 더 광범위해지거나 위험도가 높아지거나 시험대상자 선정기준에 변화가 있거나 추가적인 안전성 정보로 인해 임상시험계획서를 변경하는 경우에는 수정을 해야 한다. 임상시험계획서를 수정할 때에는 개정 일자, 개정 이유, 개정 내용 등을 기록하여 보관하여야 하며 기관생명윤리위원회에 보고해야 한다.
시험자는 시험대상자에게 발생한 즉각적 위험 요소의 제거가 필요한 경우를 제외하고는, 계획서와 다르게 임상시험을 실시하여서는 안 된다.
The protocol for the clinical trial must be reported to and approved by the Institutional Review Board (IRB). Subjects are not permitted to participate in clinical trials prior to approval.
When the trial procedure becomes more extensive, the risk increases, the subject selection criteria change, or the clinical trial protocol is changed due to new safety information, the clinical trial protocol must be revised. When revising a clinical trial protocol, the date of revision, reason for revision, revision content, and so on must be recorded, stored, and reported to the IRB.
Except in cases where immediate risk factors for test subjects must be removed, investigators should not deviate from the protocol when conducting clinical trials.

17.5 임상시험 계획서의 숙지
Accurate understanding of the clinical trial protocol

시험책임자 및 담당자들은 시험계획을 정확히 분석 및 숙지하고 임상시험 대상 시험대상자의 문제점을 적극적으로 대응한다.
The test director and staff in charge must accurately analyze and comprehend the test plan, as well as actively respond to clinical trial subjects' problems.

17.6 시험대상자 동의
Subject consent

시험대상자 동의(Patient Informed Consent)는 시험대상자가 임상시험 참여 여부를 결정하기 전(시험과 관련된 임의의 모든 절차를 시작하기 전)에 서면을 통하여 이루어진다.
의뢰자는 동의서 요건들을 충족하면서 시험대상자가 쉽게 이해할 수 있게 기술된 동의서 견본을 제공하여 기관생명윤리위원회의 승인을 얻는다.
시험대상자 본인 또는 대리인이 동의서 서식, 시험대상자 설명서 및 기타 문서화된 정보를 읽

을 수 없는 경우에는 공정한 입회자가 동의를 얻는 전 과정에 참석하여야 한다.
시험자는 시험대상자를 위한 설명서를 통해 본 시험에 참여함에 따른 이익과 위험을 포함하여 임상시험과 관련된 모든 정보를 제공하고, 서명과 서명 날짜가 포함된 문서에 서명하도록 하여 본인이 자발적으로 임상시험에 참여함을 확인하여야 한다.
시험자는 시험대상자 또는 시험대상자의 법정 대리인이 직접 서명하고 날짜를 쓴 동의서 원본을 보관하고 사본을 제공하여야 한다.
동의를 얻기 전에 시험자는 시험대상자 또는 대리인이 임상시험의 세부 사항에 대해 질문하고 해당 임상시험의 참여 여부를 결정할 수 있도록 충분한 시간과 기회를 주어야 하며, 모든 임상시험 관련 질문에 대해 시험대상자 또는 대리인이 만족할 수 있도록 대답해 주어야 한다.
동의서 양식은 시험 기간 중 시험대상자의 안전과 관련된 중요한 정보가 입수되는 경우 개정될 수 있다. 이 경우 추가 정보에 관하여 항상 IRB와 기존 시험대상자에게 알리고 동의를 다시 얻어야 하며 이는 이전과 같은 방법으로 기록된다.
시험자는 시험대상자의 동의하에 시험대상자의 주치의에게 시험대상자의 임상시험 참가를 알린다.
Patient Informed Consent is obtained in writing before the subject decides whether or not to participate in the clinical trial (and before any trial-related procedures begin).
The sponsor obtains institutional review board approval by providing a sample consent form written in a way that subjects can easily understand while meeting the consent form requirements.
If the subject or his or her representative is unable to read the consent form, subject manual, or other documented information, an impartial witness must be present throughout the consent process.
Through an instruction manual for subjects, the investigator provides all information related to the clinical trial, including the benefits and risks of participating in the trial. Furthermore, the test subject must sign a document containing his or her signature and the date of signature to confirm that he or she is voluntarily participating in the clinical trial.
The original consent form signed and dated by the test subject or the test subject's legal representative must be kept by the investigator, and a copy must be provided.
Before obtaining consent, the investigator must give the subject or representative enough time and opportunity to ask questions about the clinical trial's details and decide whether or not to participate. All clinical trial-related questions must be answered to the test subject's or representative's satisfaction.
If important information about the safety of test subjects is obtained during the study period, the consent form may be revised. In this case, the IRB and existing test subjects must always be informed of the new information, and consent must be obtained and recorded in the same manner as before.
The investigator notifies the subject's attending physician of the subject's participation in the clinical trial with the subject's consent.

17.7 정확한 시험대상자의 선정

Accurate selection of test subjects

본 임상시험에 앞서 시험대상자와의 충분한 면담 및 검사를 통하여 시험대상자 적합 여부에 대하여 철저히 평가하여야 한다.
The suitability of the test subject must be thoroughly evaluated prior to this clinical trial through sufficient interviews and examinations with the test subject.

17.8 임상시험의 진행 점검

Checking the progress of clinical trials

임상시험책임자는 의뢰자에게 주기적으로 이상반응, 시험진행, 상황, 결과 등에 대하여 보고하며 임상시험 의뢰자는 주기적으로 임상시험 진행 상황에 대하여 점검을 시행한다.
The clinical trial director reports adverse events, trial progress, situations, results, and so on to the sponsor on a regular basis, and the clinical trial sponsor monitors the clinical trial progress on a regular basis.

17.9 모니터링의 역할

The role of monitoring

모니터링(Monitoring)은 임상시험 진행 과정을 감독하고, 해당 임상시험이 계획서, 표준작업지침서, 임상시험 관리기준 및 관련 규정에 따라 실시, 기록되는지를 정기적으로 검토, 확인한다. 임상시험에 대한 모니터링은 모니터링 담당자의 정기적인 시험자 방문과 전화로 이루어지도록 한다. 방문 시 모니터는 시험대상자 기록 원본, 자료보관(연구 파일) 등을 확인한다. 또한, 모니터는 임상시험 진행 과정을 잘 살피고, 문제가 있으면 시험자와 상의한다.
Monitoring oversees the progress of the clinical trial and reviews and confirms on a regular basis whether the clinical trial is being conducted and recorded in accordance with the protocol, standard operating procedures, clinical trial management standards, and related regulations.
Clinical trials should be monitored by monitoring staff through regular visits to investigators and phone calls. The monitor checks the original test subject records, data storage (research files), and so on during the visit. Furthermore, the monitor closely monitors the clinical trial progress and consults with the investigator if any problems arise.

17.10 시험대상자 기록의 비밀보장

Confidentiality of test subject records

시험대상자의 신원을 파악할 수 있는 기록은 비밀로 보장될 것이며, 임상시험의 결과가 출판될 경우에도 시험대상자의 신원을 비밀상태로 유지한다.
본 시험에 관련된 의뢰자, 모니터 및 점검자는 본 시험의 모니터링과 점검 및 진행 사항 관리를 위한 목적으로 시험대상자의 기록을 열람할 수 있다. 시험자는 본 계획서에 서명함으로써, 국내의 법규와 윤리적 측면에서 임상시험 의뢰자 또는 임상시험수탁기관의 모니터 및 점검자가 시험대상자의 차트와 증례기록지 기록을 검증하기 위하여 해당 문서를 검토하거나 복사할

수도 있음을 인정한다. 이러한 정보들은 기밀로 보관되어야 하며, 기밀 보관을 위한 시설과 그 관리기준을 갖추고 있어야 한다.

한편, 증례기록지 등 임상시험에 관련된 모든 서류에는 시험대상자 이름이 아닌 시험대상자 식별코드(일반적으로 시험대상자 이니셜)로 기록하고 구분한다.

Records that can be used to identify the test subject will be kept private, and the test subject's identity will be kept private even after the clinical trial results are published.

Sponsors, monitors, and inspectors involved in this test can view the test subject's records in order to monitor, inspect, and manage the test's progress. By signing this protocol, the investigator agrees that, in accordance with domestic laws, regulations, and ethics, the clinical trial sponsor's or clinical trial consignment institution's monitors and inspectors may review or copy the relevant documents to verify the subject's chart and case record. This information must be kept confidential, and there must be facilities and management standards in place for confidential storage.

Meanwhile, all clinical trial-related documents, such as case records, are recorded and distinguished by the subject identification code (usually the subject's initials) rather than the subject's name.

17.11 이상반응 발생 시 조치

Measures taken when adverse events occur

이상반응 발생 시 즉시 담당의사로부터 필요 검사 및 치료를 받을 수 있도록 관리한다. 중대한 이상반응 발생 시에는 시험을 중지하고 15.3.3항의 이상반응 발생 시 조치사항에 따라 신속하고 적절한 조치를 취한다.

If an unfavorable event occurs, ensure that the subject receives the necessary tests and treatment from a doctor as soon as possible. If a serious adverse event occurs, the test should be stopped and prompt and appropriate action should be taken in accordance with the measures in case of adverse event described in Section 15.3.3.

17.12 자료의 보관 Storage of data

임상시험 실시와 관련된 각종 자료 및 기록을 잘 보존하도록 보관하는 장소가 따로 준비되어 있고 보안을 유지하도록 한다. 결과보고서 작성 이후에는 임상시험 관련 문서를 임상시험 실시기관 문서보관실에 임상시험 완료일로부터 3년간 보관한다.

To ensure that various data and records related to the conduct of clinical trials are well preserved, a separate storage location should be prepared and security should be maintained. Documents pertaining to the clinical trial are stored in the archives of the clinical trial institution for three years following the completion of the clinical trial.

18. 임상시험 담당자 (Clinical trial person in charge)

[별첨1] 임상시험 담당자

[Appendix 1] Clinical trial person in charge

19. 피해자 보상에 대한 규약 (Protocol for Victim Compensation)

[별첨2] 피해자 보상에 대한 규약

[Appendix 2] Rules for Victim Compensation

20. 시험 대상자 설명문 및 동의서 (Test subject explanation and consent form)

[별첨3] 시험 대상자 설명문 및 동의서

[Appendix 3] Test subject explanation and consent form

21. 증례기록지 (Case report form)

[별첨4] 증례기록지

[Appendix 4] Case report form

22. 모집 광고문 (Recruitment advertisement)

[별첨5] 모집 광고문

[Appendix 5] Recruitment advertisement

23. 참고문헌 (References)

1) Bum Gwon CH. Analysis of Gait Characteristics Using GaitRite System in Children with Down Syndrome. *Journal of Adapted Physical Activity and Exercise*. 2011;19(4):123-34.
2) Hwang-bo G, Jeong HY, Bae SS. Comparison of Gait Characteristics in Young and Old Persons with GAITRite System Analysis. *PNF and Movement*. 2003;1(1):33-41.
3) JY H. The effect of manual acupuncture therapy on symptoms of the patients with idiopathic Parkinson's disease. *Korean J Orient Med*. 2003;24:172-83.
4) Lei H, Toosizadeh N, Schwenk M, Sherman S, Karp S, Sternberg E, Najafi B. A pilot clinical trial to objectively assess the efficacy of electroacupuncture on gait in patients with Parkinson's disease using body worn sensors. *PLOS One*. 2016;11(5):e0155613.
5) Kang MK, Lee SH, Hong JM, Park SM, Kang JW, Park HJ, Lim S, Chang DI, Lee YH. Effect of electroacupuncture on patients with idiopathic Parkinson's disease. *Journal of Acupuncture Research*. 2004;21(5):59-68.
6) Chung KA, Lobb BM, Nutt JG, Horak FB. Effects of a central cholinesterase inhibitor on reducing falls in Parkinson disease. *Neurology*. 2010;75(14):1263-9.
7) Lee MS, Park YG, Bae NY. A Case Study of a Taeeumin Patient with Advanced Parkinson's Disease Having Orthostatic Hypotension. *Journal of Sasang Constitutional Medicine*. 2016;28(2):193-204.
8) Toosizadeh N, Lei H, Schwenk M, Sherman SJ, Sternberg E, Mohler J, Najafi B. Does integrative medicine enhance balance in aging adults? Proof of concept for the benefit of electroacupuncture therapy in Parkinson's disease. *Gerontology*. 2014;61(1):3-14.
9) Cho SY, Shim SR, Rhee HY, Park HJ, Jung WS, Moon SK, Park JM, Ko CN, Cho KH, Park SU. Effectiveness of acupuncture and bee venom acupuncture in idiopathic Parkinson's disease. *Parkinsonism & Related Disorders*. 2012;18(8):948-52.
10) Fietzek UM, Schroeteler FE, Ziegler K, Zwosta J, Ceballos-Baumann AO. Randomized cross-over trial to investigate the efficacy of a two-week physiotherapy programme with repetitive exercises of cueing to reduce the severity of freezing of gait in patients with Parkinson’s disease. *Clinical Rehabilitation*. 2014;28(9):902-11.
11) Lee YE, Lee DH, Lee JH, Lu HY, Cho SY, Park JM, Ko CN, Bae HS, Park SU. Three case reports of postural instability and gait difficulty in parkinson's disease patients treated with korean and western medicine. *Korean Journal of Acupunct*. 2014;31(1):40-7.
12) Dorsey ER, Elbaz A, Nichols E, Abbasi N, Abd-Allah F, Abdelalim A, Adsuar JC, Ansha MG, Brayne C, Choi JY, Collado-Mateo D. Global, regional, and national burden of Parkinson's disease, 1990–2016: a systematic analysis for the Global Burden of Disease Study 2016. *The Lancet Neurology*. 2018;17(11):939-53.
13) Lee JJ. Pharmacological treatment in Parkinson’s disease. *Journal of the Korean Neurological Association*. 2019;37(4):335-44.
14) Kalilani L, Friesen D, Boudiaf N, Asgharnejad M. The characteristics and treatment patterns of patients with Parkinson’s disease in the United States and United Kingdom: A retrospective cohort study. *PLOS One*. 2019;14(11):e0225723.
15) Voges J, Hilker R, Bötzel K, Kiening KL, Kloss M, Kupsch A, Schnitzler A, Schneider GH, Steude U, Deuschl G, Pinsker MO. Thirty days complication rate following surgery performed for deep-brain-stimulation. *Movement Disorders*. 2007;22(10):1486-9.
16) van der Kolk NM, King LA. Effects of exercise on mobility in people with Parkinson's disease. *Movement Disorders*. 2013;28(11):1587-96.
17) Mak MK, Wong-Yu IS, Shen X, Chung CL. Long-term effects of exercise and physical therapy in people with Parkinson disease. *Nature Reviews Neurology*. 2017 Nov;13(11):689-703.
18) 한의표준임상진료지침개발사업단 (Korean Medicine Standard Clinical Practice Guidelines Development Team). (2020). 파킨슨병 한의표준임상진료지침 (Parkinson’s Disease: Clinical

Practice Guideline of Korean Medicine).
